# Model-Based Quantification of Left Ventricular Diastolic Function in Critically Ill Patients with Atrial Fibrillation from Routine Data: A Feasibility Study

**DOI:** 10.1155/2019/9682138

**Published:** 2019-05-16

**Authors:** Nicholas Kiefer, Maximilian J. Oremek, Andreas Hoeft, Sven Zenker

**Affiliations:** ^1^Department of Anesthesiology & Intensive Care Medicine, University of Bonn Medical Center, Bonn, Germany; ^2^Department of Anesthesiology, Intensive Care Medicine and Pain Treatment, Hospital Dortmund, Dortmund, Germany; ^3^Applied Mathematical Physiology (AMP) Lab, Department of Anesthesiology & Intensive Care Medicine, University of Bonn Medical Center, Bonn, Germany

## Abstract

**Introduction:**

Left ventricular diastolic dysfunction (LVDD) and atrial fibrillation (AF) are connected by pathophysiology and prevalence. LVDD remains underdiagnosed in critically ill patients despite potentially significant therapeutic implications since direct measurement cannot be performed in routine care at the bedside, and echocardiographic assessment of LVDD in AF is impaired. We propose a novel approach that allows us to infer the diastolic stiffness, *β*, a key quantitative parameter of diastolic function, from standard monitoring data by solving the nonlinear, ill-posed inverse problem of parameter estimation for a previously described mechanistic, physiological model of diastolic filling. The beat-to-beat variability in AF offers an advantageous setting for this.

**Methods:**

By employing a global optimization algorithm, *β* is inferred from a simple six parameter and an expanded seven parameter model of left ventricular filling. Optimization of all parameters was limited to the interval ]0, 400[ and initialized randomly on large intervals encompassing the support of the likelihood function. Routine ECG and arterial pressure recordings of 17 AF and 3 sinus rhythm (SR) patients from the PhysioNet MGH/MF Database were used as inputs.

**Results:**

Estimation was successful in 15 of 17 AF patients, while in the 3 SR patients, no reliable estimation was possible. For both models, the inferred *β* (0.065 ± 0.044 ml^−1^ vs. 0.038 ± 0.033 ml^−1^ (*p*=0.02) simple vs. expanded) was compatible with the previously described (patho) physiological range. Aortic compliance, *α*, inferred from the expanded model (1.46 ± 1.50 ml/mmHg) also compared well with literature values.

**Conclusion:**

The proposed approach successfully inferred *β* within the physiological range. This is the first report of an approach quantifying LVDF from routine monitoring data in critically ill AF patients. Provided future successful external validation, this approach may offer a tool for minimally invasive online monitoring of this crucial parameter.

## 1. Introduction

Heart failure (HF) and atrial fibrillation (AF) are both frequent cardiovascular conditions that share an increasing prevalence and cause significant morbidity, mortality, and socioeconomic burden [1–5]. Incidence correlates with age [6–8]. In the USA, HF is the most frequent reason for unplanned/medical ICU admission, either primarily as acute HF or as an aggravating comorbidity [9].

About half of HF patients present with left ventricular diastolic dysfunction (LVDD) [5, 10, 11], a condition termed “heart failure with preserved ejection fraction” (HFpEF). LVDD and AF are closely connected by pathophysiology and prevalence: LVDD precipitates AF, and the absence of atrial contraction in AF aggravates LVDD [12–15]. AF is the most common arrhythmia in HF with 65% of HFpEF presenting with AF [16]. AF is associated with increased mortality in HFpEF [17]. Although in cardiology, HFpEF has become a very common diagnosis, LVDD remains underdiagnosed in the ICU population, despite a potentially large impact on therapeutic decisions [18].

In critically ill patients, prevalence of AF is increased [19] and left ventricular diastolic function (LVDF) may be additionally impaired [20]. Assessment and monitoring of LVDF in presence of AF is challenging as standard echocardiographic measures (*E*/*A*, *E*/*E*′) are difficult to obtain and do not allow for continuous monitoring [21]. Acquisition of invasive measures such as the left ventricular diastolic time constant (*τ*) is not feasible at the bedside [22].

The fundamental cardiovascular physiology approach to quantify diastolic cardiac function involves the recording of pressure-volume loops under varying preload conditions to estimate the nonlinear diastolic pressure-volume relationship [23]:

(1) $$\begin{array}{c} P_{\text{LV}}\left(V_{\text{LV}}\right)=P_{\text{LV0}}\left(e^{\beta \left(V_{\text{LV}}-V_{\text{ED0}}\right)}-1\right), \end{array}$$

where *β* quantifies the exponential diastolic ventricular stiffness, *P*_LV0_ represents the pressure scaling factor, and *V*_ED0_ corresponds to the unstressed volume.

According to Frank–Starling's law and given an otherwise stationary state, variations in end-diastolic volume (EDV) determine beat-to-beat stroke volume variability [24]. In the absence of synchronised atrial contraction in AF, EDV primarily depends on the duration of filling, the diastolic properties of the ventricle, preload, and the end-systolic volume, which in turn depends on the previous cardiac cycle and the systolic properties of the ventricle. Previous attempts to quantify the relationship between variable filling times in AF and stroke volume have produced heterogeneous results [25].

The application of nonlinear mathematical representations of physiology based on solving the inverse problem of parameter estimation from available observations/measurements has become feasible at the bedside due to the recent availability of affordable high-performance computational resources [26, 27]. For such a model-based approach, AF patients are a particularly promising population due to the high intrinsic variability in observable physiological time series. This variability effectively acts as a continuous high-bandwidth perturbation of the underlying biological system, facilitating system identification and subsequent parameter estimation within a short observation period.

Zenker et al. have previously described a simple, nonlinear, mechanistic model of the left ventricle [28]. We hypothesized that inversion of this model would allow quantitative inference of parameters of LVDF from routine ECG and invasive arterial pressure (ABP) recordings in AF patients under minimal assumptions. The aim of this study was to assess whether inference of *β*, the exponential diastolic ventricular stiffness, is feasible using global optimisation. Additionally, we aimed to evaluate whether uncertainty quantification from the local covariance would allow for robust identification of individual patients in which inference failure occurred.

## 2. Materials and Methods

### 2.1. Ethical Approval

The dataset used in this study is freely available and fully deidentified. Thus, no ethical restrictions apply in this context.

### 2.2. Data Acquisition

We used the PhysioNet [29] Massachusetts General Hospital/Marquette Foundation (MGH/MF) Waveform Database [30]. It contains recordings of patient's physiological signals including ECG and invasive ABP. The data are 360 Hz 8-bit time series in the PhysioNet binary format. Patients were selected using the patient guide, which contained limited clinical information such as cardiac rhythm. We screened the entire database with regard to documented cardiac rhythm and extracted datasets for two patient groups: the AF group (17 patients) included all patients with AF without pacemakers or intermittent non-AF cardiac rhythms in the available low-noise areas of the recordings. The sinus rhythm (SR) control group included 3 patients with documented SR without ECG alterations or an arrhythmia described in the patient guide.

### 2.3. Signal Processing and Data Selection

RR intervals (RRIs) were calculated based on an ECG R peak detection algorithm derived from methods described by Arzeno and coworkers [31]. Pulse pressures (PPs) were computed from ABP recordings using an algorithm adapted from Zong et al. [32]. Manual selection of low-noise intervals was then performed to extract 800 pairs of RRIs followed by pulse pressures (PPs). The resulting RRI and PP time series are made available as text files in the online supplement.

### 2.4. Software Development and Implementation

The main data processing and inference routine was implemented in C++ using the ADOL-C 2.63 [33], Armadillo v8.2 [34], Boost v1.60 [35], and TRNG4 4.19 [36] libraries. The data were visualised and postprocessed using Python v.3.6 [37] with the numpy v.1.13 [38], Matplotlib v.2.1 [39], pandas v.0.19 [40], and SciPy v1.0 [41] packages. The estimation of the covariance used MATLAB R2017a, (The Mathworks, Inc, Natick, Massachusetts, USA). The analysis code is available in the online supplement Code.

### 2.5. Mathematical Model

The mechanistic model of the left ventricle used in this paper derives the diastolic filling behaviour from a simple ODE:

(2) $$\begin{array}{c} V_{\text{ED}}\left(t_{\text{dia}}\right)=k_{3}\left(t_{\text{dia}}+C\right)-\frac{1}{\beta }\text{ln}\left(\frac{1-k_{1}e^{\beta k_{3}\left(t+C\right)}}{k_{3}}\right), \end{array}$$

whose solution predicts end-diastolic volume, *V*_ED_, from filling times, *t*_dia_, the exponential diastolic stiffness parameter, *β*, the time shift constant, *C*, and two combined parameters *k*_1_ and *k*_3_, which in addition to *β*, the mitral valve resistance, *R*_valve_, and the filling pressure, *P*_cvp_, incorporate parameters *P*_LV0_ and *V*_ED0_ of the nonlinear pressure-volume relationship [28].

(3) $$\begin{array}{c} k_{1}=\frac{-P_{\text{LV0}}}{R_{\text{valve}}}e^{-\beta V_{\text{ED0}}},\\ k_{3}=\frac{P_{\text{LV0}}}{R_{\text{valve}}}+\frac{P_{\text{cvp}}}{R_{\text{valve}}}, \end{array}$$

where *k*_1_ and *k*_3_ both include the term *P*_LV0_/*R*_valve_, with *k*_1_ additionally depending on *β* and *V*_ED0_. This interdependence warrants constraint considerations for valid combinations when solving the inverse problem. More information on the model parameters and the limits is provided in the online supplement Expanded Methods-Modeling and Constraints (available here).

The model predicts volumes, whereas in routine data, only pressure measurements are readily available. To convert volumes into pressures, we assume a linear model of the static aortic pressure-volume relationship, expressing pulse pressures as a function of diastolic filling time *t*_dia_ with the aortic compliance, *α*, and the zero-offset, *δ*:

(4) $$\begin{array}{c} PP_{i}=\frac{V_{\text{ED}}\left(t_{\text{dia }i}\right)}{\alpha }+\delta . \end{array}$$

Equations (2) and (4) jointly define the simple model (SM), in which only the diastolic properties of the left ventricle and aortic pressure-volume relationship are taken into account.

After fitting the SM resulting from equation (4) to patient data, we observed a strong systematic dependence of residuals on the RRI preceding the RRI directly determining the filling duration. Such a dependence has previously been attributed to variations of the inotropic state of the heart, among other things [42, 43]. The model was therefore expanded to include a simple linear correction to compensate these effects. The pulse pressure of the *i* ^th^ beat for this expanded model (EM) then depends on an additional factor *k*_4_ weighting the contribution of the prepreceding RRI:

(5) $$\begin{array}{c} PP_{i}=\frac{V_{\text{ED}}\left(t_{\text{dia }i}\right)+k_{4}\;*\;t_{\text{dia }i-1}}{\alpha }+\delta . \end{array}$$

### 2.6. Global Optimisation

Since initial exploration showed that efficient local optimization algorithms of various types became trapped almost immediately in the local minima in the neighbourhood of randomly chosen starting points, we were forced to fall back to parallel tempering (PT) as a computationally expensive global optimization technique to obtain estimation results independent of subjective choices of starting points [44]. The likelihood was calculated assuming a normally distributed, independent measurement error with a standard deviation (SD) of 12 mmHg for PPs. To minimize the potential influence of assumptions on results, we constrained optimization for all parameters to ]0, 400[, a range much larger than known or reasonable physiological constraints. Additionally, the optimization was initialised randomly, rejecting randomly chosen starting points if the local log-likelihood did not exceed a threshold of −5208, an empirically chosen limit corresponding to the likelihood at a sum of squares error of 1,500,000 mmHg^2^. This was done to ascertain initialisation of the PT on the support of the likelihood function to obtain appreciable acceptance rates. Based on experience from exploratory sampling runs to determine the support of the likelihood function, the following initialization intervals were chosen: *k*_1_, *β*, and *C* by a uniform distribution in log space ]−40, 4[, *k*_3_ by a uniform distribution ]120, 400[, *α* by a uniform distribution ]0, 5[, and *δ* by a uniform distribution ]20, 80[.

We ran 40 million PT samples using 24 chains, with a randomised starting point for each chain within the support and a multivariate Gaussian proposal distribution with a diagonal, empirically determined covariance fixed across all patients. The chains were separated by an exponential temperature ladder with base 6. The code, data, and instructions are made available in the online supplement (available here).

### 2.7. Statistics/Uncertainty Quantification

The best (maximum) likelihood vector (BLV) seen by the sampler was used to compute Pearson's *R* ^2^ as a measure of observed fraction of variation explained (FVE) by the model. At this parameter vector, the uncertainty was calculated by estimating the covariance from a local Hessian, regularized following the recommendations of Gill and King [45]. Details are discussed in the online supplement Expanded Methods-Uncertainty Quantification (available here).

The residuals with respect to the filling and prefilling interval were evaluated for any systematic errors. All data are presented as mean ± standard deviation (SD). Groups were compared using the Mann–Whitney rank sum test, and a two-tailed *p* < 0.05 was considered statistically significant [46].

## 3. Results

### 3.1. Patient Population

The analysis included 17 AF and 3 SR patients with mean age 72.5 ± 9.79 yrs and 37.3 ± 25.8 yrs, respectively. The SR group did not have any documented preexisting cardiovascular diseases and included two patients with orthopaedic/trauma diagnosis and one with sepsis. The AF group included 10 patients with various cardiovascular primary diagnoses, four patients with gastrointestinal diagnoses, and one patient each with sepsis, cerebral haemorrhage, and renal calculus with retroperitoneal haematoma.

Mean RRIs and PPs of the individual patient did not differ significantly between AF and SR groups (0.68 ± 0.11 s vs. 0.76 ± 0.04 s, *p*=0.20; 71.1 ± 17 mmHg vs. 57.8 ± 18 mmHg, *p*=0.20). Individual variability expressed as SD of RRIs and PPs was significantly higher in the AF group compared to the SR group (SD of RRI 0.12 ± 0.04 s vs. 0.024 ± 0.01 s, *p* < 0.005; SD of PP 12.45 ± 5.65 mmHg vs. 2.89 ± 0.37 mmHg, *p* < 0.005). Within the AF group, two patients (Patient IDs: mgh013 and mgh130) displayed a low variability of RRI (SD of RRI 0.06 s and 0.048 s, respectively) similar to the SR group.

### 3.2. Population Level Estimation Results: Goodness of Fit and Uncertainty Estimation

**Figure 1:**
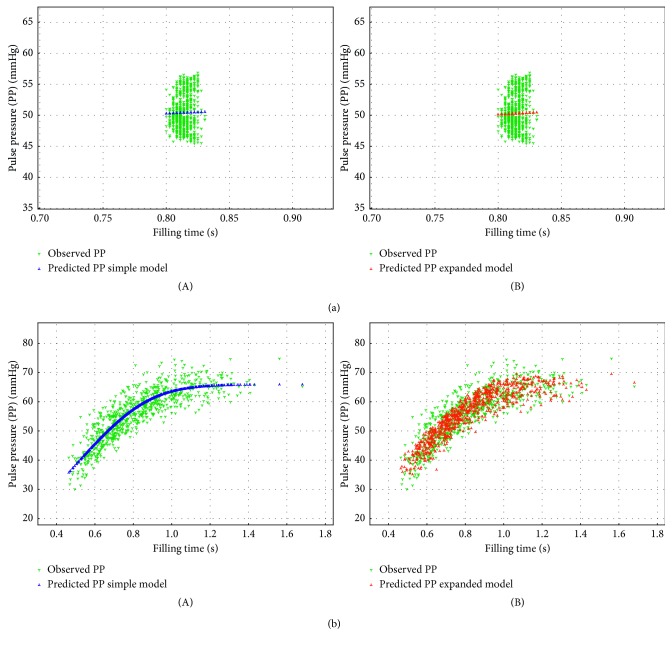
Observed (green) vs. predicted (blue for the simple model, red for the expanded model) relationship between pulse pressures (PPs) and filling times for a typical sinus rhythm patient (mgh079) (a) and a typical atrial fibrillation patient (mgh126) (b). (A) Simple model. (B) Expanded model.

The BLVs for both simple model (SM) and expanded model (EM) provided excellent fits to the observed RRI-PP relationships in 15 of 17 AF patients, with the EM largely eliminating the systematic dependency on the RRI preceding the RRI determining the filling time. FVE was 0.53 ± 0.17 vs. 0.70 ± 0.21, *p*=0.007 for SM and EM, respectively. For SR patients, both models were unable to provide adequate fits, as evidenced by the low values of FVE (0.017 ± 0.013 vs. 0.062 ± 0.04, *p*=0.38). Examples are provided in Figure 1, showing a side-by-side comparison of the models for a typical sinus and AF patient. Plots of all patients are available in the online supplement Expanded Results (available here).

BLV values of *β* in AF patients were 0.065 ± 0.044 ml^−1^ vs. 0.038 ± 0.033 ml^−1^ (*p*=0.02) for SM and EM, respectively. The EM values of 15 out of 17 AF patients were within the range reported by Kass et al. (0.044 ± 0.024 ml^−1^) [47], whilst on average slightly exceeding the range described by Schmitt et al. (0.023 ± 0.006 ml^−1^) [48], whose measurements were performed in a younger and healthier cohort, who may quite plausibly present with a more compliant ventricle. For all SR patients, values of *β* were in a similar but higher range (0.096 ± 0.049 ml^−1^ vs. 0.11 ± 0.016 ml^−1^ *p*=0.66).

The only other estimated parameter for which we were able to find literature values is capacitive aortic compliance in ml/mmHg. This was reported in the study by Cohn et al., Duprez et al., Liu et al., and McVeigh et al. [49–52] ranging from 0.8 ml/mmHg to 2.2 ml/mmHg. SM and EM showed values of 3.22 ± 2.11 ml/mmHg vs. 1.46 ± 1.50 ml/mmHg (*p*=0.04), respectively, for AF patients. The inferred aortic compliance values of the expanded, but not the simple, model thus compared well with the physiological range.

**Figure 2:**
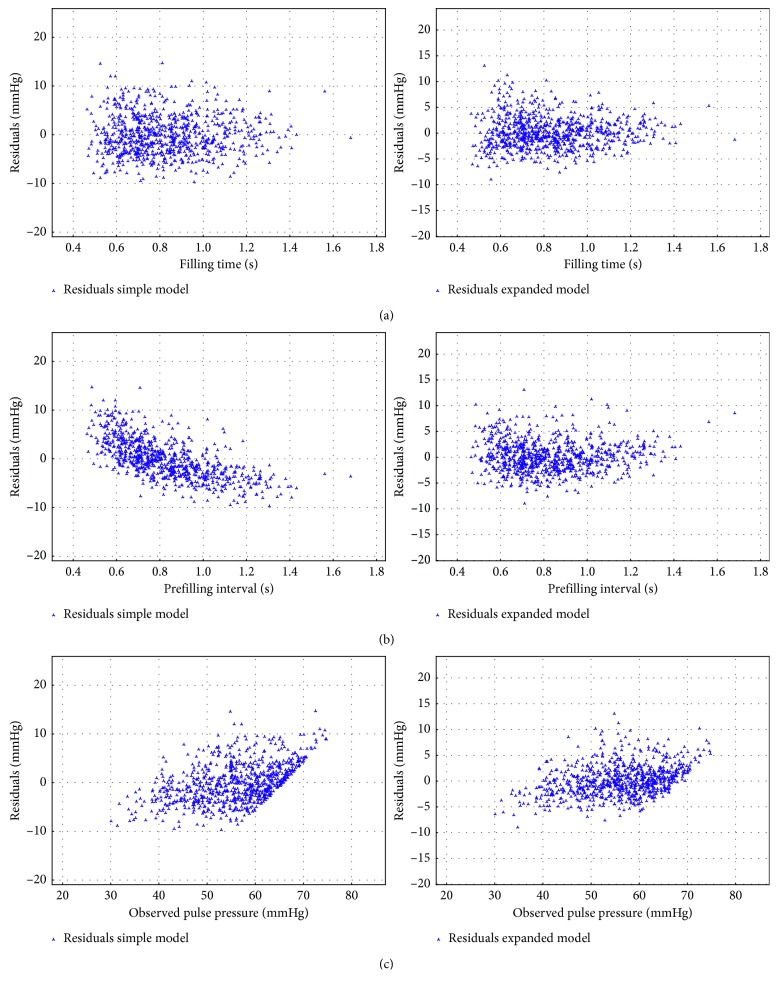
Residuals with respect to filling interval, prefilling interval, and observed pulse pressures for mgh126, a typical AF patient: residuals with respect to the (a) filling interval for the simple and expanded model, (b) RR interval prior to the filling interval for the simple and expanded model, and (c) observed pulse pressure for the simple and expanded model.

For both models, the residuals with respect to filling time, prefilling interval, and observed pulse pressure were inspected. A typical example for an AF patient is shown in Figures 2(a)–2(c). For both models, the residuals with respect to filling time appear to be randomly distributed. In the residuals with respect to the prefilling interval, the dependence seen when applying SM is no longer present for the EM. In the case of dependence of residuals on the observed pulse pressure, SM shows a stronger correlation than the EM.

The parameters discussed and their estimated individual errors, as well as the FVE for all individual patients, are summarised in Table 1.

### 3.3. Individual Inference Results: Identifying Inference Failure

**Figure 3:**
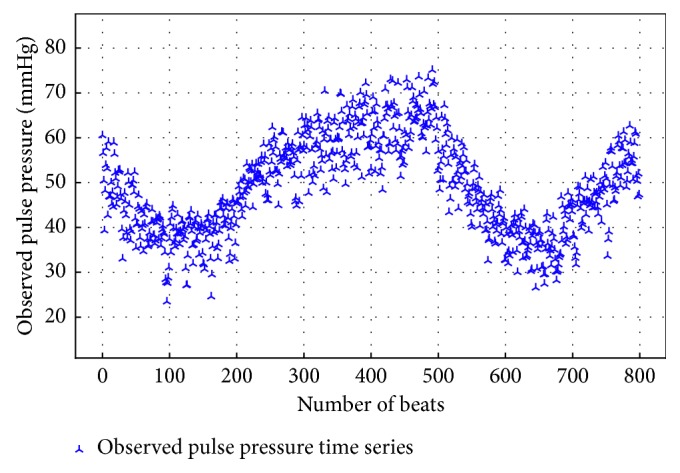
Example of the nonstationary time series of pulse pressures in patient mgh145, leading to inference failure due to violation of modeling assumptions.

The FVE values in Table 1 allow the identification of one patient (identifier: mgh145) for which both models are not able to explain more than 13% of the observed variation. Further investigation of this patient showed that the measurement data violated the model assumption of stationarity, as seen in Figure 3.

**Figure 4:**
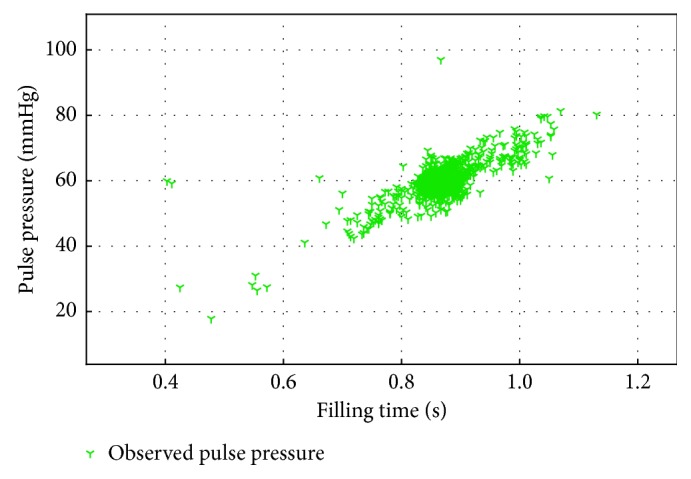
Example of atrial fibrillation patient (mgh130) without nonlinear plateau of pulse pressures for higher filling times, leading to estimation of a degenerate linear rather than the informative nonlinear model.

Another outlier can be seen in the values of *β* for patient mgh130. Whilst this patient shows values within the physiological range for the simple model, the expanded model shows an extreme value of 0.005 ml^−1^, which is an entire order of magnitude smaller than the expected range. Looking more closely at the input data, the patient shows a linear trend without the nonlinear plateau seen in the plot of pulse pressure vs. filling time as is present in the other AF patients.  Figure 4 shows the plot for mgh130 as compared to typical AF patients in Figure 1.

A further tool for identification of model failure was tested through estimation of the error associated with the parameter vector from the local Hessian. The values are shown in Table 1 for the estimated error on *β*. Comparison between simple and expanded model shows a mean value across all patients of 0.03 ± 0.03 ml^−1^ vs. 0.009 ± 0.008 ml^−1^ (*p*=0.01), suggesting a significantly smaller estimation uncertainty in the expanded model, in addition to the larger FVE. Similarly, comparing the error on the aortic compliance, *α*, yields 4.54 ± 4.31 ml/mmHg vs. 1.38 ± 1.48 ml/mmHg, *p* < 0.05. Unfortunately, the estimation of the covariance did not flag the previously described model failures or highlight any new AF patients when looking at the errors on *β* or *α*.

To summarize, we were able to identify a measure of the diastolic ventricular stiffness within the physiological range for both models from routine data using a mechanistic mathematical model. The simple model described only diastolic features of the left ventricle but produced a strong dependence in residuals with respect to the prefilling interval. An expansion of the model using a correcting term, which takes the RR interval prior to filling into account, not only largely eliminated this dependency but additionally provided estimates of aortic compliance within the physiological range.

## 4. Discussion

### 4.1. Inference Results

Our hypothesis was that model-based quantitative inference would enable us to determine exponential diastolic ventricular stiffness, *β*, an important quantitative parameter of diastolic cardiac function, from ECG and ABP measurements in patients with AF, but not in patients with sinus rhythm. The probability that all global estimates with global search initialized across and constrained to a huge interval would by chance almost without exception converge to values within the comparatively tiny physiological range corresponding to approx. 0.05% of the constraint interval appears vanishingly small. Thus, our data clearly support this hypothesis. This is, to our knowledge, the first report of quantitative estimation of diastolic ventricular function from routine clinical monitoring data in patients with AF.

Not only did employing the expanded model result in improved fits as was expected but also allowed inference of an additional parameter, aortic compliance, within the reported physiological range of 0.8 ml/mmHg to 2.2 ml/mmHg. Interestingly, estimation uncertainty was reduced for the expanded model, as well. Whether this is an artefact of the regularized uncertainty estimation procedure in the presence of ill-posedness and thus local noninvertibility of the Hessian at the maximum likelihood estimate or an effect of reduced misspecification remains to be elucidated.

### 4.2. Identifiability of Inference Failure

With respect to potential clinical application, robust and, ideally, automatically determining valid individual inference results is crucial. In this investigation, we observed a case in which the violation of the model assumption of stationarity resulted in an extremely low FVE for both models. In the expanded model, a second outlier was identifiable only through the implausibly low (by an order of magnitude below the physiological range) *β* value. When inspecting the data, we found that this patient did not exhibit the nonlinear plateau seen in the other patients, resulting in the optimization effectively converging to a linear reduced model as *β* approaches zero rather than the full nonlinear model. Neither of these patients were identifiable from the covariance matrix at the point of best likelihood seen by the sampler when inspecting the errors on *β* and aortic compliance, suggesting either a thresholding approach or comparison of FVE with a reduced linear model as possible ways of identifying this type of inference failure induced by insufficient or nonstationary data in practice.

In the SR patients, no adequate fit to the observed pulse pressures was achieved, as evidenced by the low FVE values and supported by visual inspection of fits and residuals. Yet, the inferred values of *β* were only slightly above the physiological range. Inspecting the errors as estimated from the covariance matrix showed larger errors for both *β* and aortic compliance. The actual usefulness of this estimation, however, appears questionable as the model's assumptions are violated by the underlying physiology in sinus patients. Ultimately, only experimental corroboration will determine usefulness in the clinical setting.

When examining residuals for systematic errors and comparing results between the simple and expanded model, it becomes apparent that focusing on the diastolic properties of the ventricle only cannot explain the entire beat-to-beat variability. Taking into account the systematic dependence on the prefilling interval in the expanded model largely resolved this systematic dependence in the residuals but not the systematic dependence of residuals on observed values. This suggests a role for more realistic, physiologically motivated models directly accounting for effects of end-systolic volume, inotropic state, and afterload on ventricular performance.

### 4.3. Clinical Relevance

Our findings, if transferable to the bedside, are of high clinical importance for the large group of ICU patients with combined AF and LVDD.

To our knowledge, no epidemiological data on the combined prevalence of LVDD and AF in critically ill patients exist in the literature, but it is likely to be high. Quantification of LVDD is impaired in presence of AF, while it is of significant clinical importance in critically ill patients. Treatment of acute heart failure in the ICU typically focuses on adjusting systolic function, volume balance, and peripheral resistance, usually based on combining volume therapy with catecholaminergic inotropes and vasopressors. These catecholamines may further impair diastolic function, if their contractile effect outweighs their lusitropic effect, a condition commonly referred to as overstimulation [53]. LVDD plays a key role in overstimulation, and presence of inflammation may further aggravate this mechanism [53]. Milrinone and levosimendan, in contrast, have been shown to improve diastolic function [54–56]. Also, LVDD plays a role in weaning pulmonary edema and weaning failure [57]. Our approach may thus enable automated, continuous, and user-independent assessment of LVDF, providing online guidance for differential intensive care therapy of shock. In the future, the continuous monitoring of LVDD may lead to novel insights into the physiology of diastolic function under volume and catecholaminergic therapy in the ICU setting. It might also contribute to provide better guidance to volume therapy in the AF subpopulation.

### 4.4. Limitations

An issue requiring further investigation is the robust and automatic identification of inference failure. The approach of estimating the covariance from the Hessian, which was not positive definite and thus noninvertible to a classical covariance in this case, did no yield a robust measure of such failure. The noninvertible, nonpositive definite Hessian is a well-known issue especially in the case of nonlinear models with only partial identifiability and has been discussed in depth with various suggested approaches ranging from rethinking the model to employing Bayesian sense sampling techniques [45]. We have been unable to definitively resolve this issue so far. From our experience when attempting to sample full Bayesian posterior distributions for this model to more robustly estimate parameter uncertainty, only complex algorithmic approaches such as parallel tempering with large number of samples combined with manual tuning of the sampling covariance for each individual patient/dataset were able to generate reasonably stable posterior estimates, which of course, aside from not being amenable to full automation in the translational setting, also carry the risk of introducing subjective bias through the manual tuning process. We therefore decided not to report such preliminary results at this stage.

The advantage of robust inference failure identification is the confidence in the quality and reliability of results. Currently, haemodynamic monitoring devices that rely on complex processing algorithms may provide information on the quality of the input signal, e.g., as a signal quality index, but they are often inherently unable to give any information on the quality of the computed output. This makes such monitors a black box for the clinician, who has to use the displayed parameters for clinical decisions without information on their reliability in the current clinical situation.

In this retrospective study, independent validation measurements of the inferred *β* were not available. Therefore, this preliminary report can only serve to stimulate further research using prospective data that contain quantitative measurements of diastolic function, and data collected during interventions to validate our findings. External validation is also required to assess whether the inferred *β* values may be usable in situations of estimation failure and in SR patients, given that estimation results also approach the physiological range in this pilot study. With regard to inference methodology, the current approach using parallel tempering as a global optimisation routine is limited to relatively short stationary time series, which could be addressed by applying sequential Monte Carlo techniques [58] to allow for online estimation of time-varying parameters by sequentially assimilating incoming measurements. For bedside use of such an approach, robust identification of model failure in the case of noisy input data is required and future work needs to address this.

A further limitation is the small number of SR patients matching the inclusion criteria, resulting in a significantly younger and healthier SR group. This, however, does not question the findings. The purpose of including an SR group in the study design was to demonstrate that the approach only works with broadband beat-to-beat variation as seen in AF, not in SR, and that its failure can clearly be identified.

### 4.5. Outlook and Future Work

The observation of better fits from the expanded model with reduced estimation uncertainty, along with reduced, but not eliminated systematic dependencies in residuals, suggests applying a physiologically more accurate model of left ventricular function, which includes a quantitative description of systole.

Such a more complex approach will require further investigation of sampling techniques and algorithms as the current simple model already proved challenging to estimate. We believe this to be due to the highly nonlinear underlying parameter space, which could only be effectively sampled by the PT algorithm. Sequential Monte Carlo techniques may work better if employed in a continuous tracking scenario, as is required for clinical application, but the fundamental challenge of estimation from distributions with narrow support on a curved submanifold, which appears as the primary source of the estimation difficulty, will remain.

## 5. Conclusion

In this study, we demonstrated for the first time that inferring diastolic ventricular stiffness, *β*, from routine monitoring data may be possible in ICU patients presenting with AF. This is of potentially high clinical importance for the large group of ICU patients with combined LVDD and AF because of relevant therapeutic implications. Uncertainty estimation, via local evaluation of the covariance from the Hessian, in the setting of an ill-posed, nonlinear problem did not allow for robust identification of inference failure. In order to translate the methodology to the bedside, further work with regard to robust and automated identification of inference failure and continuous parameter estimation is needed. A crucial next step will be the prospective validation of the quantitative correctness of the inferred parameter values from clinical information.

## Figures and Tables

**Table 1:** Summary of patient characteristics and results for both models. The results were rounded to three significant figures where applicable.

Patient	Age	Rhythm	Mean RR ± SD (s)	Mean PP ± SD (mmHg)	FVE simple model	*β* simple model ± EE(ml^−1^)	*α* simple model ± EE(ml/mmHg)	FVE expanded model	*β* expanded model ± EE(ml^−1^)	*α* expanded model (ml/mmHg)
mgh013	73	AF	0.787 ± 0.06	72.1 ± 7.89	0.36	0.0755 ± 0.0215	3.86 ± 3.58	0.48	0.0136 ± 0.00205	0.711 ± 0.621
mgh019	79	AF	0.75 ± 0.11	46.5 ± 12.9	0.5	0.0649 ± 0.104	3.71 ± 8.11	0.82	0.150 ± 0.0244	0.684 ± 0.237
mgh023	78	AF	0.794 ± 0.19	108.3 ± 18.3	0.73	0.0347 ± 0.0050	2.80 ± 2.14	0.91	0.0124 ± 0.00113	1.25 ± 0.985
mgh027	73	AF	0.671 ± 0.175	70.6 ± 19.1	0.45	0.0700 ± 0.0343	4.07 ± 2.48	0.64	0.0471 ± 0.0104	1.70 ± 0.594
mgh032	78	AF	0.733 ± 0.15	76.0 ± 15.6	0.82	0.0378 ± 0.0103	3.01 ± 3.39	0.93	0.0198 ± 0.00415	0.880 ± 2.36
mgh105	86	AF	0.628 ± 0.16	51.5 ± 8.70	0.53	0.128 ± 0.0655	7.60 ± 11.5	0.73	0.0368 ± 0.00916	5.37 ± 3.88
mgh126	46	AF	0.830 ± 0.203	55.9 ± 8.47	0.77	0.0218 ± 0.0051	4.70 ± 5.87	0.87	0.0335 ± 0.00855	4.98 ± 4.13
mgh129	63	AF	0.420 ± 0.08	80.8 ± 22.0	0.51	0.0574 ± 0.0269	1.03 ± 2.68	0.74	0.0472 ± 0.00516	0.529 ± 0.398
mgh130	73	AF	0.856 ± 0.05	59.7 ± 4.06	0.5	0.105 ± 0.0621	5.62 ± 10.38	0.74	0.0052 ± 0.00112	0.622 ± 0.177
mgh135	65	AF	0.549 ± 0.11	88.9 ± 11.8	0.44	0.0523 ± 0.0089	0.324 ± 0.432	0.47	0.0553 ± 0.0220	0.333 ± 0.463
mgh139	64	AF	0.608 ± 0.13	82.4 ± 13.8	0.7	0.0387 ± 0.0084	2.33 ± 1.41	0.834	0.0187 ± 0.00796	0.652 ± 0.749
mgh141	73	AF	0.714 ± 0.14	56.9 ± 12.7	0.41	0.0125 ± 0.0034	0.252 ± 0.069	0.58	0.0112 ± 0.00237	0.461 ± 0.208
mgh144	71	AF	0.735 ± 0.12	74.1 ± 7.69	0.52	0.0538 ± 0.0137	5.64 ± 2.27	0.76	0.0441 ± 0.0112	2.40 ± 3.69
mgh145	81	AF	0.578 ± 0.09	49.1 ± 10.6	0.13	0.0459 ± 0.0062	0.482 ± 0.151	0.14	0.0672 ± 0.0286	2.57 ± 3.85
mgh146	87	AF	0.637 ± 0.08	97.7 ± 8.94	0.34	0.0736 ± 0.0388	3.59 ± 14.83	0.46	0.0347 ± 0.00548	0.560 ± 0.260
mgh147	76	AF	0.533 ± 0.11	70.1 ± 24.6	0.61	0.0340 ± 0.0092	0.511 ± 0.481	0.86	0.0328 ± 0.00424	0.211 ± 0.215
mgh149	67	AF	0.706 ± 0.09	78.7 ± 8.15	0.64	0.194 ± 0.0845	5.18 ± 7.46	0.89	0.0110 ± 0.00173	0.955 ± 0.596
mgh059	25	S	0.717 ± 0.04	44.7 ± 2.73	0.02	0.160 ± 0.516	44.89 ± 177	0.09	0.118 ± 0.0823	19.8 ± 12.2
mgh079	20	S	0.813 ± 0.02	50.4 ± 2.64	0	0.0862 ± 0.241	10.93 ± 9.51	0.01	0.124 ± 0.0942	31.4 ± 24.6
mgh152	67	S	0.764 ± 0.02	78.4 ± 3.33	0.03	0.0409 ± 0.0226	27.7 ± 51.0	0.1	0.0880 ± 0.211	4.71 ± 3.12

AF = atrial fibrillation; S = sinus; RR = ECG R‐peak intervals; PP = pulse pressure; SD = standard deviation; FVE = fraction of variation explained; *β* = left ventricular diastolic stiffness; *α* = aortic compliance; EE = error estimate.

## Data Availability

The waveform data supporting this numerical analysis are from previously reported datasets, which have been cited and are freely available at PhysioNet.org. The processed input data and algorithm are available in the online supplement and upon request to PD Dr. Sven Zenker.

## Abbreviations

HF: Heart failure
AF: Atrial fibrillation
LVDD: Left ventricular dysfunction
HFpEF: Heart failure with preserved ejection fraction
LVDF: Left ventricular diastolic function
EDV: End-diastolic volume
ABP: Invasive arterial blood pressure
SR: Sinus rhythm
RRI: RR intervals
PP: Pulse pressure
SM: Simple model
EM: Expanded model
PT: Parallel tempering
BLV: Best (maximum) likelihood vector
FVE: Fraction of variation explained
SD: Standard deviation

### Model Parameters

*α*: Aortic compliance
*β*: Left ventricular diastolic stiffness
*δ*: Offset factor (linear compliance)
*k*_1_/*k*_3_: Combined factors of model
*C*: Factor from integration
*P*_cvp_: Left ventricular filling pressure
*P*_LV0_: Pressure scaling factor of pressure-volume equation
*R*_valve_: Mitral valve resistance
*V*_ED0_: Unstressed volume factor of pressure-volume equation.

## Supplementary Materials

Supplementary Materials Online Supplement Expanded Methods and Results: this document provides further details on methodological considerations and details about the model employed. Furthermore, the Results section includes figures of the entire cohort corresponding to those displayed in the manuscript for a selection of patients. Online Supplement Code: this is the product of the research and enables the reader to verify results and potentially apply the methodology to their data. We have a strong commitment to open sourcing the analysis code to promote transparency and reproducibility and thus further future research in this area. This supplement provides the complete analysis code and processed data for the research presented within this manuscript. Within the ZIP folder, the file “Readme.md” provides instructions for building and running the code. If additional questions or issues arise with regard to usage of the code, do not hesitate to contact the authors.
