# Supplementary material for: Model-Based Quantification of Left Ventricular Diastolic Function in Critically Ill Patients with Atrial Fibrillation from Routine Data: A Feasibility Study
Source: Comput Math Methods Med. 2019 May 16;2019:9682138. doi: 10.1155/2019/9682138 (PMC6541946; doi:10.1155/2019/9682138)
Supplement: Supplementary Materials — Online Supplement Expanded Methods and Results: this document provides further details on methodological considerations and details about the model employed. Furthermore, the Results section includes figures of the entire cohort corresponding to those displayed in the manuscript for a selection of patients. Online Supplement Code: this is the product of the research and enables the reader to verify results and potentially apply the methodology to their data. We have a strong commitment to open sourcing the analysis code to promote transparency and reproducibility and thus further future research in this area. This supplement provides the complete analysis code and processed data for the research presented within this manuscript. Within the ZIP folder, the file “Readme.md” provides instructions for building and running the code. If additional questions or issues arise with regard to usage of the code, do not hesitate to contact the authors. [file 9682138.f1.zip › OnlineMethodsAndResults_CMMM_2711431/kiefer_et_al_expanded_methods_and_results.pdf]

Online supplement to "Model-based quantification of left ventricular diastolic function in critically ill patients with atrial fibrillation from routine data: a feasibility study"

Nicholas Kiefer [1,3 *], Maximilian J. Oremek [2 *], Andreas Hoeft [1], Sven Zenker [1,2]

1- Department of Anesthesiology & Intensive Care Medicine, University of Bonn Medical Center, Bonn, Germany

2- Applied Mathematical Physiology (AMP) Lab, Department of Anesthesiology & Intensive Care Medicine, University of Bonn Medical Center, Bonn, Germany

3- Department of Anesthesiology, Intensive Care Medicine and Pain Treatment, Hospital Dortmund, Dortmund, Germany

* - Both authors contributed equally to the research presented in this paper.

Expanded Methods

Modeling and Constraints

This section will give an overview of the model and the parameters, before looking more closely at limit considerations in special cases.

The model is derived from the consideration of the left ventricle as an elastic compartment, which is filled passively dependent on the pressure difference across the mitral valve as described by Equation 6. This difference is dependent upon the filling pressure P_{cvp}, the passive pressure-volume relationship given by equation 1 from [23] and the resistance of the mitral valve, R_{valve}. In equation 1, P_{LV0} represents the pressure scaling factor and V_{ED0} represents the unstressed volume of the ventricle i.e. the volume at which pressure in the ventricle is zero.

$$\frac{dV}{dt} = \frac{P_{CVP} - P_{LV}(V)}{R_{valve}} \quad (6)$$

Given these parameters equation 2 can be derived through integration. The complete derivation can be found in [28]

Due to the dependence of k_1 and k_3 on P_{LV0}/R_{valve} as shown in equation 3, limit considerations are required. V_{ED0} is the volume at which the pressure in the ventricle is zero in the passive pressure-volume relationship. At this point in the pressure-volume relationship, the ventricle is neither actively contracted nor passively stretched. This state, if at all reached during the cardiac cycle, will occur close to the end of systole if the ventricular volume is completely ejected. End-systolic volumes from MRI were reported to range from 25 ml to 80 ml at 95% confidence for males aged 20-79 yrs. [59]. Given that end-systolic volume will typically be larger than V_{ED0}, it appears safe to assume a lower contraint of 10 ml for this parameter. This means the volume at zero pressure is 10ml or less

Additionally, the maximum and minimum values P_{LV0}/R_{valve} can assume can be determined from k_3. The minimum value is trivial as P_{LV0}/R_{valve} approaching 0 ml/s would require k_1 to approach 0 ml/s, whilst k_3 would be equal to P_{cvp}/R_{valve}. The other extreme would be $k_3 \approx P_{LV0}/R_{valve}$. Taking this upper limit and the derived physiological lower constraint on V_{ED0} into account, valid values of k_1 are given by the inequality Equation 7.

$$k_1 \leq - k_3 e^{10ml * \beta} \quad (7)$$

Applying these constraints allows for the model to be evaluated within physiological limits of the left ventricle in which V_{ED0} >= 0 ml and pressures P_{LV0} and P_{cvp} are positive.

Uncertainty Quantification

The Hessian at a maximum has a negative which is a positive-definite matrix such that the inverse is the covariance. In the case of ill-posed nonlinear problem, the Hessian often becomes non-positive definite, thus non-invertible in the sense of a classical covariance, with the general advice being a rethink of the model. Re-specifying a mechanistically motivated model of the underlying physiology would alter the model to an extent which no longer describes the physiology or, alternatively, becomes so high dimensional that the computational cost of solving becomes prohibitive. Both cases are unsatisfactory. Thus, we employed a routine described by Jeff Gill et al. [45] for the computation of the general

inverse of a non-invertible Hessian as an estimation of the error. This procedure uses the Moore-Penrose generalized inverse followed by a Schnabel-Eskow Cholesky decomposition which produces a positive definite matrix.

Expanded Bibliography:

23. D. D. Glower, J. A. Spratt, N. D. Snow et al., “Linearity of the Frank–Starling relationship in the intact heart: the concept of preload recruitable stroke work,” Circulation, vol. 71, no. 5, pp. 994–1009, 1985.

28. S. Zenker, J. Rubin, and G. Clermont, “From inverse problems in mathematical physiology to quantitative differential diagnoses,” PLoS Computational Biology, vol. 3, no. 11, Article ID e204, 2007.

45. J. Gill and G. King, “What to do when your hessian is not invertible,” Sociological Methods & Research, vol. 33, no. 1, pp. 54–87, 2004.

59. A. Maceira, S. Prasad, M. Khan, and D. Pennell, “Normalized left ventricular systolic and diastolic function by steady state free precession cardiovascular magnetic resonance,” Journal of Cardiovascular Magnetic Resonance, vol. 8, no. 3, pp. 417–426, 2006.

Expanded Results

The following section contains the plots corresponding to figures 1 and 2 of the Results section of the manuscript for all patients. First, we will show the plots of the AF patients structured in fits and residuals against filling interval, prior RR interval and observed Pulse Pressure for the simple and expanded model. The fits and residuals associated with the simple model are displayed on the left of each panel and those of the expanded model on the right hand side. The results for the sinus patients come afterwards in the same order.

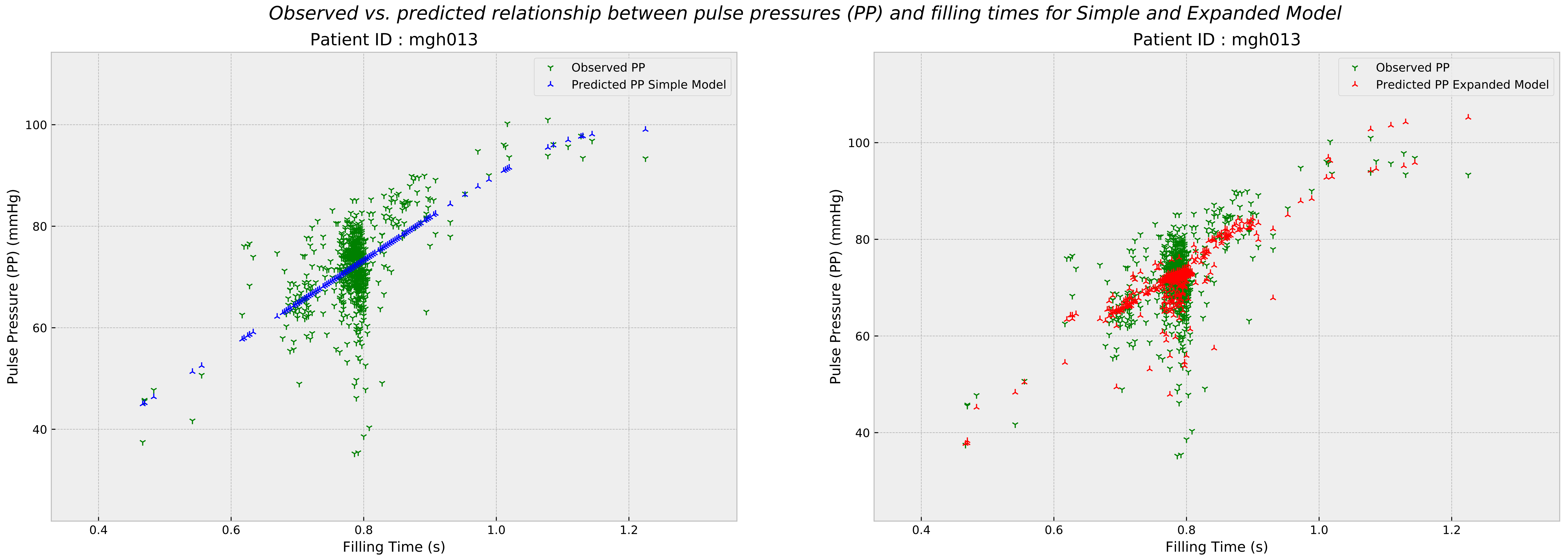

Observed vs. predicted relationship between pulse pressures (PP) and filling times for Simple and Expanded Model
Patient ID : mgh013
Observed PP
Predicted PP Simple Model
Pulse Pressure (PP) (mmHg)
Filling Time (s)
Patient ID : mgh013
Observed PP
Predicted PP Expanded Model
Pulse Pressure (PP) (mmHg)
Filling Time (s)

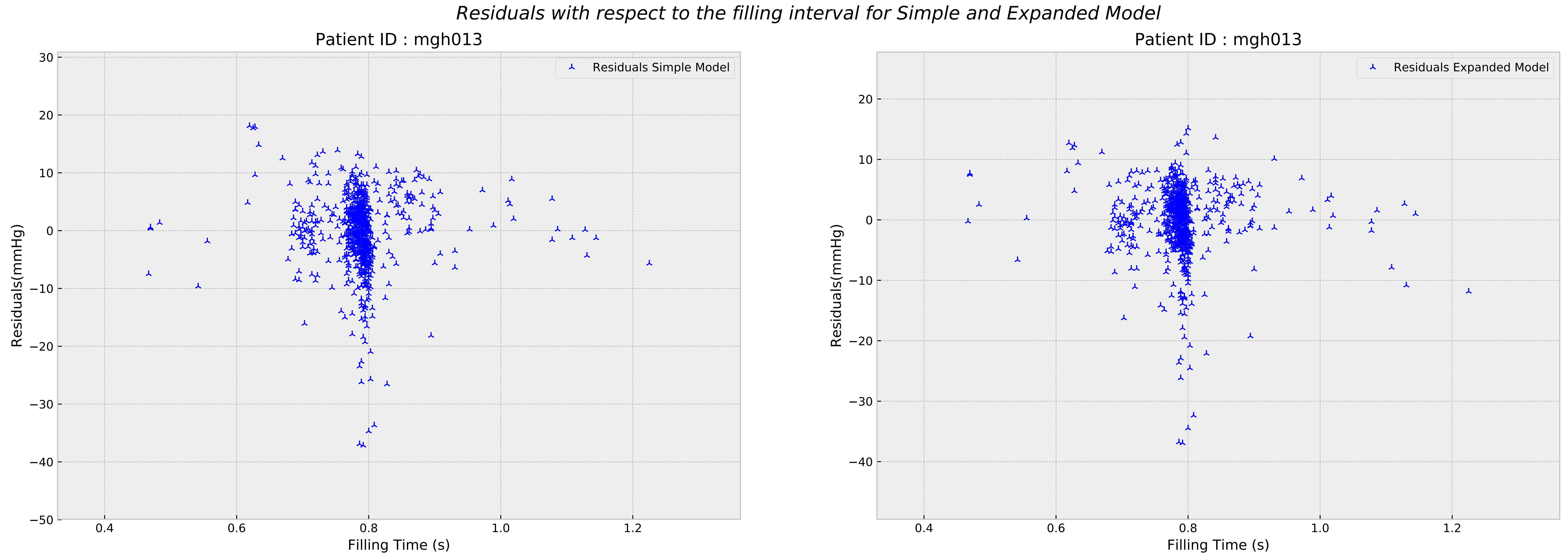
Residuals with respect to the filling interval for Simple and Expanded Model
Patient ID : mgh013
Residuals Simple Model
Residuals(mmHg)
Filling Time (s)
30
20
10
0
−10
−20
−30
−40
−50
0.4
0.6
0.8
1.0
1.2
Patient ID : mgh013
Residuals Expanded Model
Residuals(mmHg)
Filling Time (s)
20
10
0
−10
−20
−30
−40
0.4
0.6
0.8
1.0
1.2

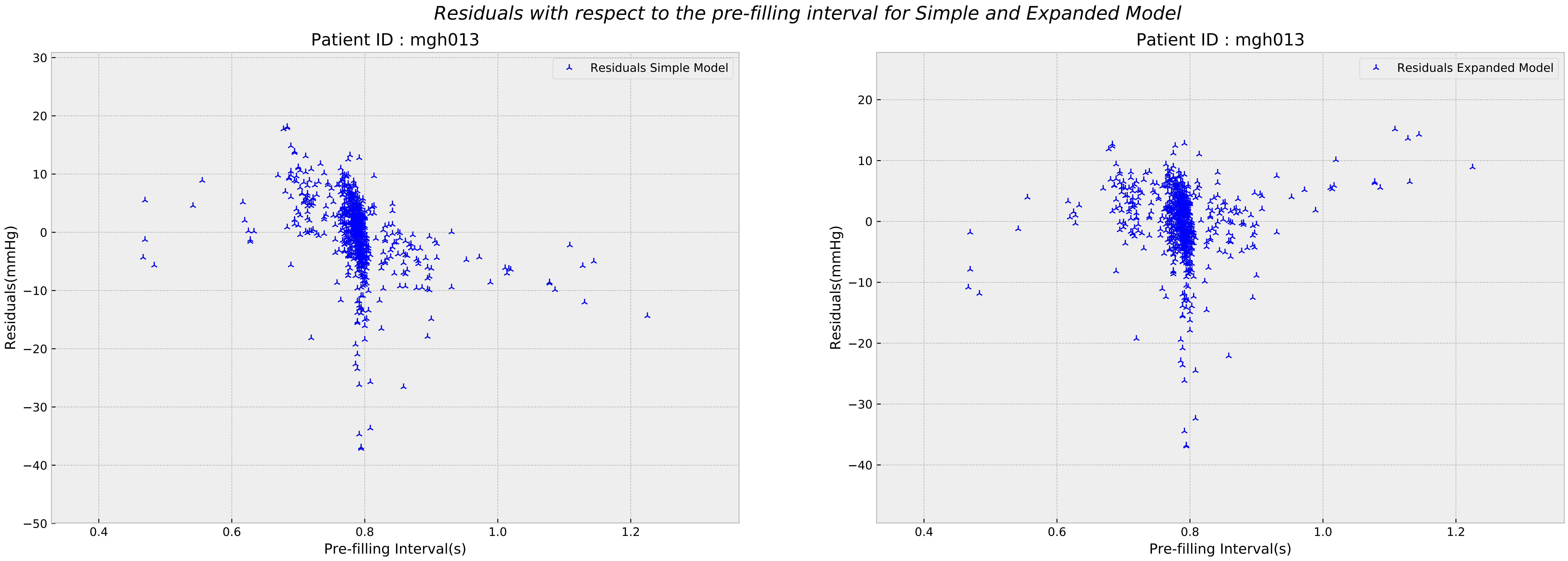
Residuals with respect to the pre-filling interval for Simple and Expanded Model
Patient ID : mgh013
Residuals Simple Model
Residuals(mmHg)
Pre-filling Interval(s)
Patient ID : mgh013
Residuals Expanded Model
Residuals(mmHg)
Pre-filling Interval(s)

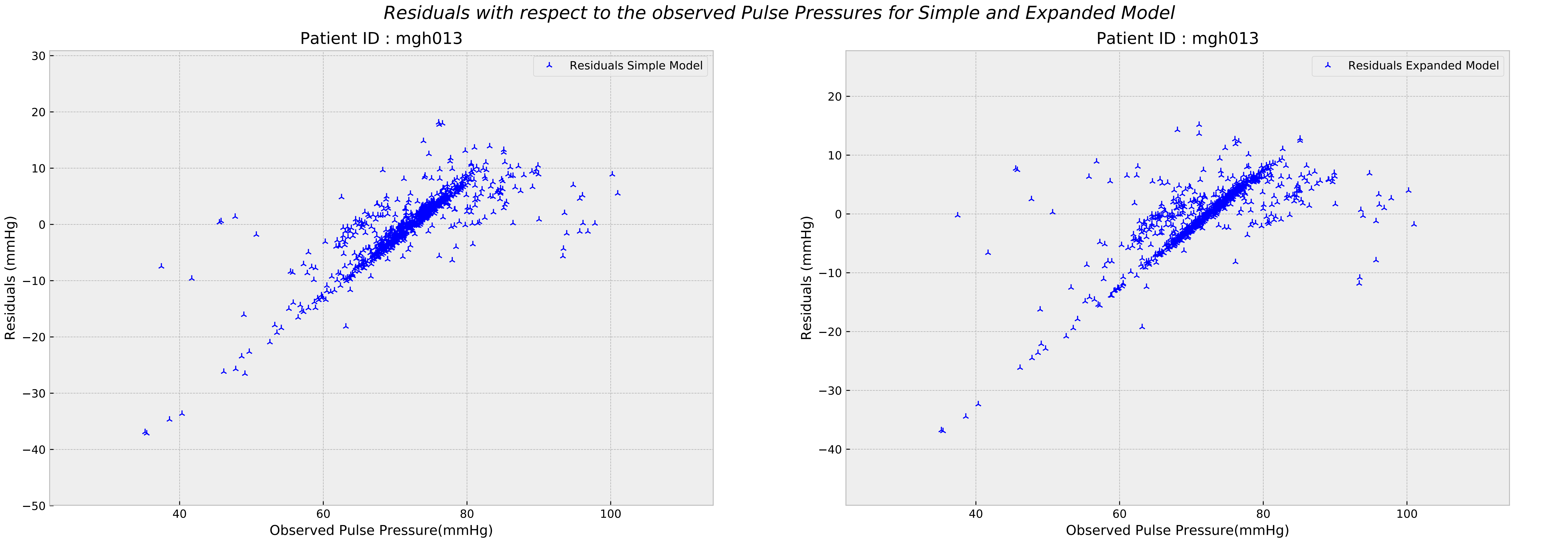
Residuals with respect to the observed Pulse Pressures for Simple and Expanded Model
Patient ID : mgh013
Residuals Simple Model
Residuals (mmHg)
Observed Pulse Pressure(mmHg)
30
20
10
0
−10
−20
−30
−40
−50
40
60
80
100
Patient ID : mgh013
Residuals Expanded Model
Residuals (mmHg)
Observed Pulse Pressure(mmHg)
20
10
0
−10
−20
−30
−40
40
60
80
100

Observed vs. predicted relationship between pulse pressures (PP) and filling times for Simple and Expanded Model

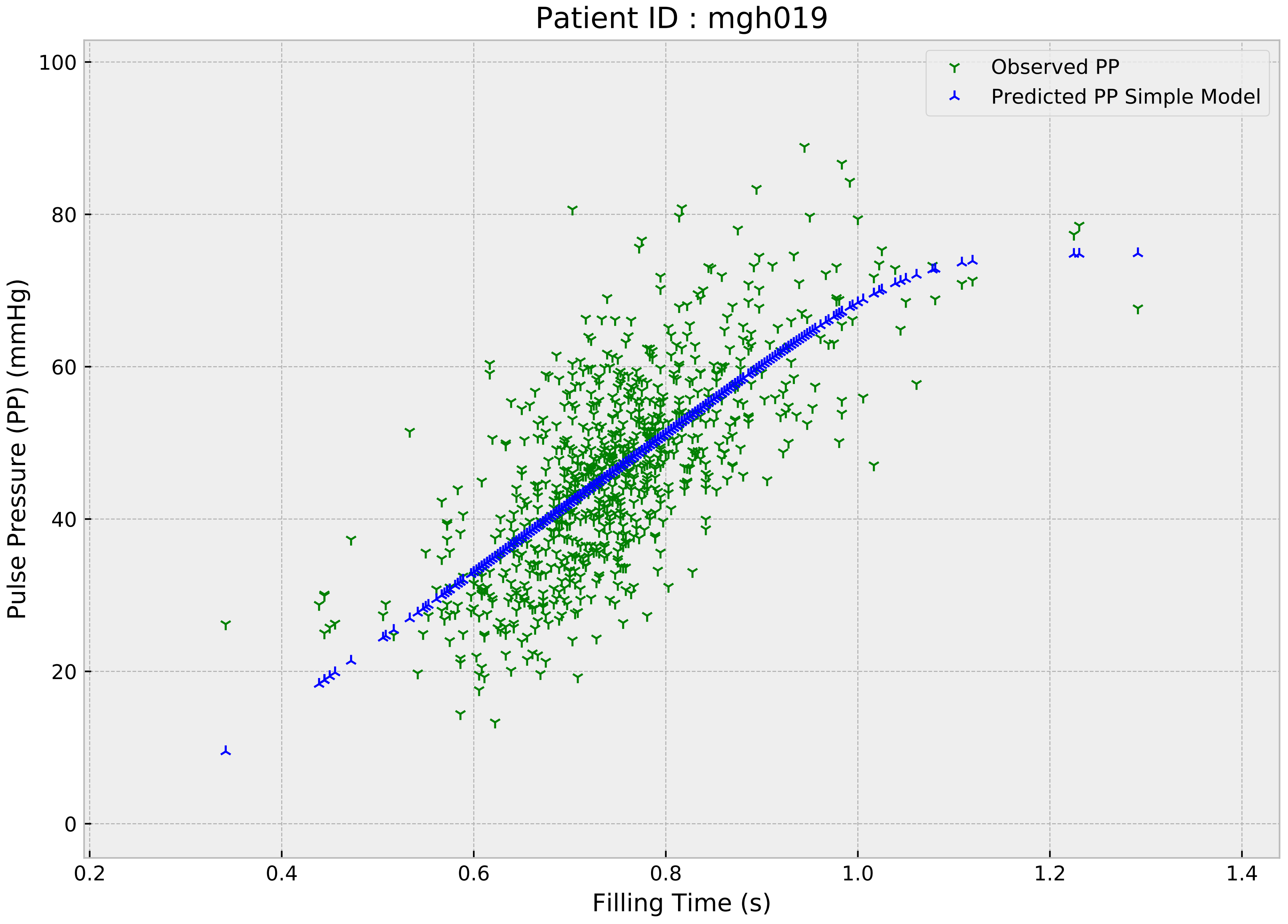

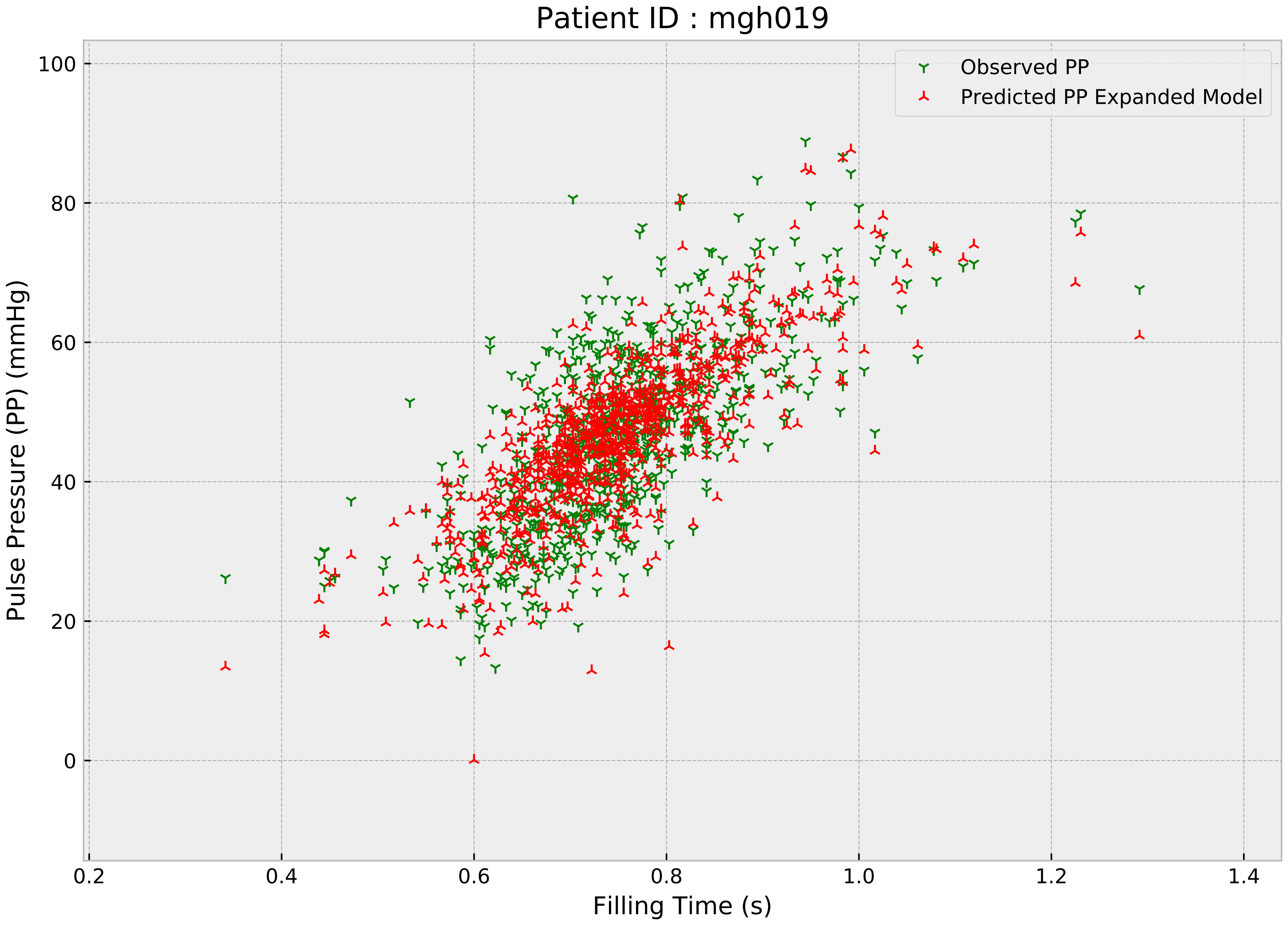

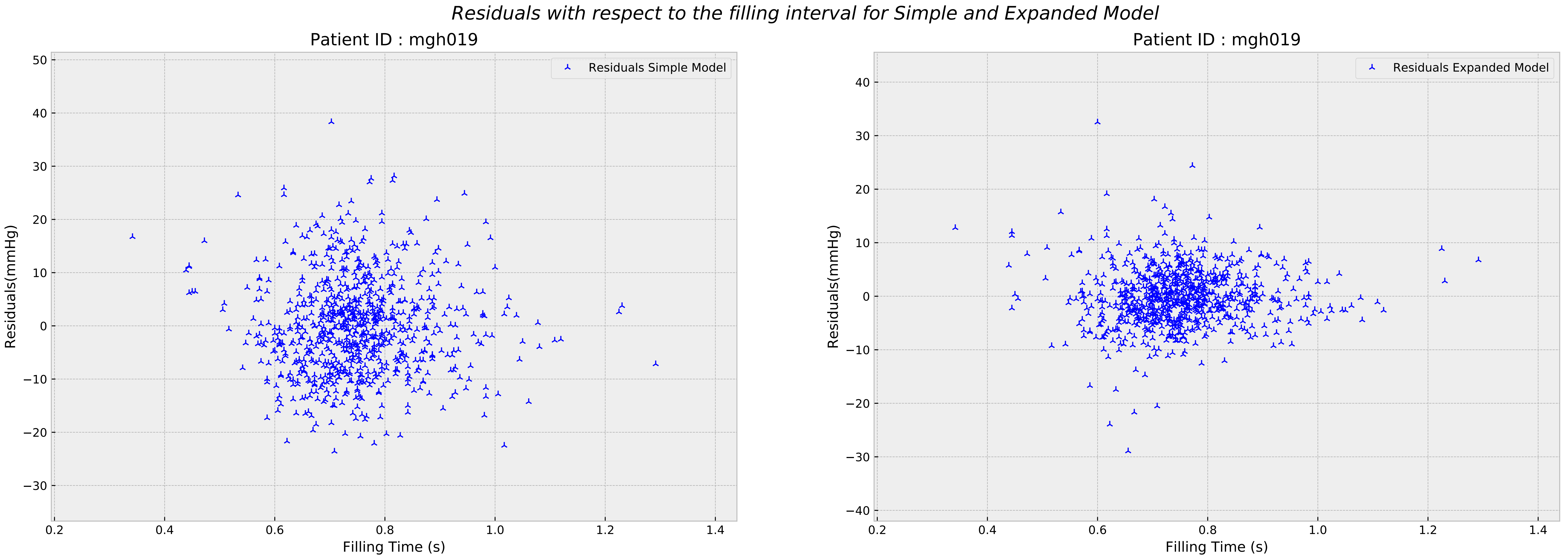
Residuals with respect to the filling interval for Simple and Expanded Model
Patient ID : mgh019
Residuals Simple Model
Residuals(mmHg)
Filling Time (s)
Patient ID : mgh019
Residuals Expanded Model
Residuals(mmHg)
Filling Time (s)

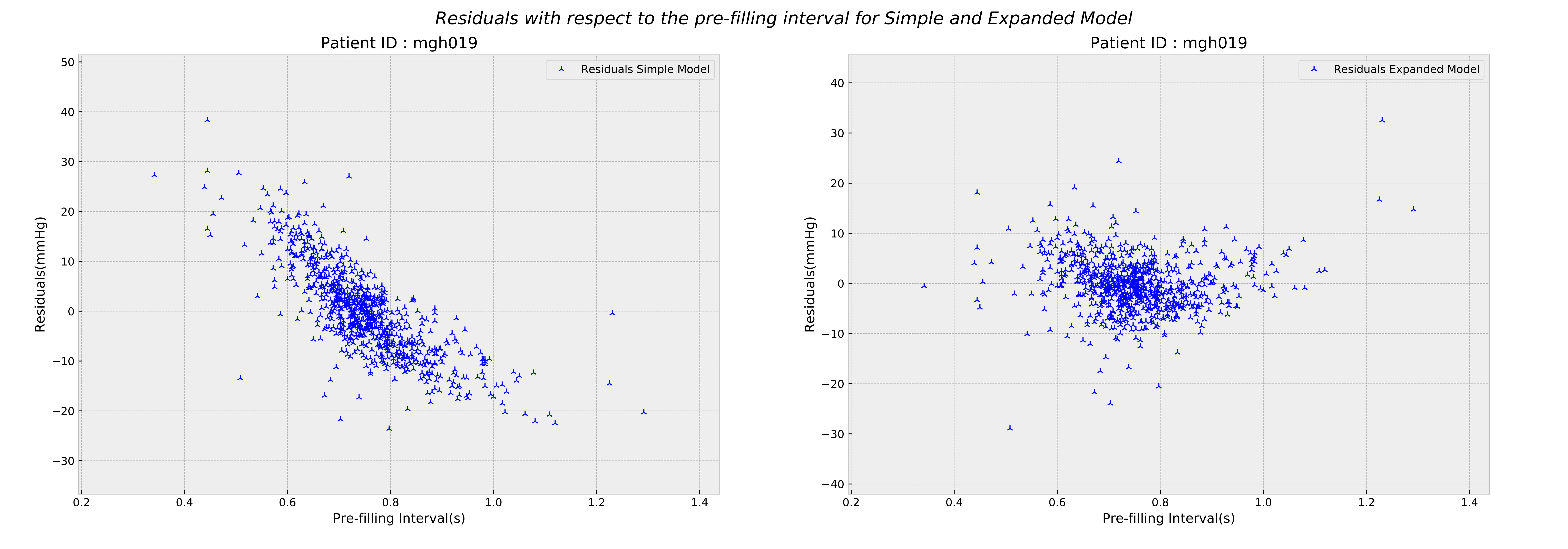
Residuals with respect to the pre-filling interval for Simple and Expanded Model
Patient ID : mgh019
Residuals Simple Model
Residuals(mmHg)
Pre-filling Interval(s)
Patient ID : mgh019
Residuals Expanded Model
Residuals(mmHg)
Pre-filling Interval(s)

Residuals with respect to the observed Pulse Pressures for Simple and Expanded Model

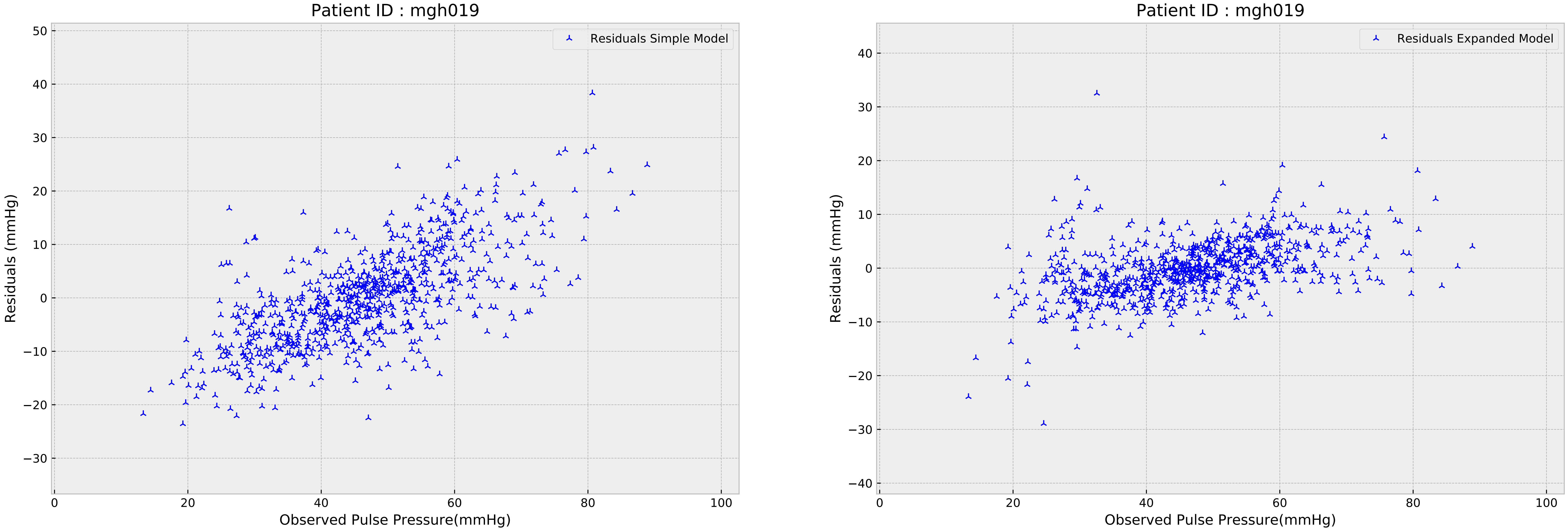

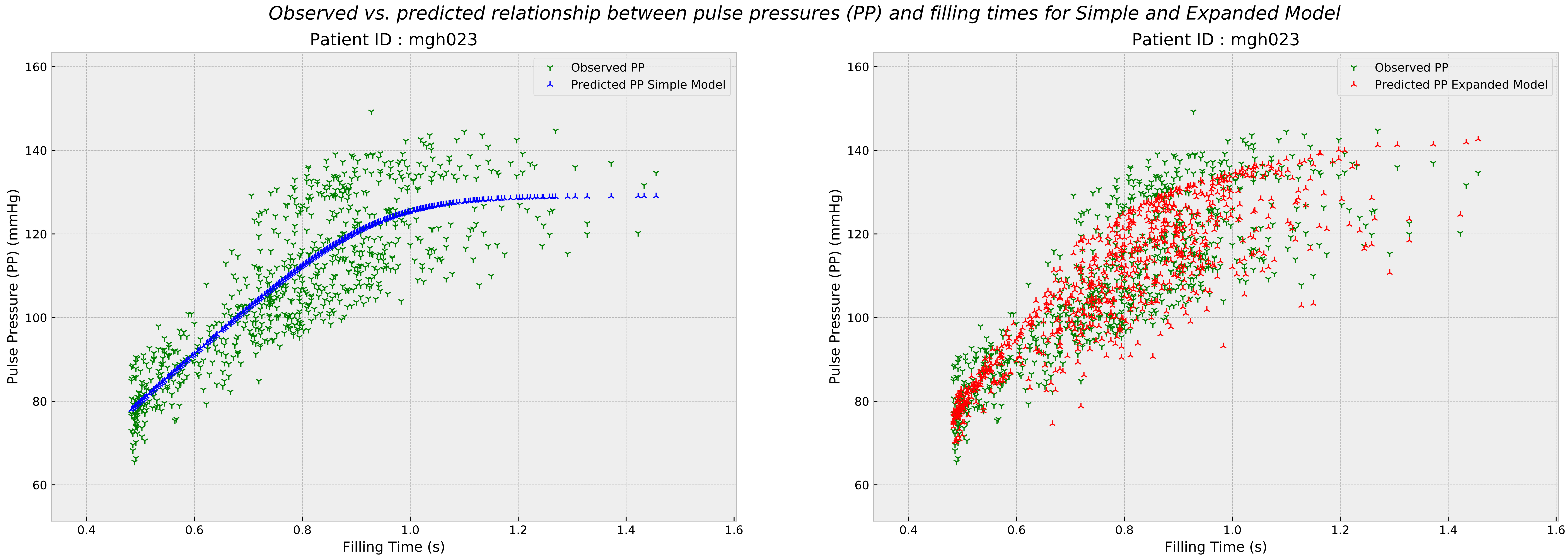

Observed vs. predicted relationship between pulse pressures (PP) and filling times for Simple and Expanded Model
Patient ID : mgh023
Observed PP
Predicted PP Simple Model
Pulse Pressure (PP) (mmHg)
Filling Time (s)
160
140
120
100
80
60
0.4
0.6
0.8
1.0
1.2
1.4
1.6
Patient ID : mgh023
Observed PP
Predicted PP Expanded Model
Pulse Pressure (PP) (mmHg)
Filling Time (s)

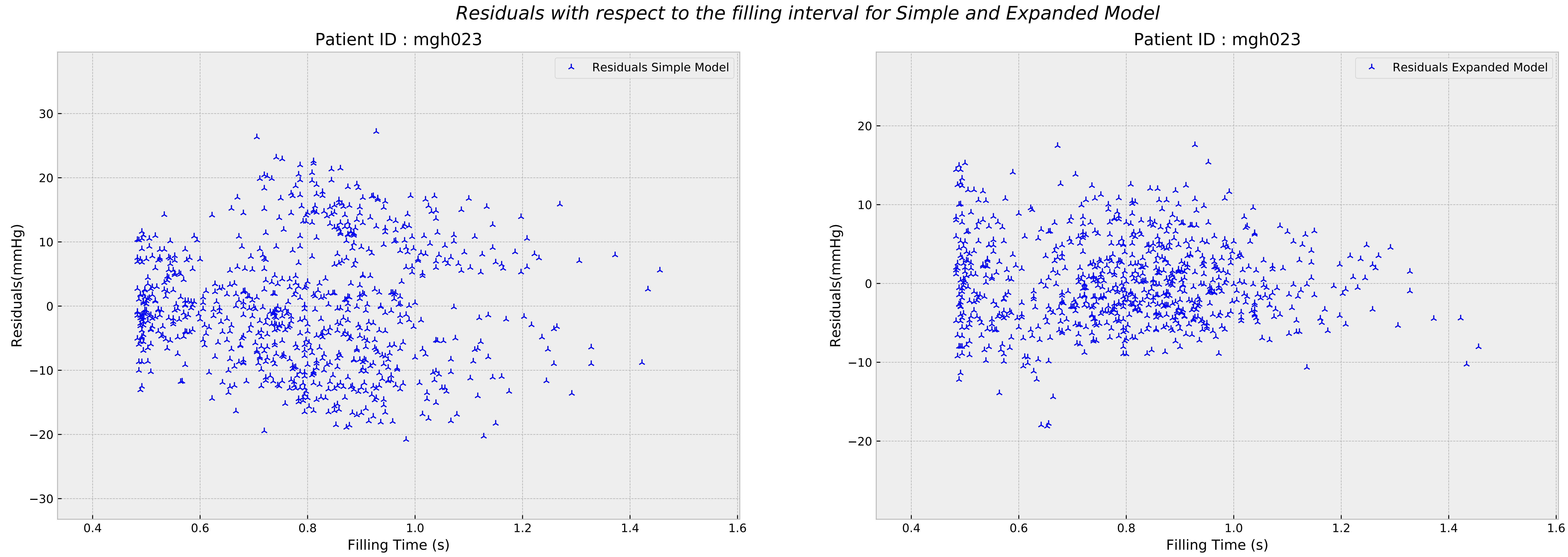

Residuals with respect to the filling interval for Simple and Expanded Model
Patient ID : mgh023
Residuals Simple Model
Residuals(mmHg)
Filling Time (s)
Patient ID : mgh023
Residuals Expanded Model
Residuals(mmHg)
Filling Time (s)

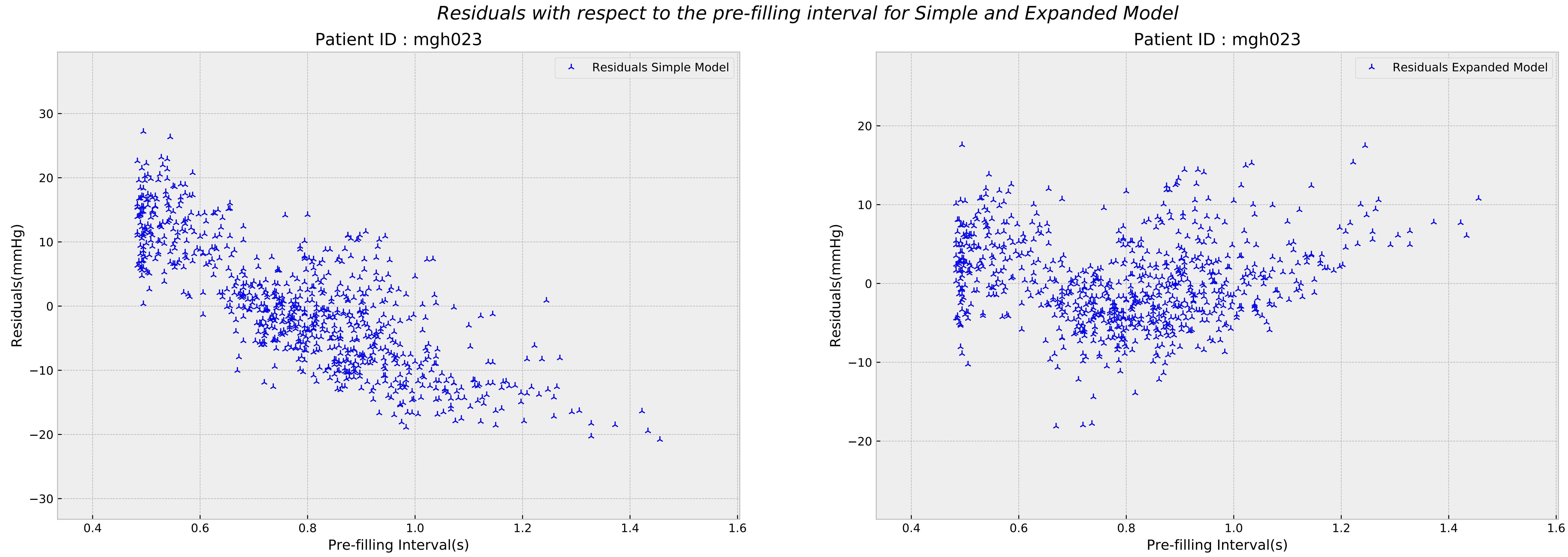
Residuals with respect to the pre-filling interval for Simple and Expanded Model
Patient ID : mgh023
Residuals Simple Model
Residuals(mmHg)
Pre-filling Interval(s)
Patient ID : mgh023
Residuals Expanded Model
Residuals(mmHg)
Pre-filling Interval(s)

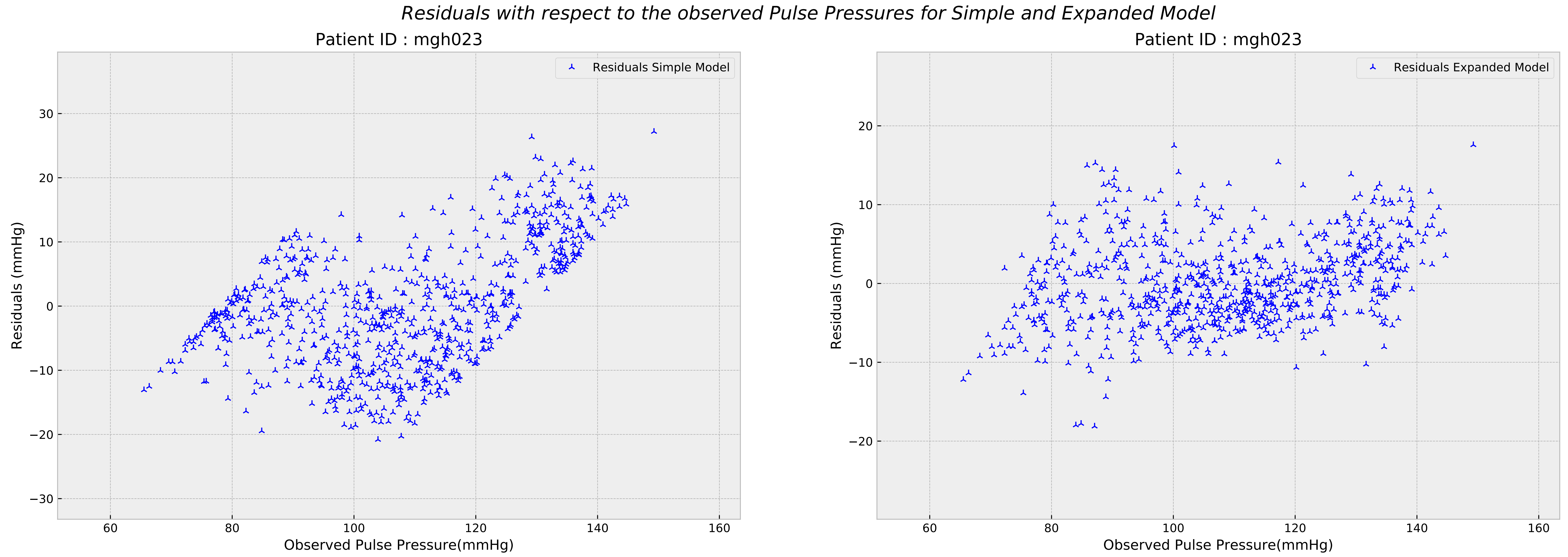
Residuals with respect to the observed Pulse Pressures for Simple and Expanded Model
Patient ID : mgh023
Residuals Simple Model
Residuals (mmHg)
Observed Pulse Pressure(mmHg)
30
20
10
0
−10
−20
−30
60
80
100
120
140
160
Patient ID : mgh023
Residuals Expanded Model
Residuals (mmHg)
Observed Pulse Pressure(mmHg)
20
10
0
−10
−20
60
80
100
120
140
160

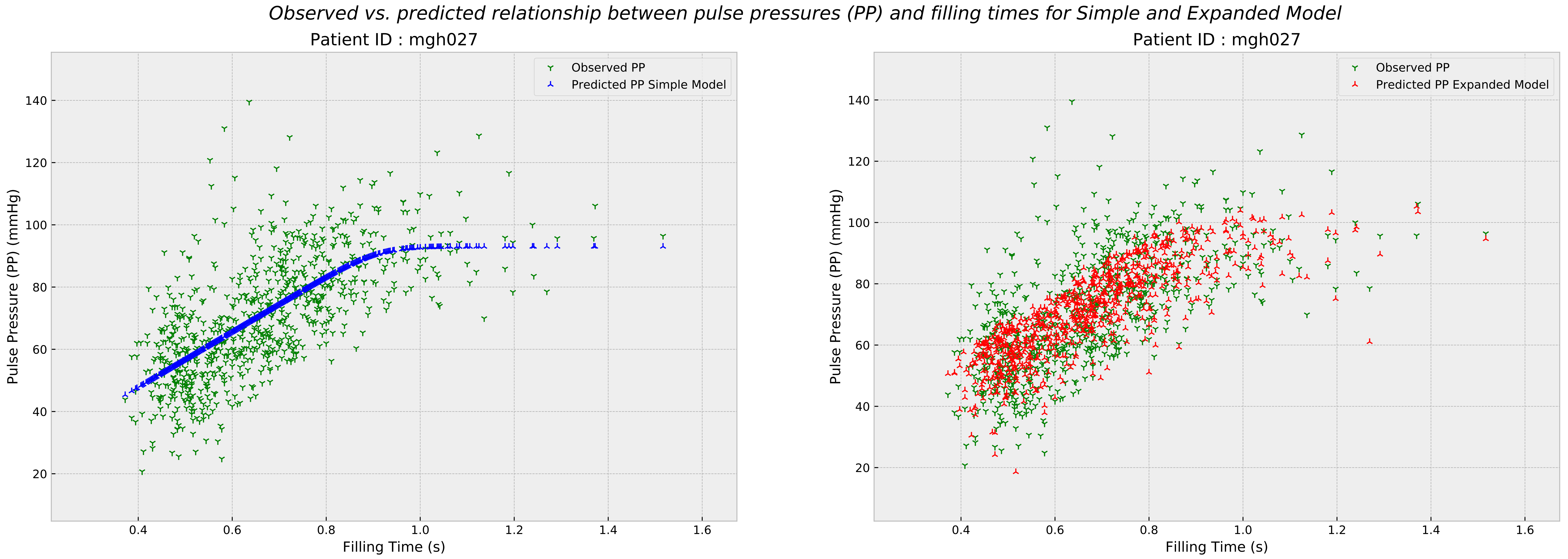
Observed vs. predicted relationship between pulse pressures (PP) and filling times for Simple and Expanded Model
Patient ID : mgh027
Observed PP
Predicted PP Simple Model
Pulse Pressure (PP) (mmHg)
Filling Time (s)
20
40
60
80
100
120
140
0.4
0.6
0.8
1.0
1.2
1.4
1.6
Patient ID : mgh027
Observed PP
Predicted PP Expanded Model
Pulse Pressure (PP) (mmHg)
Filling Time (s)

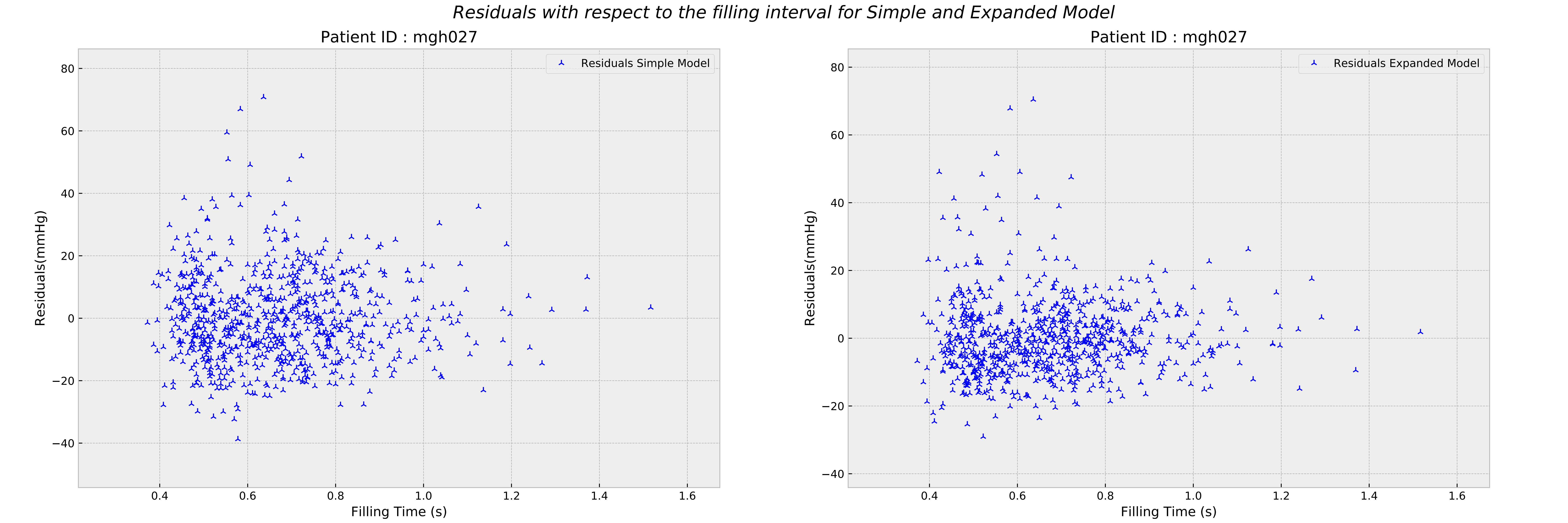
Residuals with respect to the filling interval for Simple and Expanded Model
Patient ID : mgh027
Residuals Simple Model
Residuals(mmHg)
Filling Time (s)
Patient ID : mgh027
Residuals Expanded Model
Residuals(mmHg)
Filling Time (s)

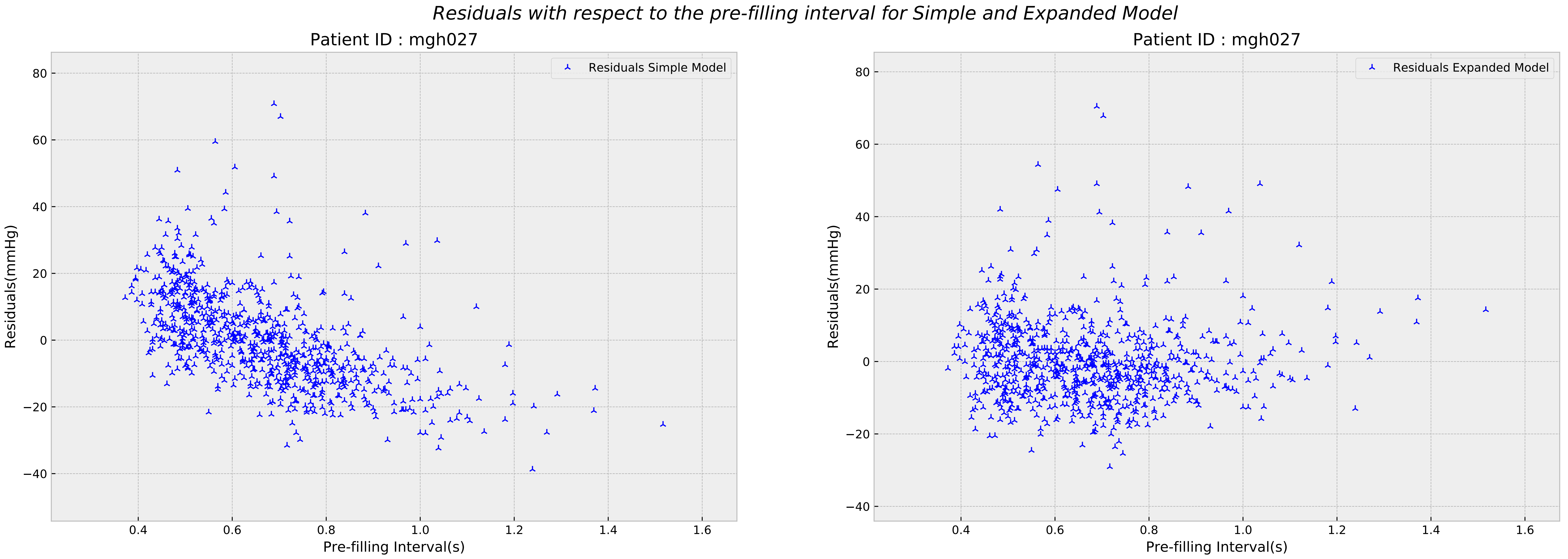
Residuals with respect to the pre-filling interval for Simple and Expanded Model
Patient ID : mgh027
Residuals Simple Model
Residuals(mmHg)
Pre-filling Interval(s)
80
60
40
20
0
−20
−40
0.4
0.6
0.8
1.0
1.2
1.4
1.6
Patient ID : mgh027
Residuals Expanded Model
Residuals(mmHg)
Pre-filling Interval(s)

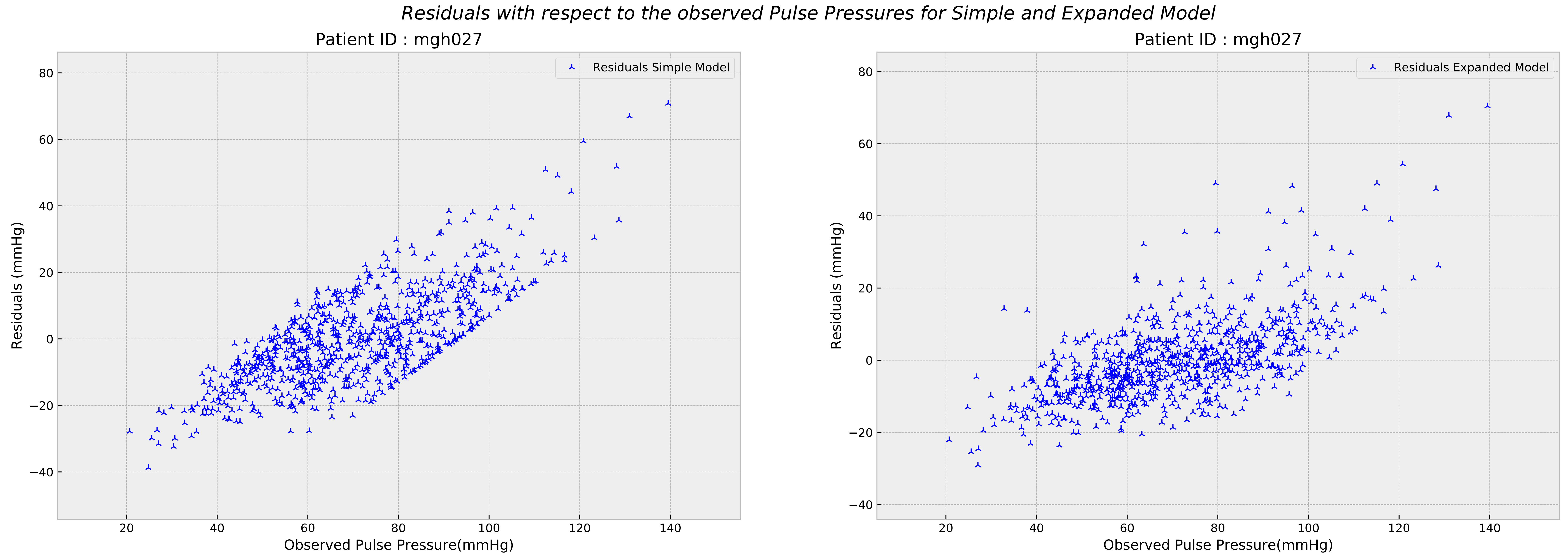
Residuals with respect to the observed Pulse Pressures for Simple and Expanded Model
Patient ID : mgh027
Residuals Simple Model
Residuals (mmHg)
Observed Pulse Pressure(mmHg)
80
60
40
20
0
−20
−40
20
40
60
80
100
120
140
Patient ID : mgh027
Residuals Expanded Model
Residuals (mmHg)
Observed Pulse Pressure(mmHg)
80
60
40
20
0
−20
−40
20
40
60
80
100
120
140

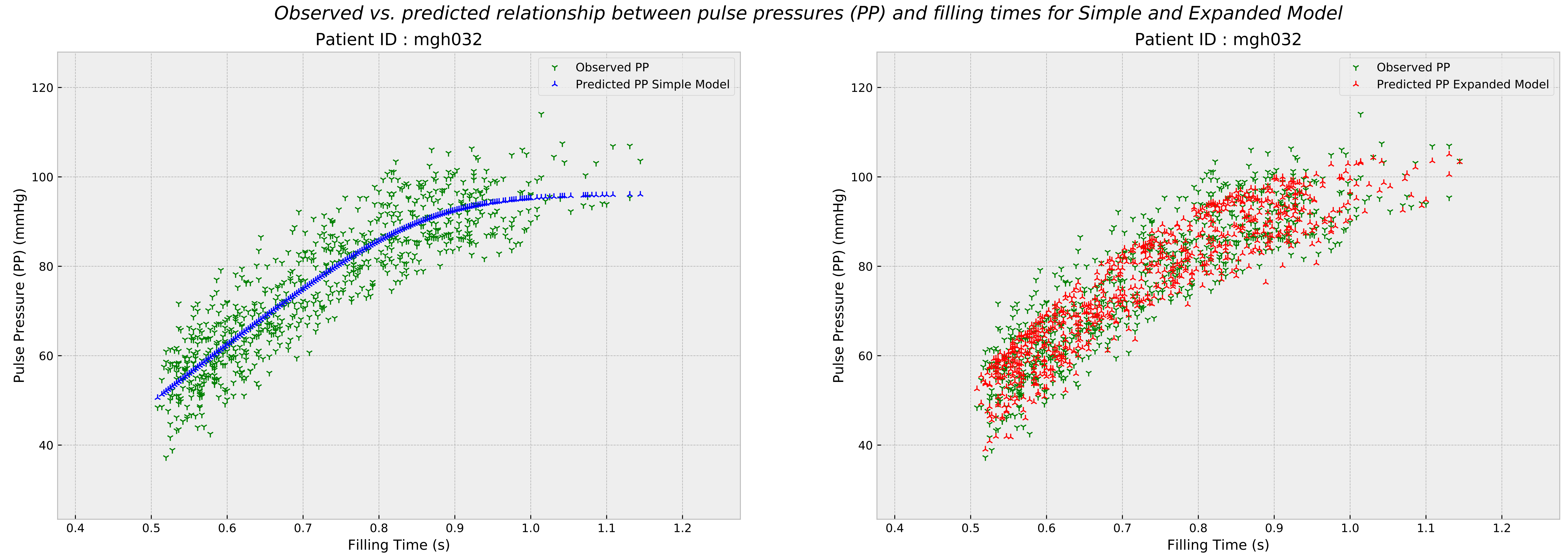

Observed vs. predicted relationship between pulse pressures (PP) and filling times for Simple and Expanded Model
Patient ID : mgh032
Observed PP
Predicted PP Simple Model
Pulse Pressure (PP) (mmHg)
Filling Time (s)
120
100
80
60
40
0.4
0.5
0.6
0.7
0.8
0.9
1.0
1.1
1.2
Patient ID : mgh032
Observed PP
Predicted PP Expanded Model
Pulse Pressure (PP) (mmHg)
Filling Time (s)

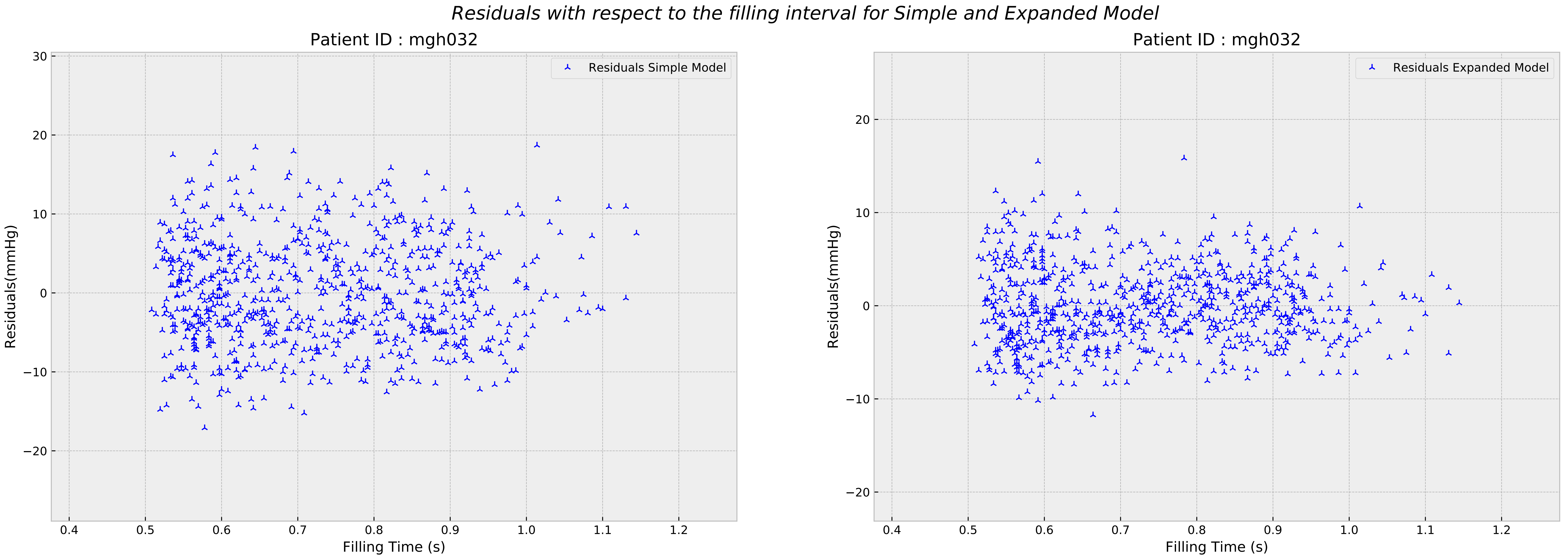
Residuals with respect to the filling interval for Simple and Expanded Model
Patient ID : mgh032
Residuals Simple Model
Residuals(mmHg)
Filling Time (s)
30
20
10
0
−10
−20
0.4
0.5
0.6
0.7
0.8
0.9
1.0
1.1
1.2
Patient ID : mgh032
Residuals Expanded Model
Residuals(mmHg)
Filling Time (s)
20
10
0
−10
−20
0.4
0.5
0.6
0.7
0.8
0.9
1.0
1.1
1.2

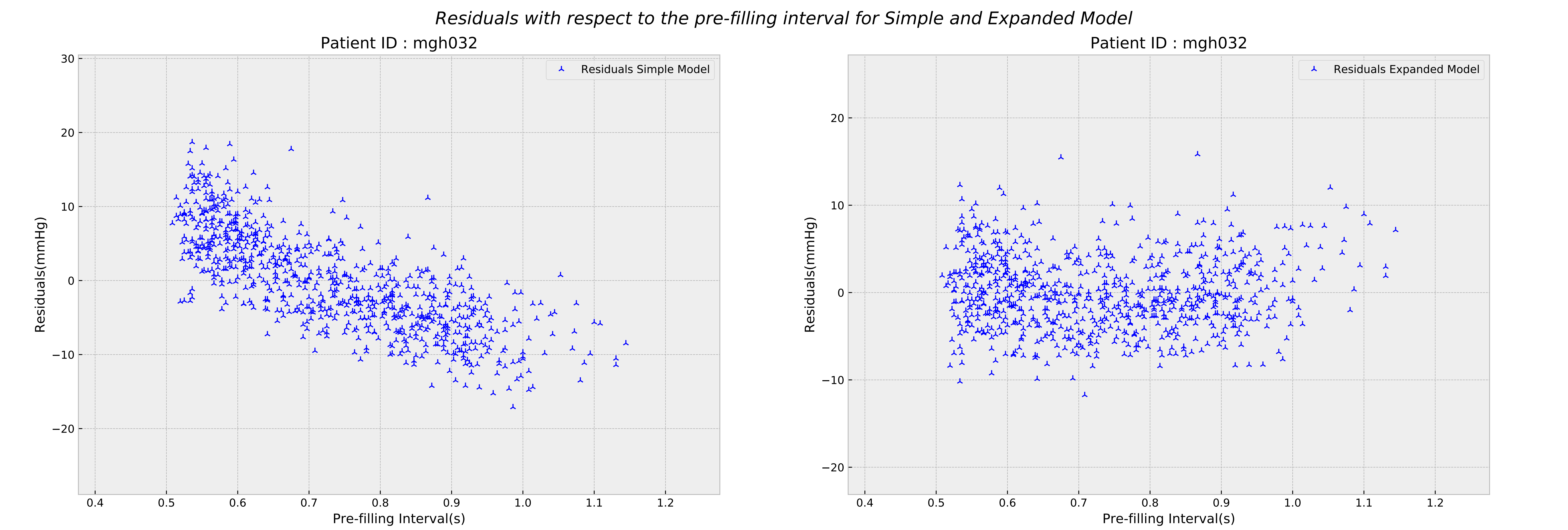
Residuals with respect to the pre-filling interval for Simple and Expanded Model
Patient ID : mgh032
Residuals Simple Model
Residuals(mmHg)
Pre-filling Interval(s)
Patient ID : mgh032
Residuals Expanded Model
Residuals(mmHg)
Pre-filling Interval(s)

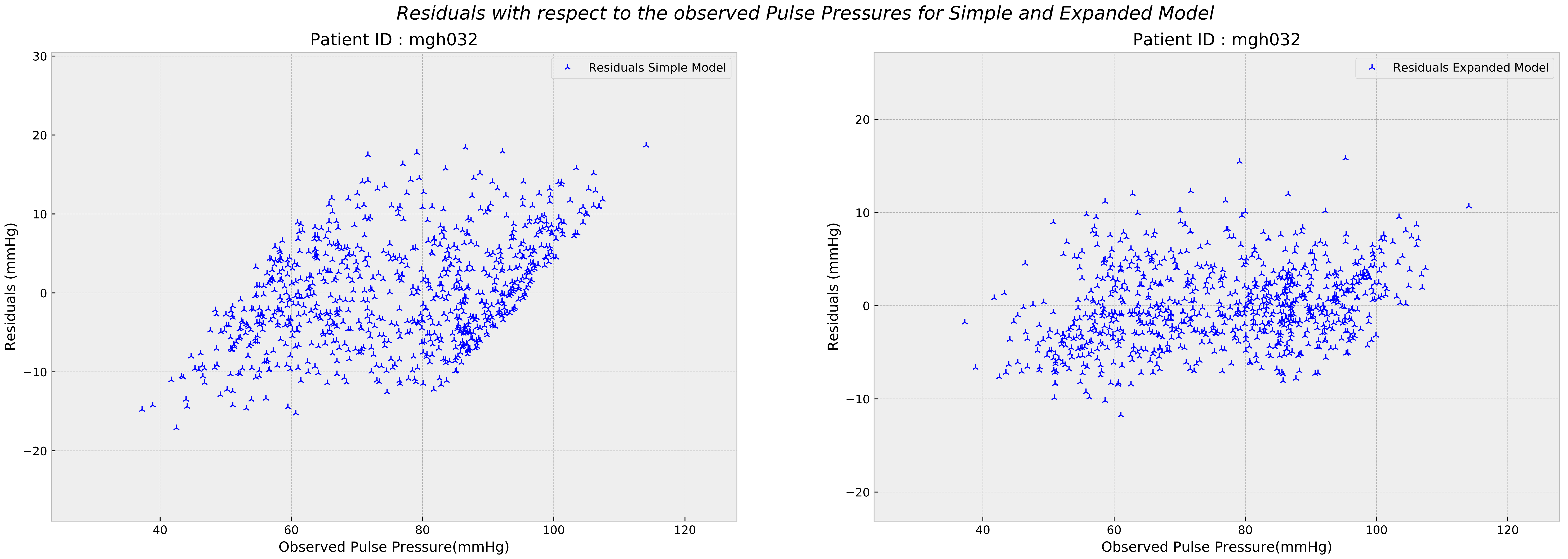
Residuals with respect to the observed Pulse Pressures for Simple and Expanded Model
Patient ID : mgh032
Residuals Simple Model
Residuals (mmHg)
30
20
10
0
−10
−20
40
60
80
100
120
Observed Pulse Pressure(mmHg)
Patient ID : mgh032
Residuals Expanded Model
Residuals (mmHg)
20
10
0
−10
−20
40
60
80
100
120
Observed Pulse Pressure(mmHg)

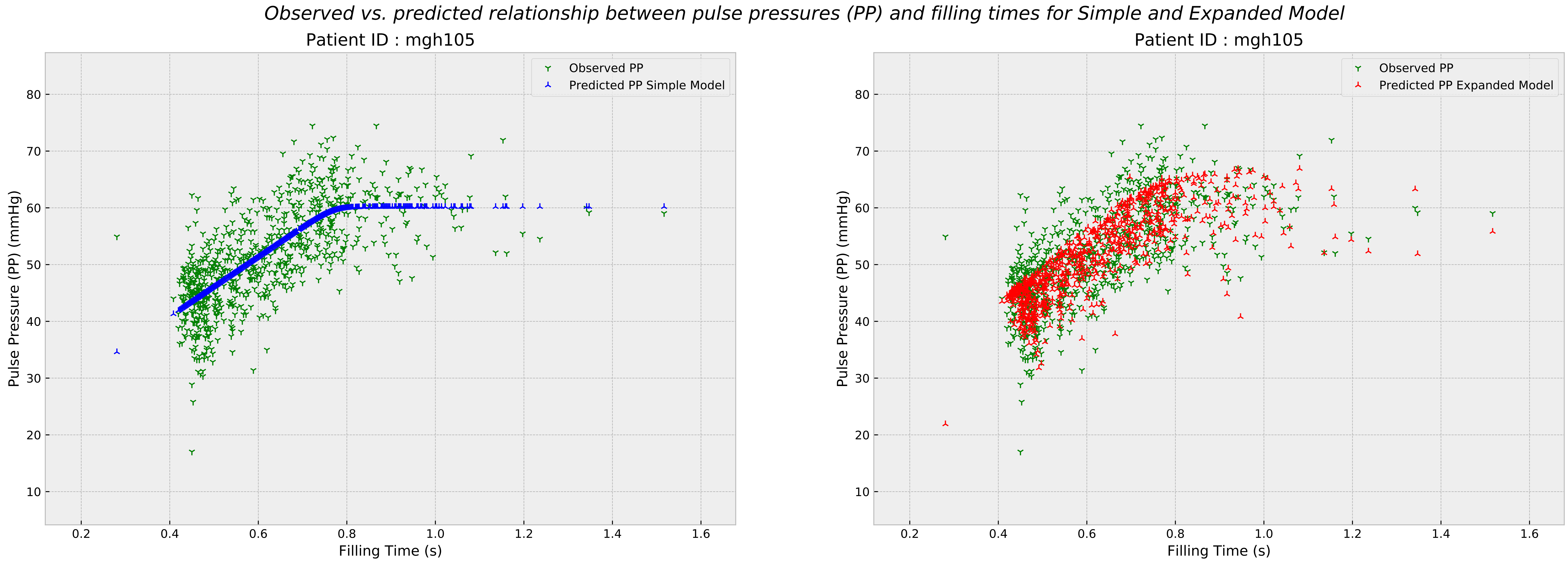

Observed vs. predicted relationship between pulse pressures (PP) and filling times for Simple and Expanded Model
Patient ID : mgh105
Observed PP
Predicted PP Simple Model
Pulse Pressure (PP) (mmHg)
Filling Time (s)
10
20
30
40
50
60
70
80
0.2
0.4
0.6
0.8
1.0
1.2
1.4
1.6
Patient ID : mgh105
Observed PP
Predicted PP Expanded Model
Pulse Pressure (PP) (mmHg)
Filling Time (s)

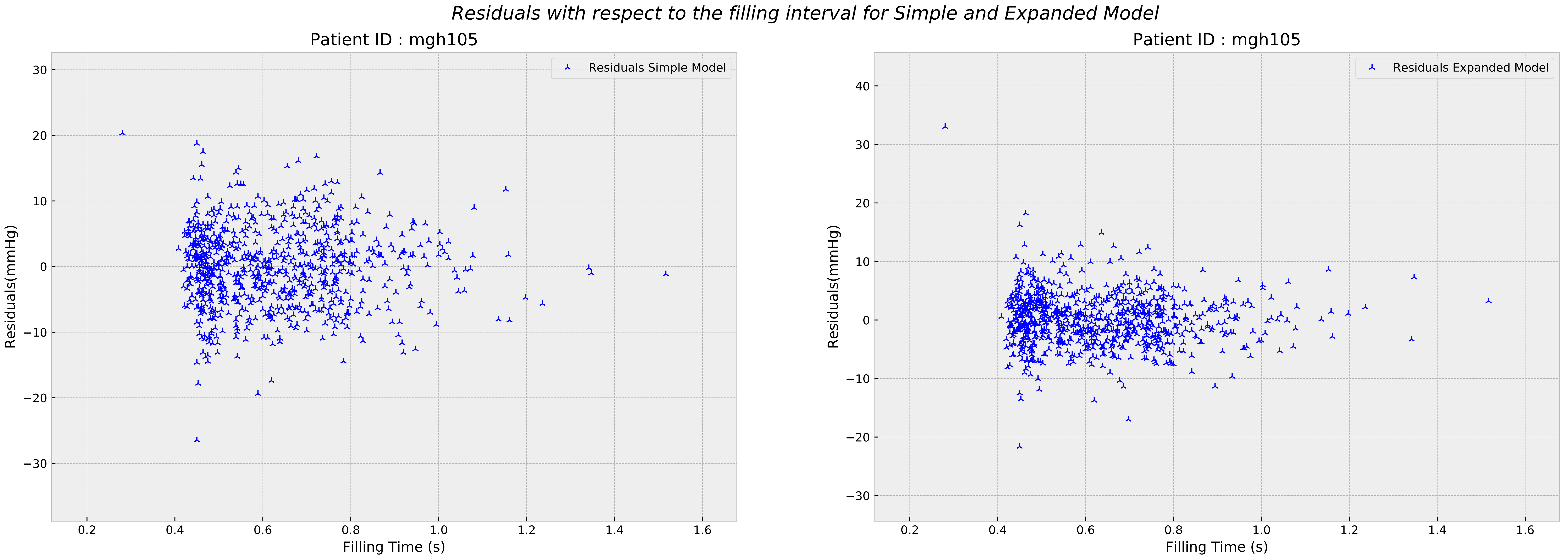
Residuals with respect to the filling interval for Simple and Expanded Model
Patient ID : mgh105
Residuals Simple Model
Residuals(mmHg)
30
20
10
0
−10
−20
−30
0.2
0.4
0.6
0.8
1.0
1.2
1.4
1.6
Filling Time (s)
Patient ID : mgh105
Residuals Expanded Model
Residuals(mmHg)
40
30
20
10
0
−10
−20
−30
0.2
0.4
0.6
0.8
1.0
1.2
1.4
1.6
Filling Time (s)

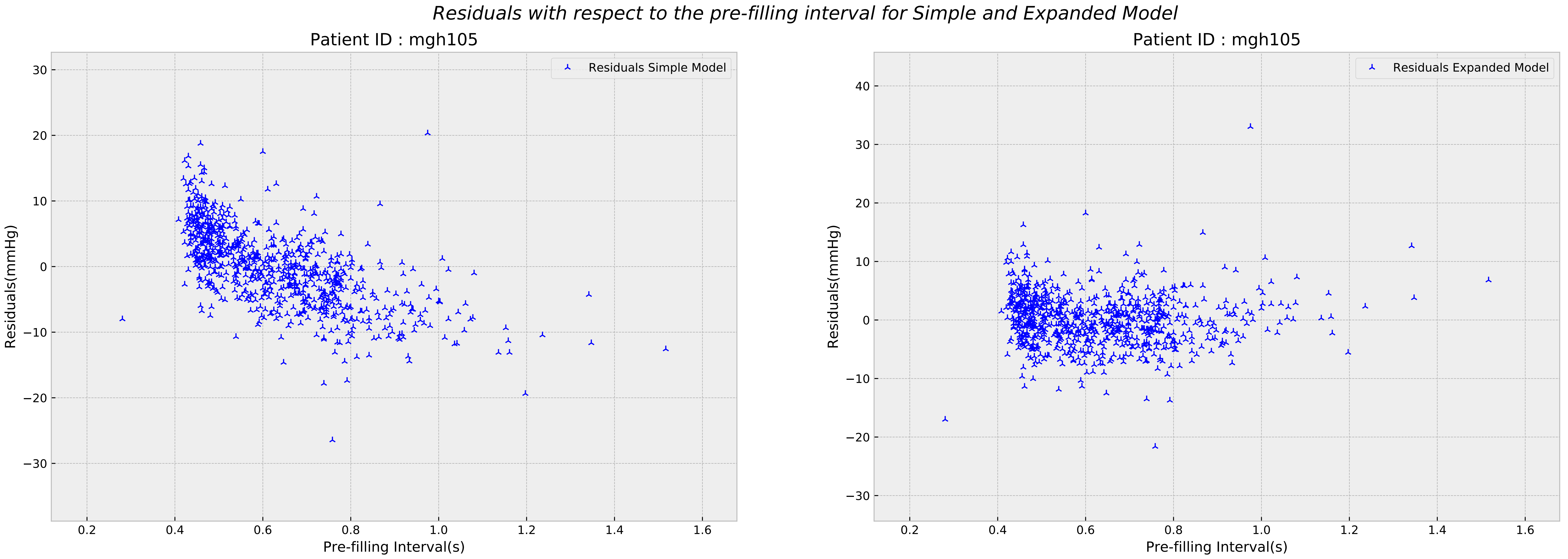

Residuals with respect to the pre-filling interval for Simple and Expanded Model
Patient ID : mgh105
Residuals Simple Model
Residuals(mmHg)
30
20
10
0
−10
−20
−30
0.2
0.4
0.6
0.8
1.0
1.2
1.4
1.6
Pre-filling Interval(s)
Patient ID : mgh105
Residuals Expanded Model
Residuals(mmHg)
40
30
20
10
0
−10
−20
−30
0.2
0.4
0.6
0.8
1.0
1.2
1.4
1.6
Pre-filling Interval(s)

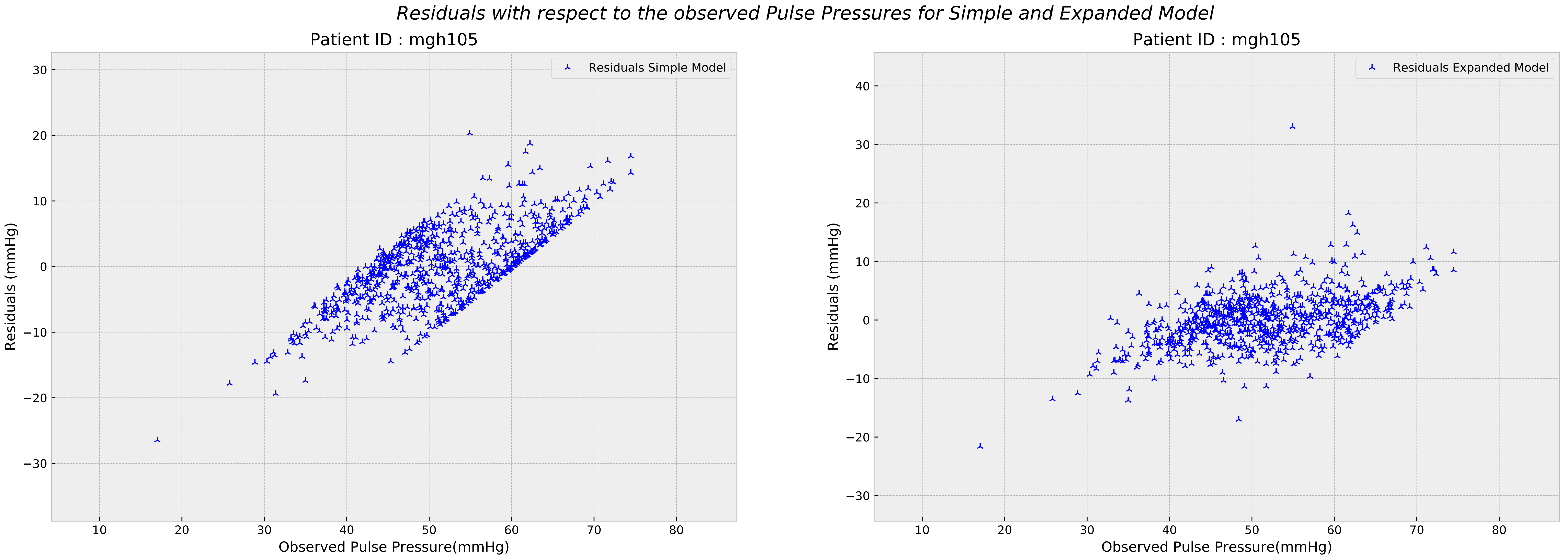

Residuals with respect to the observed Pulse Pressures for Simple and Expanded Model
Patient ID : mgh105
Residuals Simple Model
Residuals (mmHg)
Observed Pulse Pressure(mmHg)
30
20
10
0
−10
−20
−30
10
20
30
40
50
60
70
80
Patient ID : mgh105
Residuals Expanded Model
Residuals (mmHg)
Observed Pulse Pressure(mmHg)
40
30
20
10
0
−10
−20
−30
10
20
30
40
50
60
70
80

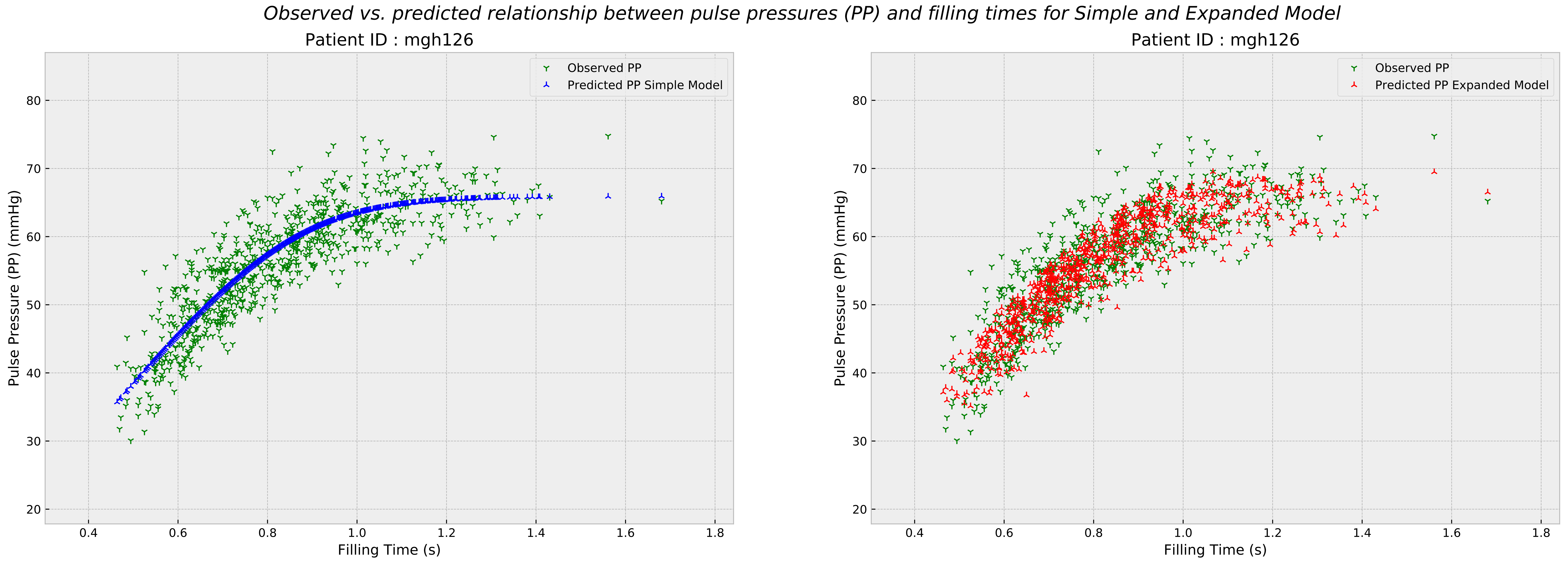
Observed vs. predicted relationship between pulse pressures (PP) and filling times for Simple and Expanded Model
Patient ID : mgh126
Observed PP
Predicted PP Simple Model
Pulse Pressure (PP) (mmHg)
Filling Time (s)
20
30
40
50
60
70
80
0.4
0.6
0.8
1.0
1.2
1.4
1.6
1.8
Patient ID : mgh126
Observed PP
Predicted PP Expanded Model
Pulse Pressure (PP) (mmHg)
Filling Time (s)

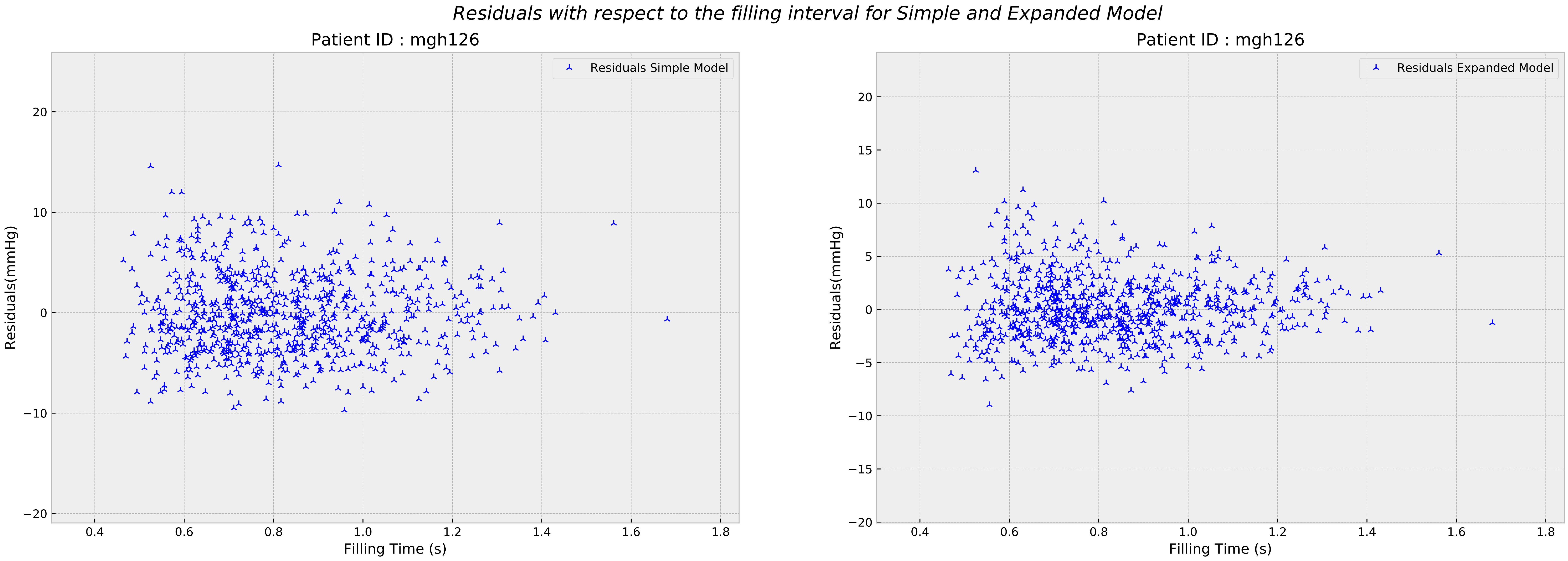
Residuals with respect to the filling interval for Simple and Expanded Model
Patient ID : mgh126
Residuals Simple Model
Residuals(mmHg)
Filling Time (s)
20
10
0
−10
−20
0.4
0.6
0.8
1.0
1.2
1.4
1.6
1.8
Patient ID : mgh126
Residuals Expanded Model
Residuals(mmHg)
Filling Time (s)
20
15
10
5
0
−5
−10
−15
−20
0.4
0.6
0.8
1.0
1.2
1.4
1.6
1.8

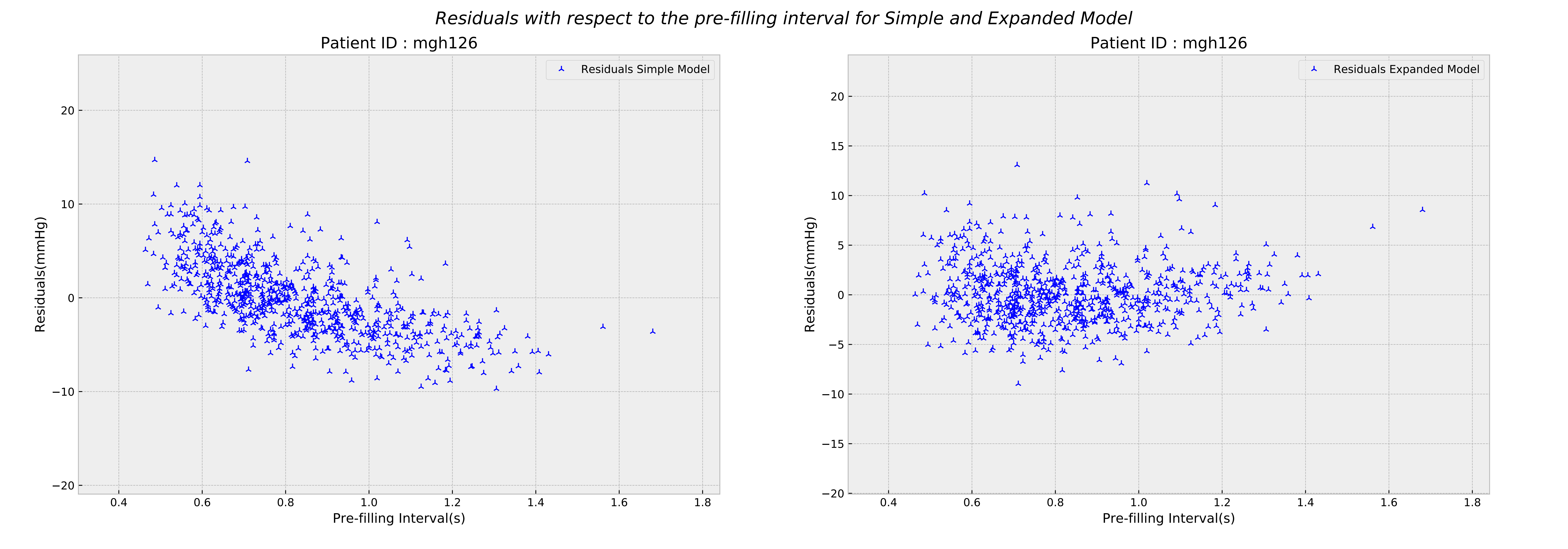

Residuals with respect to the pre-filling interval for Simple and Expanded Model
Patient ID : mgh126
Residuals Simple Model
Residuals(mmHg)
Pre-filling Interval(s)
Patient ID : mgh126
Residuals Expanded Model
Residuals(mmHg)
Pre-filling Interval(s)

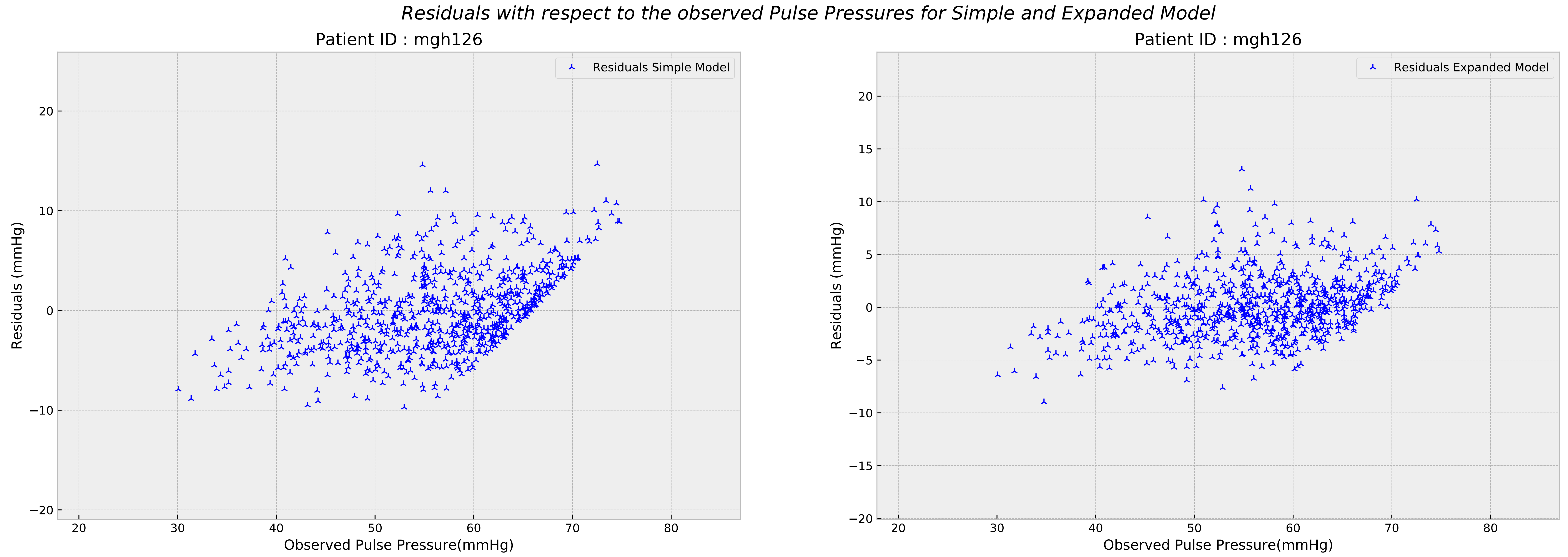
Residuals with respect to the observed Pulse Pressures for Simple and Expanded Model
Patient ID : mgh126
Residuals Simple Model
Residuals (mmHg)
Observed Pulse Pressure(mmHg)
20
10
0
−10
−20
20
30
40
50
60
70
80
Patient ID : mgh126
Residuals Expanded Model
Residuals (mmHg)
Observed Pulse Pressure(mmHg)
20
15
10
5
0
−5
−10
−15
−20
20
30
40
50
60
70
80

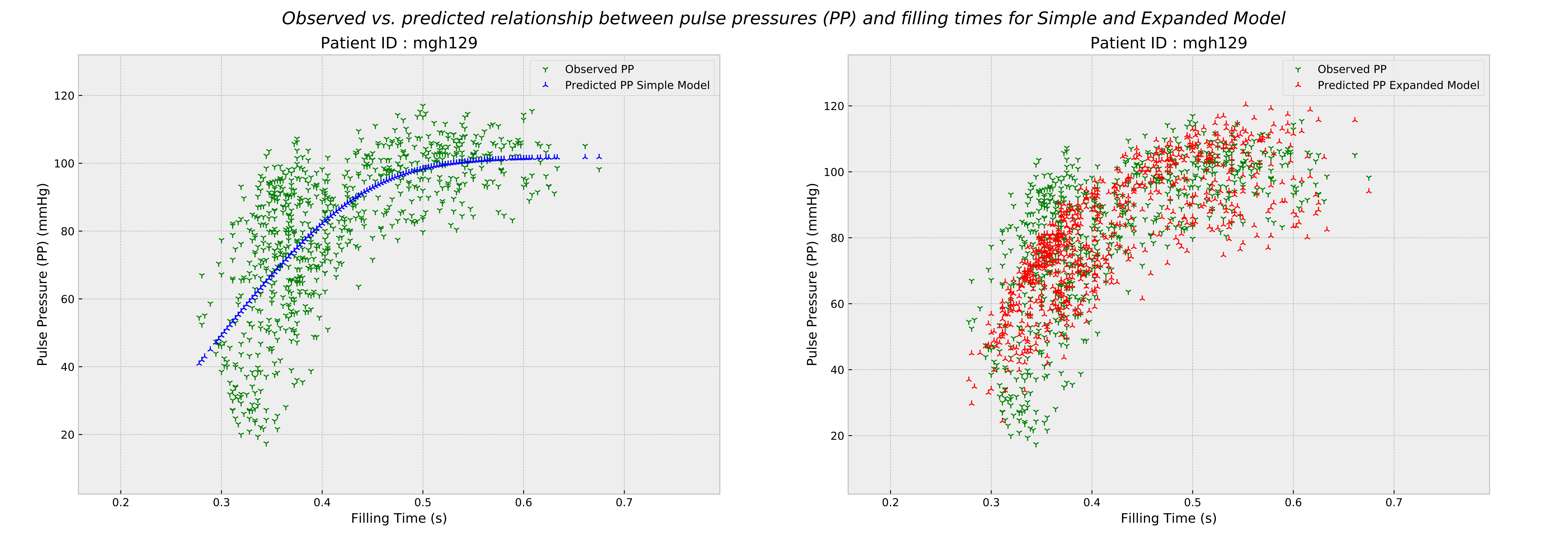
Observed vs. predicted relationship between pulse pressures (PP) and filling times for Simple and Expanded Model
Patient ID : mgh129
Observed PP
Predicted PP Simple Model
Pulse Pressure (PP) (mmHg)
Filling Time (s)
Patient ID : mgh129
Observed PP
Predicted PP Expanded Model
Pulse Pressure (PP) (mmHg)
Filling Time (s)

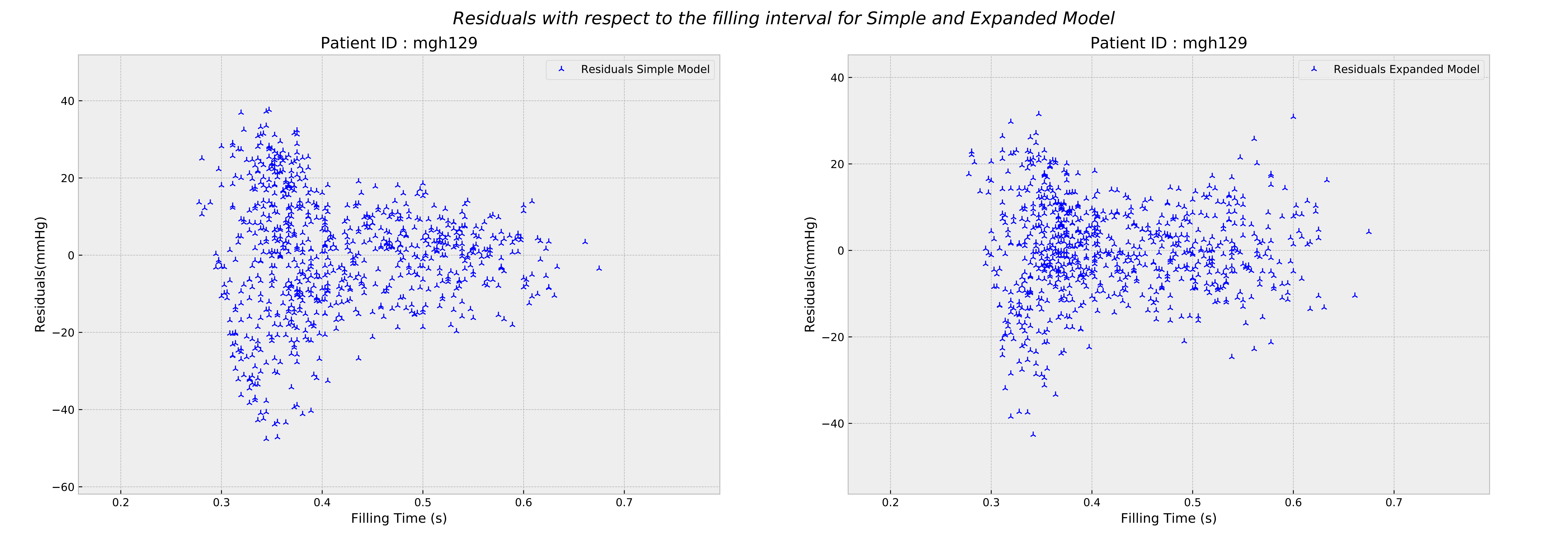

Residuals with respect to the filling interval for Simple and Expanded Model
Patient ID : mgh129
Residuals Simple Model
Residuals(mmHg)
40
20
0
−20
−40
−60
0.2
0.3
0.4
0.5
0.6
0.7
Filling Time (s)
Patient ID : mgh129
Residuals Expanded Model
Residuals(mmHg)
40
20
0
−20
−40
0.2
0.3
0.4
0.5
0.6
0.7
Filling Time (s)

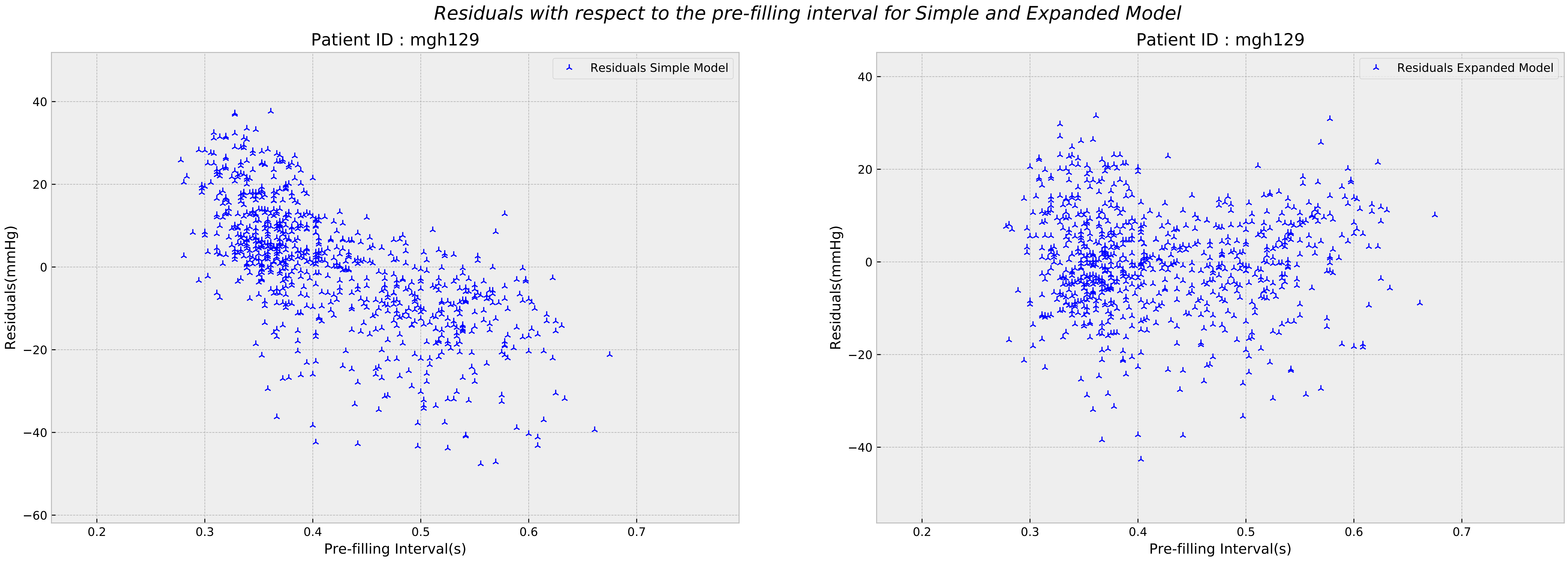
Residuals with respect to the pre-filling interval for Simple and Expanded Model
Patient ID : mgh129
Residuals Simple Model
Residuals(mmHg)
Pre-filling Interval(s)
40
20
0
−20
−40
−60
0.2
0.3
0.4
0.5
0.6
0.7
Patient ID : mgh129
Residuals Expanded Model
Residuals(mmHg)
Pre-filling Interval(s)
40
20
0
−20
−40
0.2
0.3
0.4
0.5
0.6
0.7

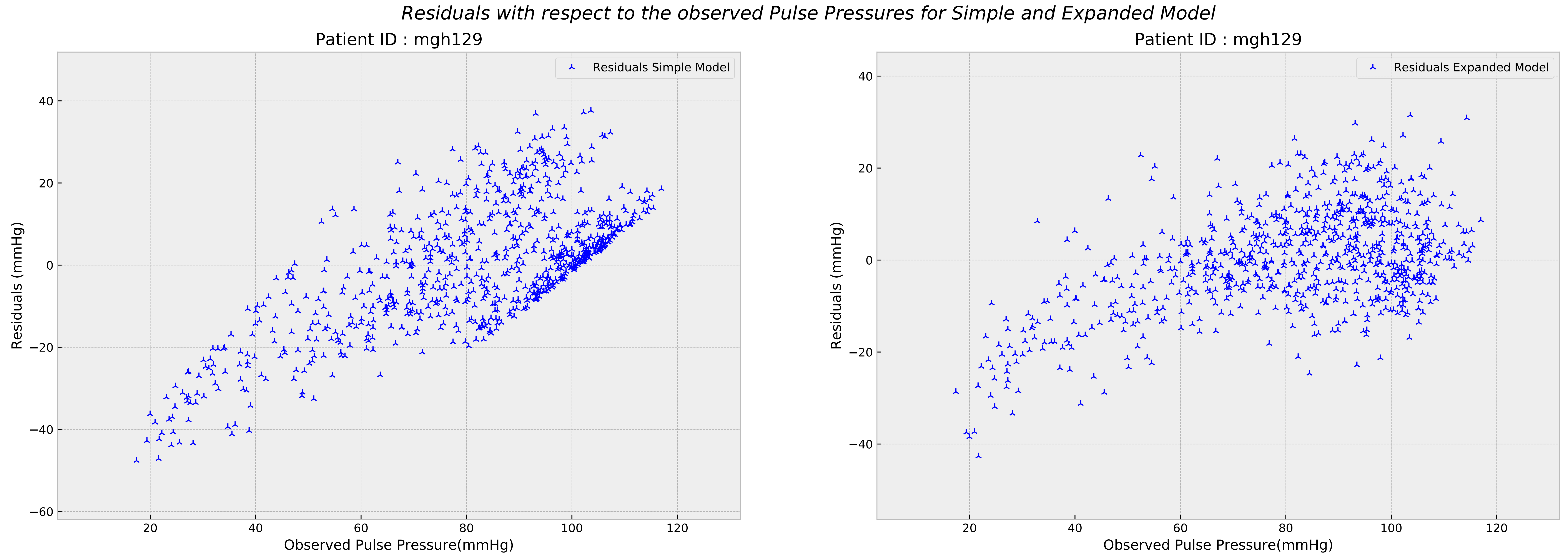
Residuals with respect to the observed Pulse Pressures for Simple and Expanded Model
Patient ID : mgh129
Residuals Simple Model
Residuals (mmHg)
Observed Pulse Pressure(mmHg)
40
20
0
−20
−40
−60
20
40
60
80
100
120
Patient ID : mgh129
Residuals Expanded Model
Residuals (mmHg)
Observed Pulse Pressure(mmHg)
40
20
0
−20
−40
20
40
60
80
100
120

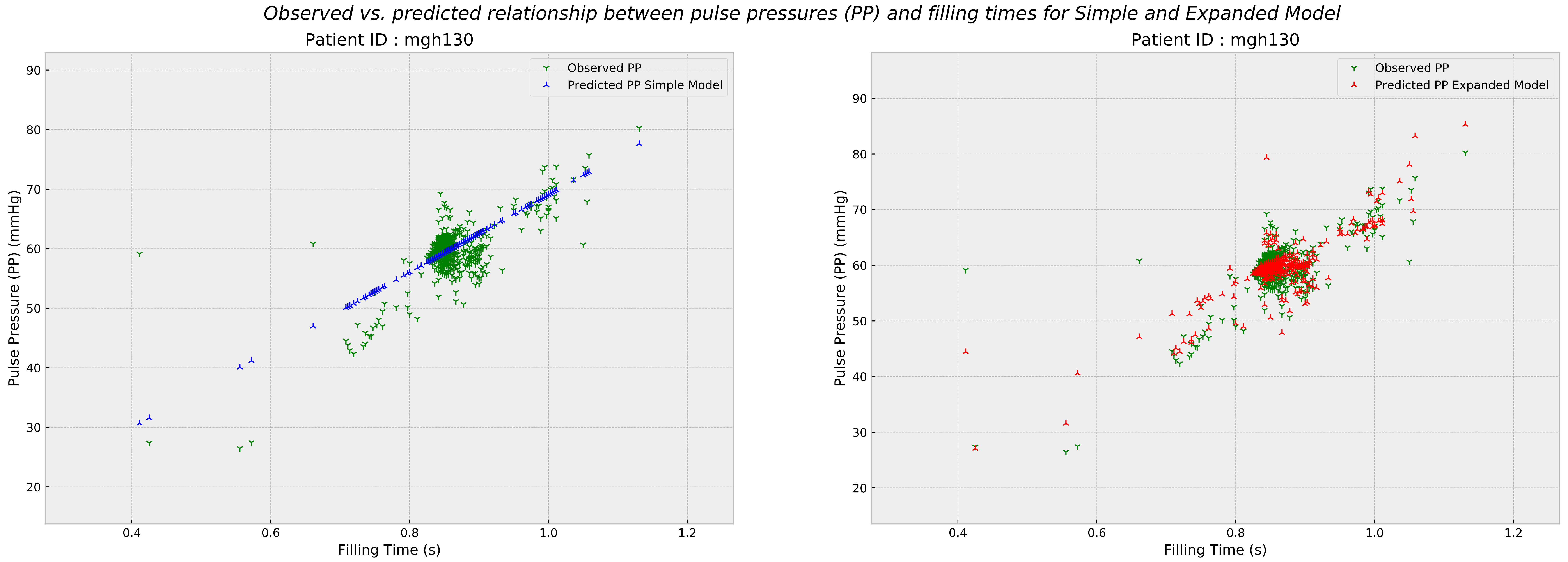
Observed vs. predicted relationship between pulse pressures (PP) and filling times for Simple and Expanded Model
Patient ID : mgh130
Observed PP
Predicted PP Simple Model
Pulse Pressure (PP) (mmHg)
Filling Time (s)
Patient ID : mgh130
Observed PP
Predicted PP Expanded Model
Pulse Pressure (PP) (mmHg)
Filling Time (s)

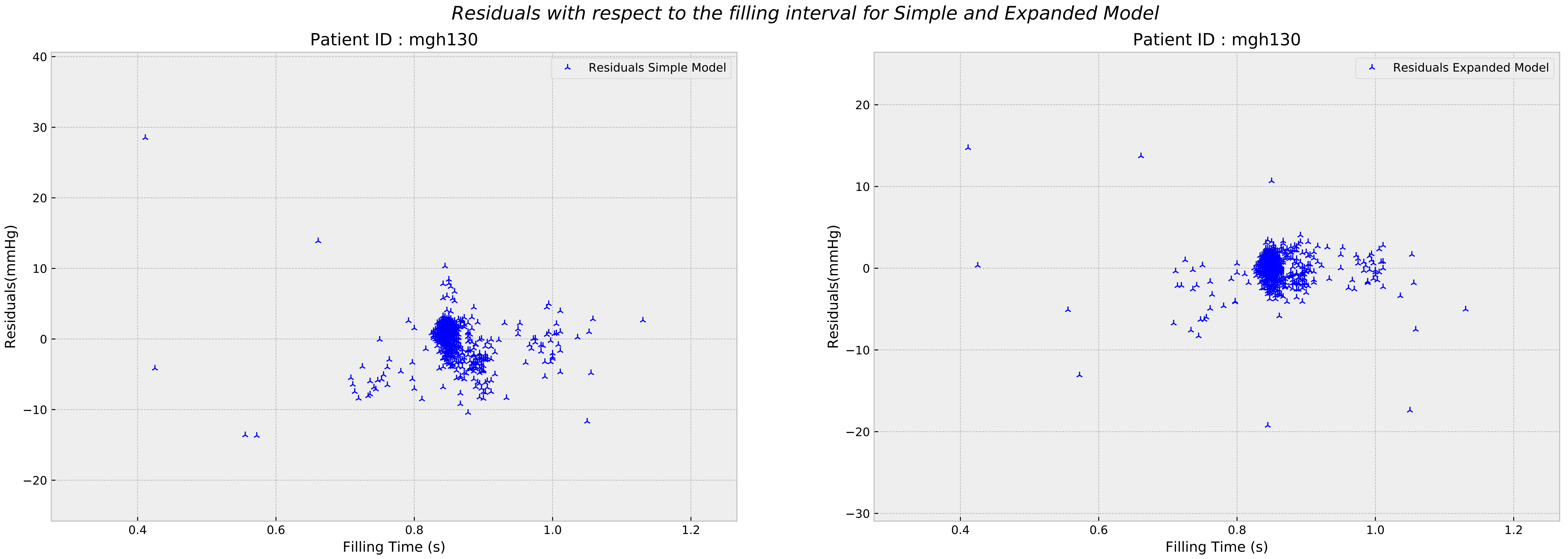
Residuals with respect to the filling interval for Simple and Expanded Model
Patient ID : mgh130
Residuals Simple Model
Residuals(mmHg)
Filling Time (s)
40
30
20
10
0
−10
−20
0.4
0.6
0.8
1.0
1.2
Patient ID : mgh130
Residuals Expanded Model
Residuals(mmHg)
Filling Time (s)
20
10
0
−10
−20
−30
0.4
0.6
0.8
1.0
1.2

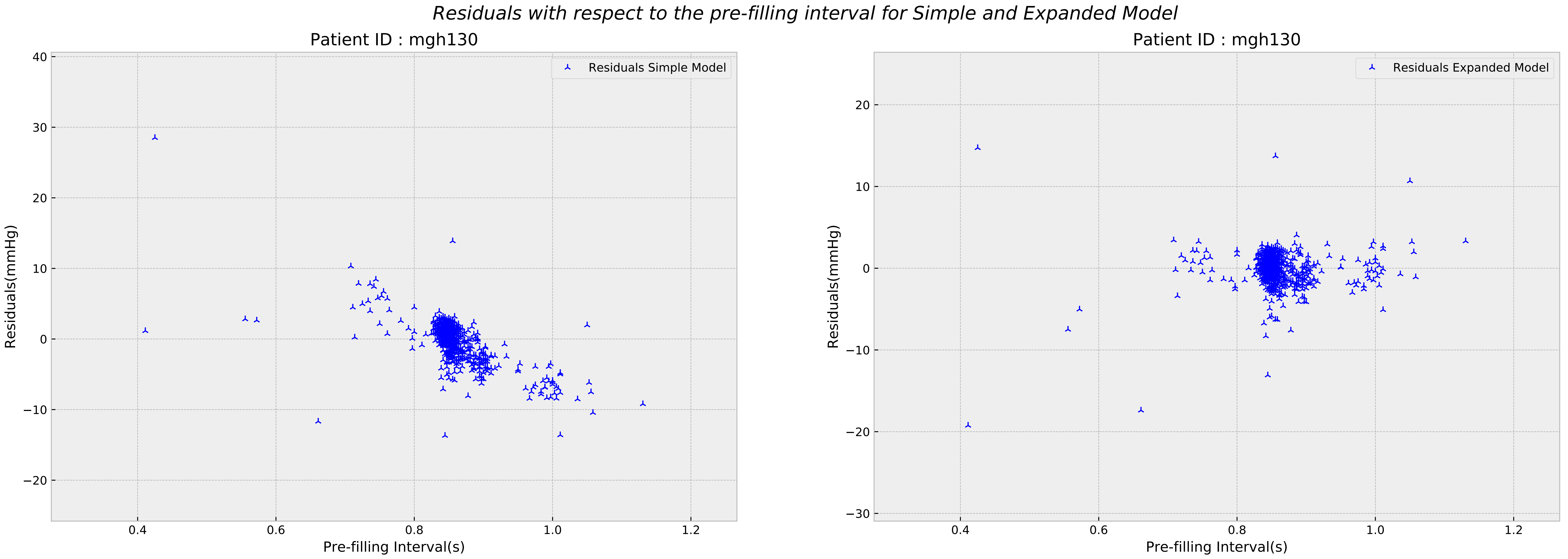
Residuals with respect to the pre-filling interval for Simple and Expanded Model
Patient ID : mgh130
Residuals Simple Model
Residuals(mmHg)
Pre-filling Interval(s)
40
30
20
10
0
−10
−20
0.4
0.6
0.8
1.0
1.2
Patient ID : mgh130
Residuals Expanded Model
Residuals(mmHg)
Pre-filling Interval(s)
20
10
0
−10
−20
−30
0.4
0.6
0.8
1.0
1.2

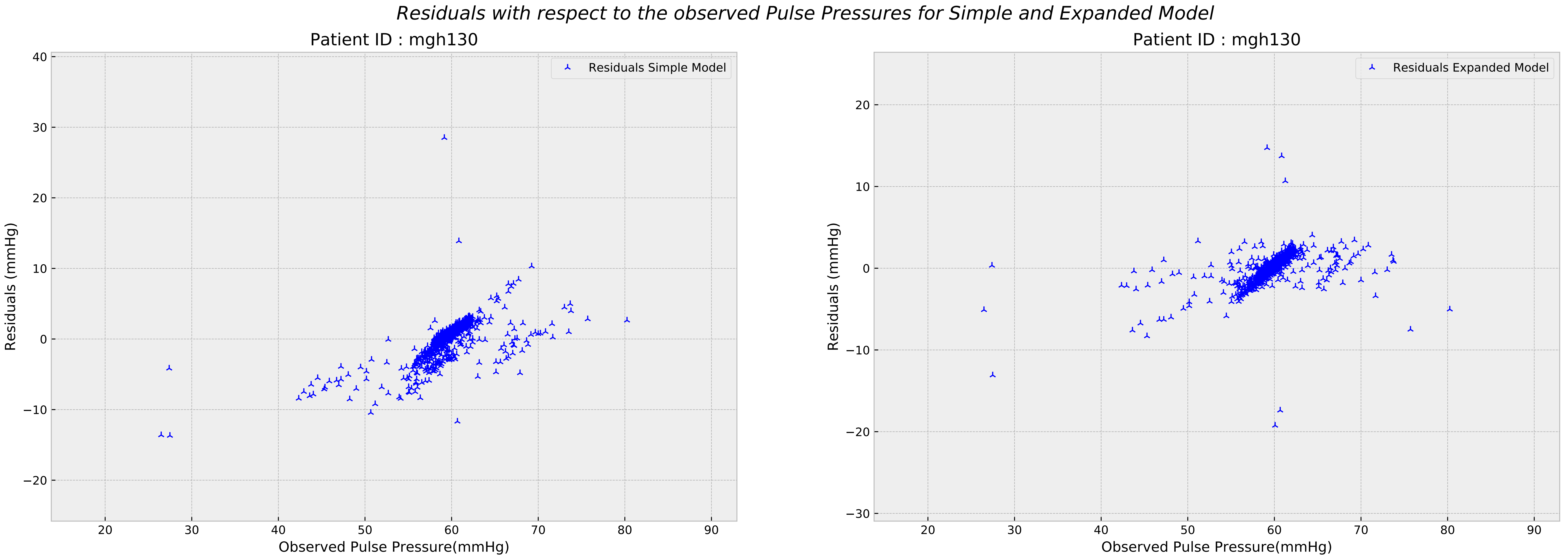

Residuals with respect to the observed Pulse Pressures for Simple and Expanded Model
Patient ID : mgh130
Residuals Simple Model
Residuals (mmHg)
Observed Pulse Pressure(mmHg)
40
30
20
10
0
−10
−20
20
30
40
50
60
70
80
90
Patient ID : mgh130
Residuals Expanded Model
Residuals (mmHg)
Observed Pulse Pressure(mmHg)
20
10
0
−10
−20
−30
20
30
40
50
60
70
80
90

Observed vs. predicted relationship between pulse pressures (PP) and filling times for Simple and Expanded Model

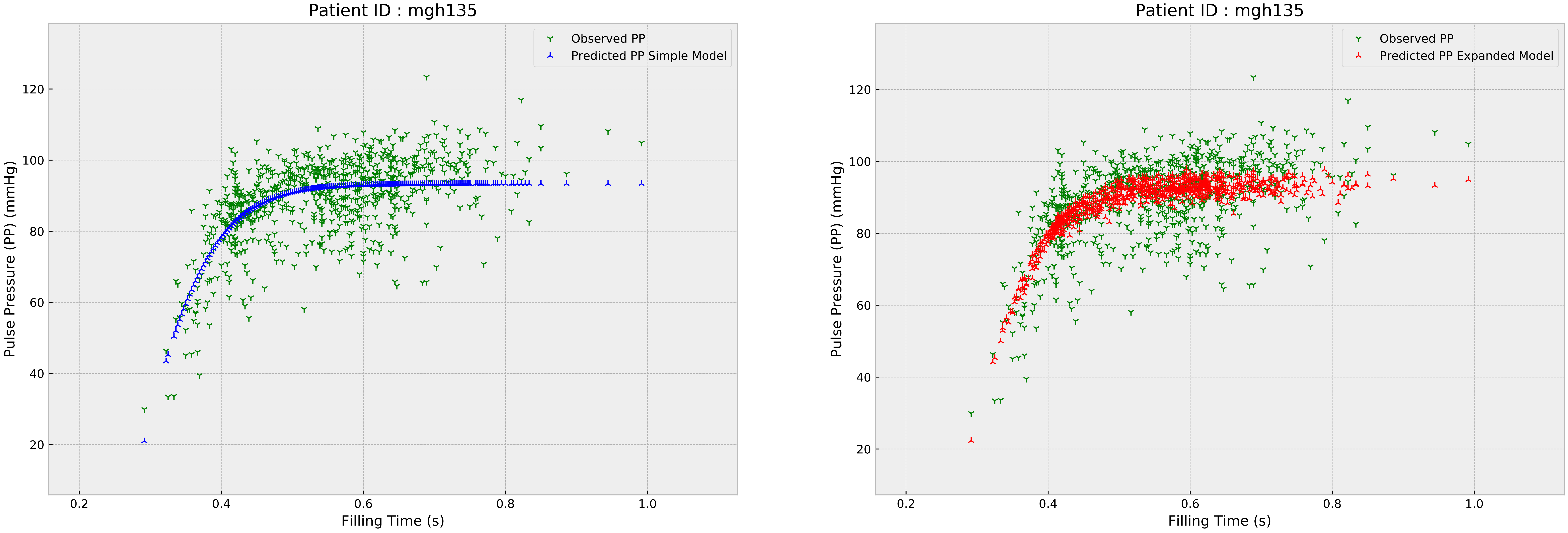

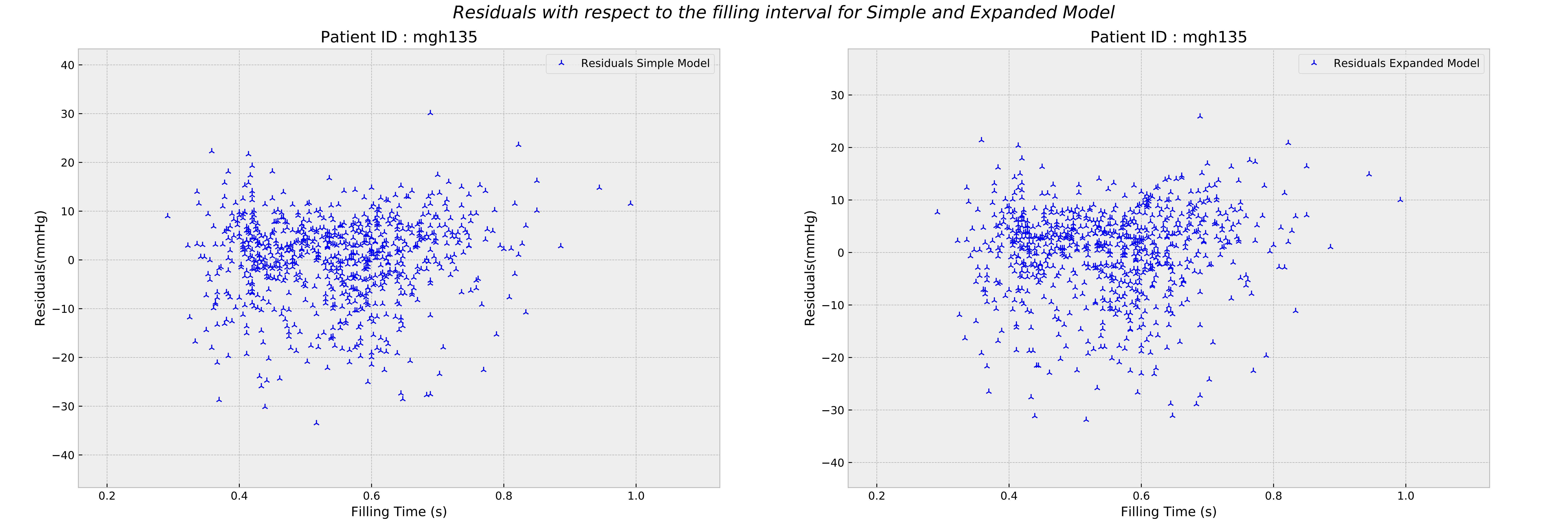
Residuals with respect to the filling interval for Simple and Expanded Model
Patient ID : mgh135
Residuals Simple Model
Residuals(mmHg)
Filling Time (s)
40
30
20
10
0
−10
−20
−30
−40
0.2
0.4
0.6
0.8
1.0
Patient ID : mgh135
Residuals Expanded Model
Residuals(mmHg)
Filling Time (s)
30
20
10
0
−10
−20
−30
−40
0.2
0.4
0.6
0.8
1.0

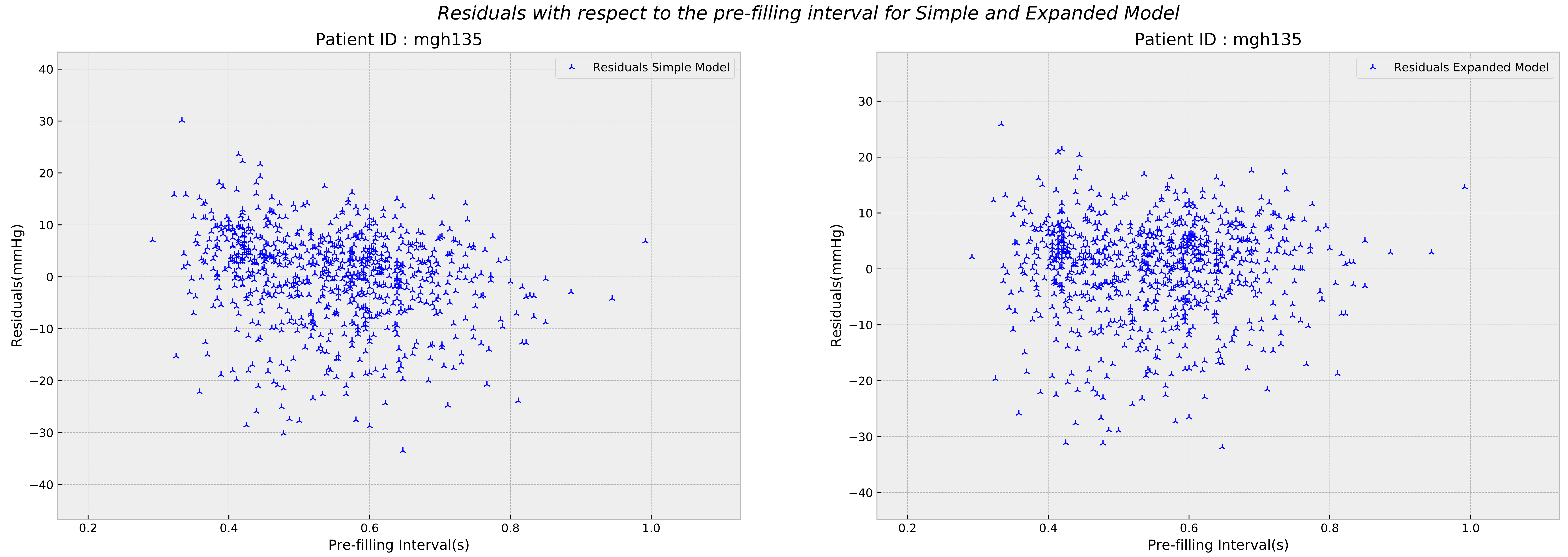

Residuals with respect to the pre-filling interval for Simple and Expanded Model
Patient ID : mgh135
Residuals Simple Model
Residuals(mmHg)
Pre-filling Interval(s)
Patient ID : mgh135
Residuals Expanded Model
Residuals(mmHg)
Pre-filling Interval(s)

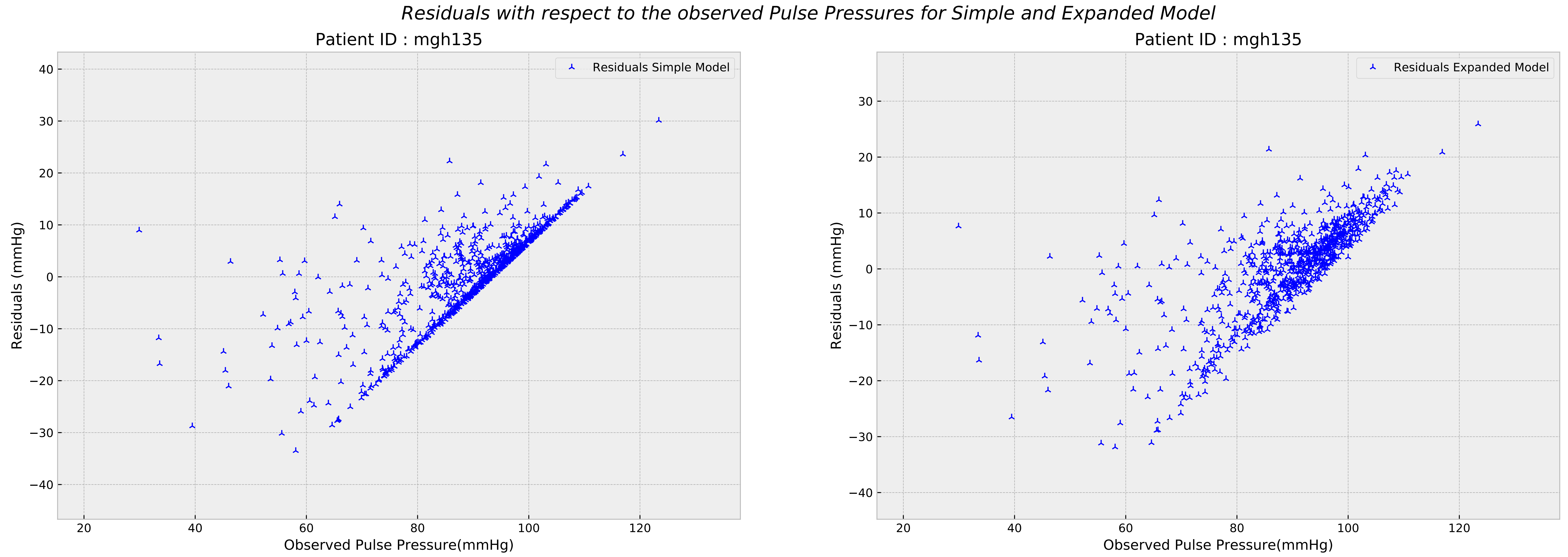
Residuals with respect to the observed Pulse Pressures for Simple and Expanded Model
Patient ID : mgh135
Residuals Simple Model
Residuals (mmHg)
Observed Pulse Pressure(mmHg)
40
30
20
10
0
−10
−20
−30
−40
20
40
60
80
100
120
Patient ID : mgh135
Residuals Expanded Model
Residuals (mmHg)
Observed Pulse Pressure(mmHg)
30
20
10
0
−10
−20
−30
−40
20
40
60
80
100
120

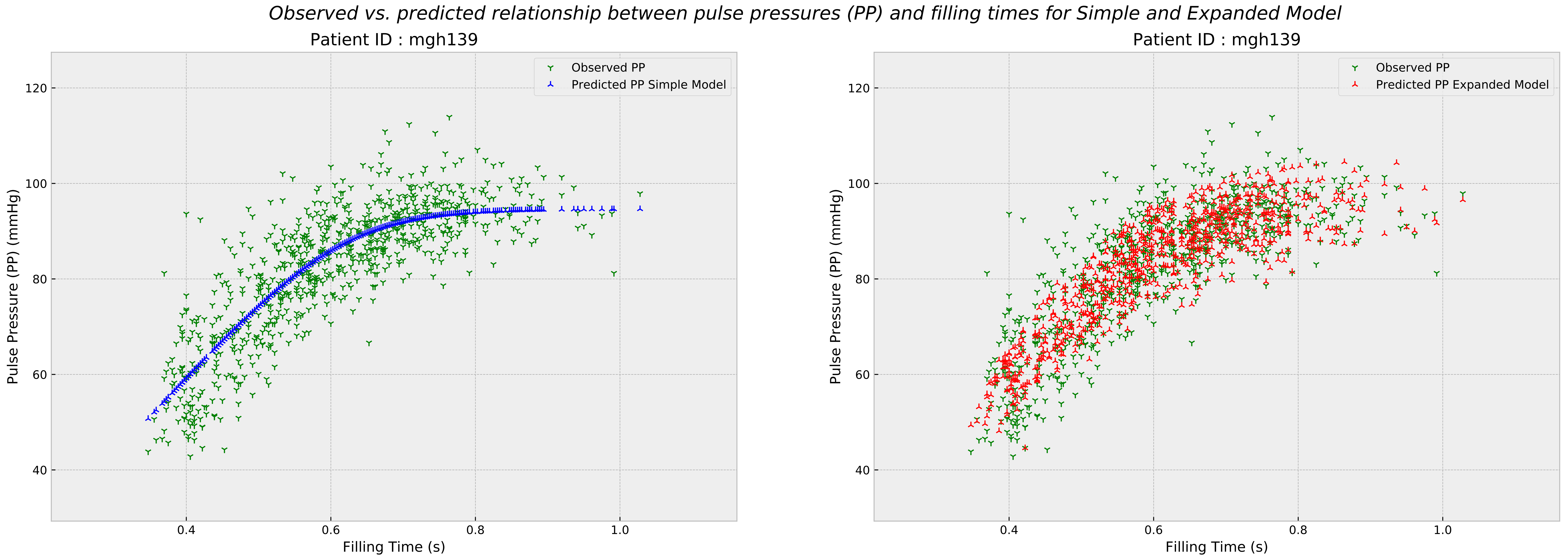
Observed vs. predicted relationship between pulse pressures (PP) and filling times for Simple and Expanded Model
Patient ID : mgh139
Observed PP
Predicted PP Simple Model
Pulse Pressure (PP) (mmHg)
Filling Time (s)
Patient ID : mgh139
Observed PP
Predicted PP Expanded Model
Pulse Pressure (PP) (mmHg)
Filling Time (s)

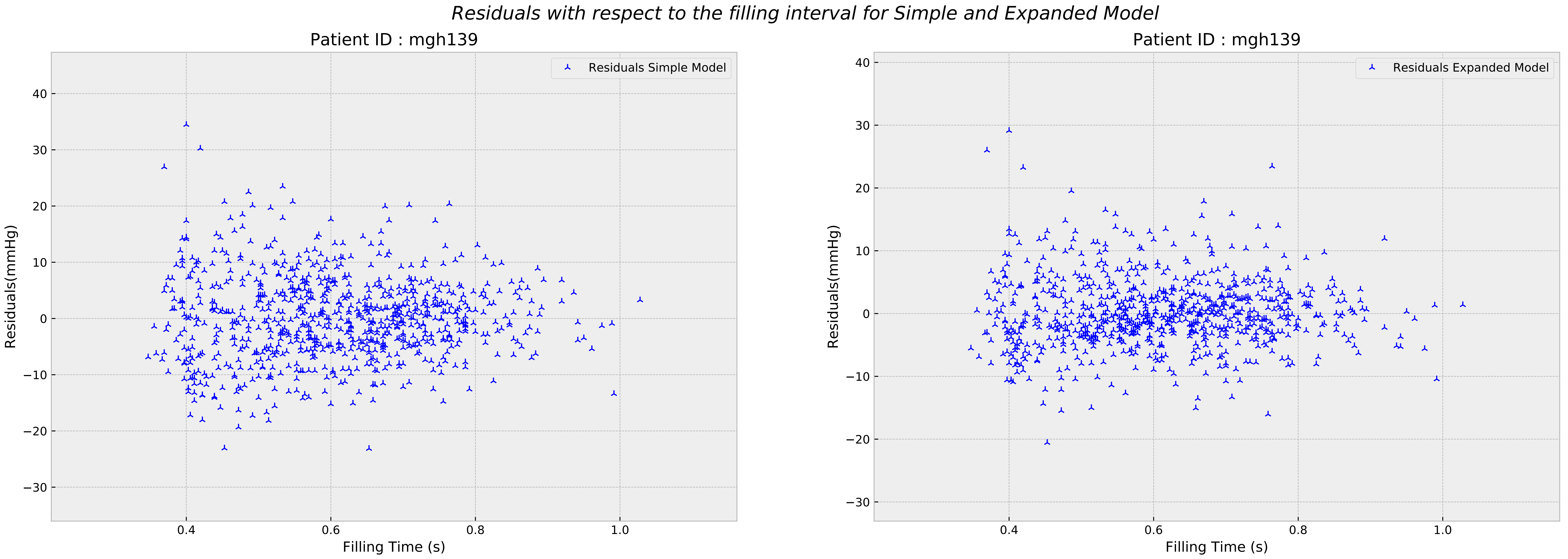

Residuals with respect to the filling interval for Simple and Expanded Model
Patient ID : mgh139
Residuals Simple Model
Residuals(mmHg)
Filling Time (s)
Patient ID : mgh139
Residuals Expanded Model
Residuals(mmHg)
Filling Time (s)

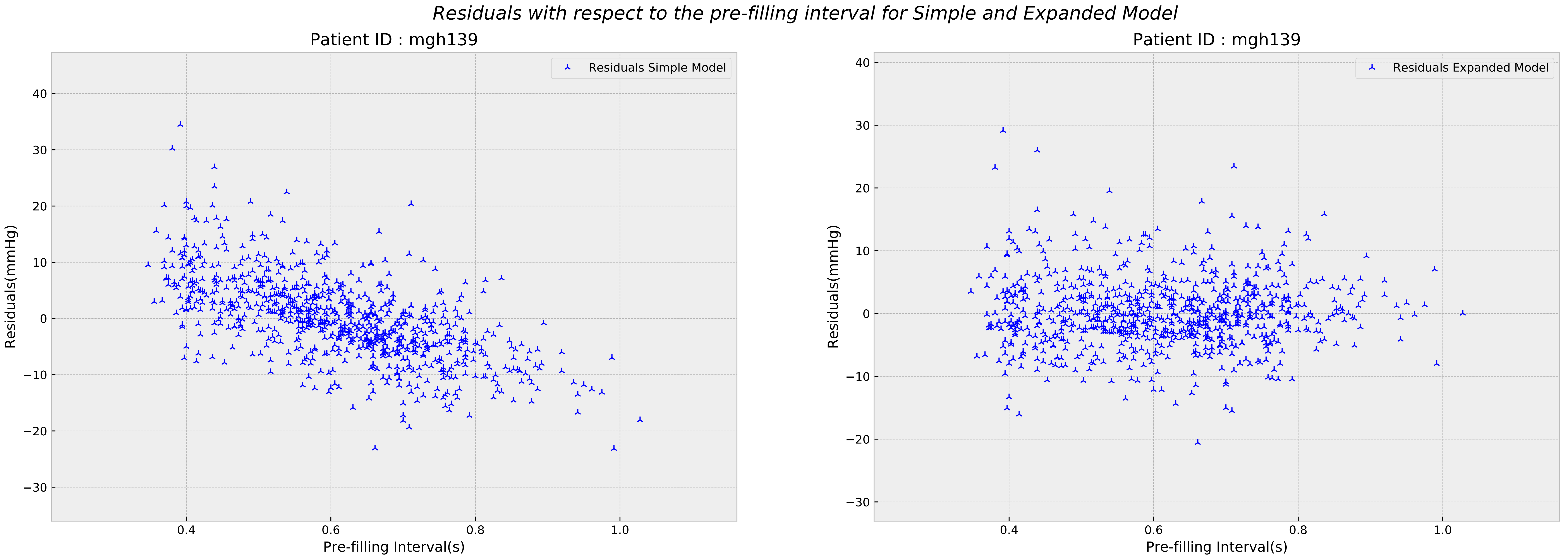
Residuals with respect to the pre-filling interval for Simple and Expanded Model
Patient ID : mgh139
Residuals Simple Model
Residuals(mmHg)
Pre-filling Interval(s)
40
30
20
10
0
−10
−20
−30
0.4
0.6
0.8
1.0
Patient ID : mgh139
Residuals Expanded Model
Residuals(mmHg)
Pre-filling Interval(s)
40
30
20
10
0
−10
−20
−30
0.4
0.6
0.8
1.0

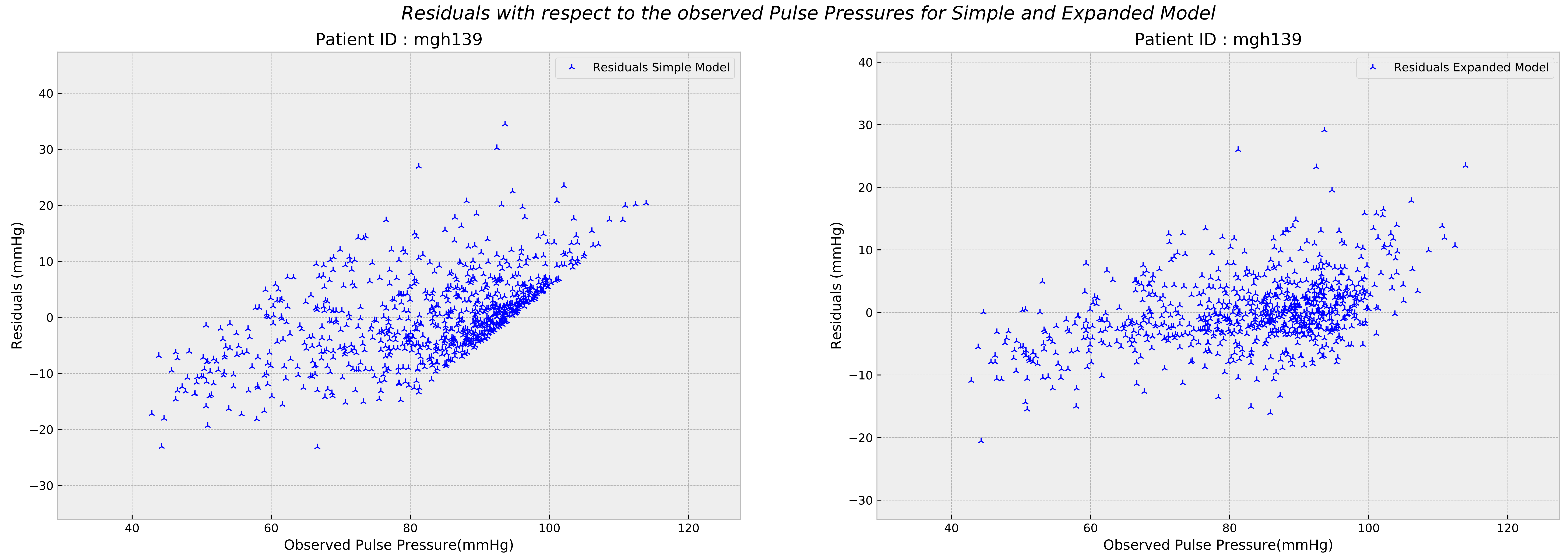
Residuals with respect to the observed Pulse Pressures for Simple and Expanded Model
Patient ID : mgh139
Residuals Simple Model
Residuals (mmHg)
Observed Pulse Pressure(mmHg)
40
30
20
10
0
−10
−20
−30
40
60
80
100
120
Patient ID : mgh139
Residuals Expanded Model
Residuals (mmHg)
Observed Pulse Pressure(mmHg)
40
30
20
10
0
−10
−20
−30
40
60
80
100
120

Observed vs. predicted relationship between pulse pressures (PP) and filling times for Simple and Expanded Model

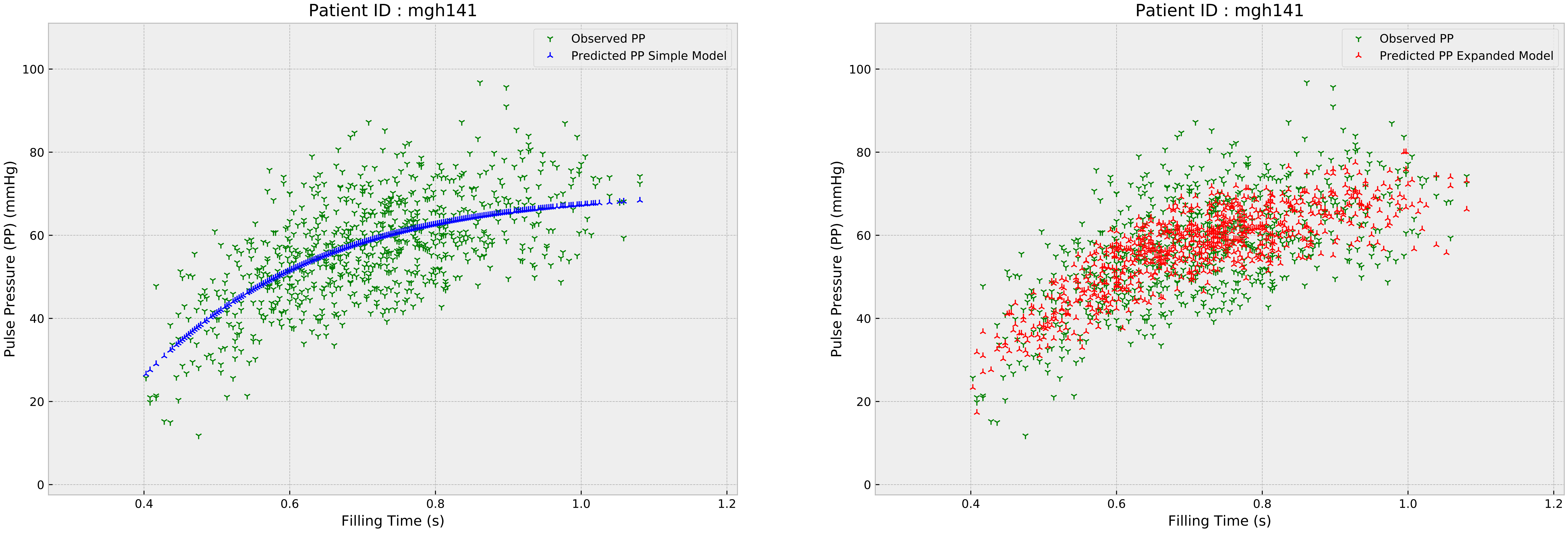

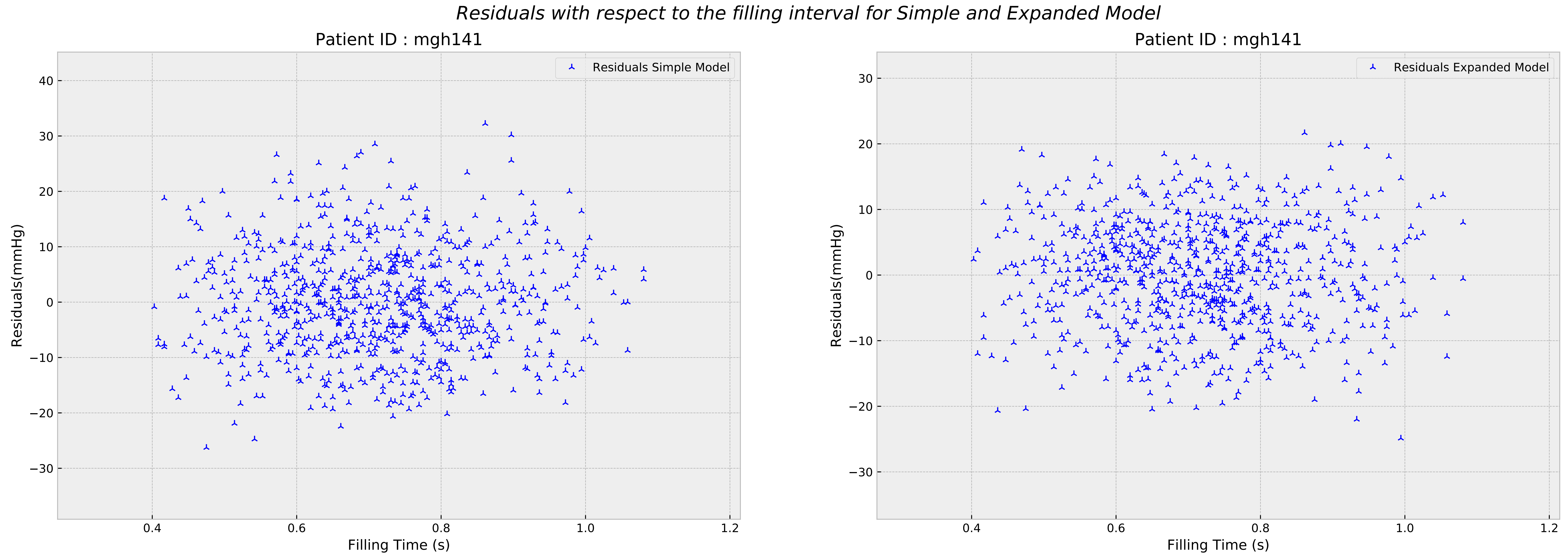

Residuals with respect to the filling interval for Simple and Expanded Model
Patient ID : mgh141
Residuals Simple Model
Residuals(mmHg)
Filling Time (s)
40
30
20
10
0
−10
−20
−30
0.4
0.6
0.8
1.0
1.2
Patient ID : mgh141
Residuals Expanded Model
Residuals(mmHg)
Filling Time (s)
30
20
10
0
−10
−20
−30
0.4
0.6
0.8
1.0
1.2

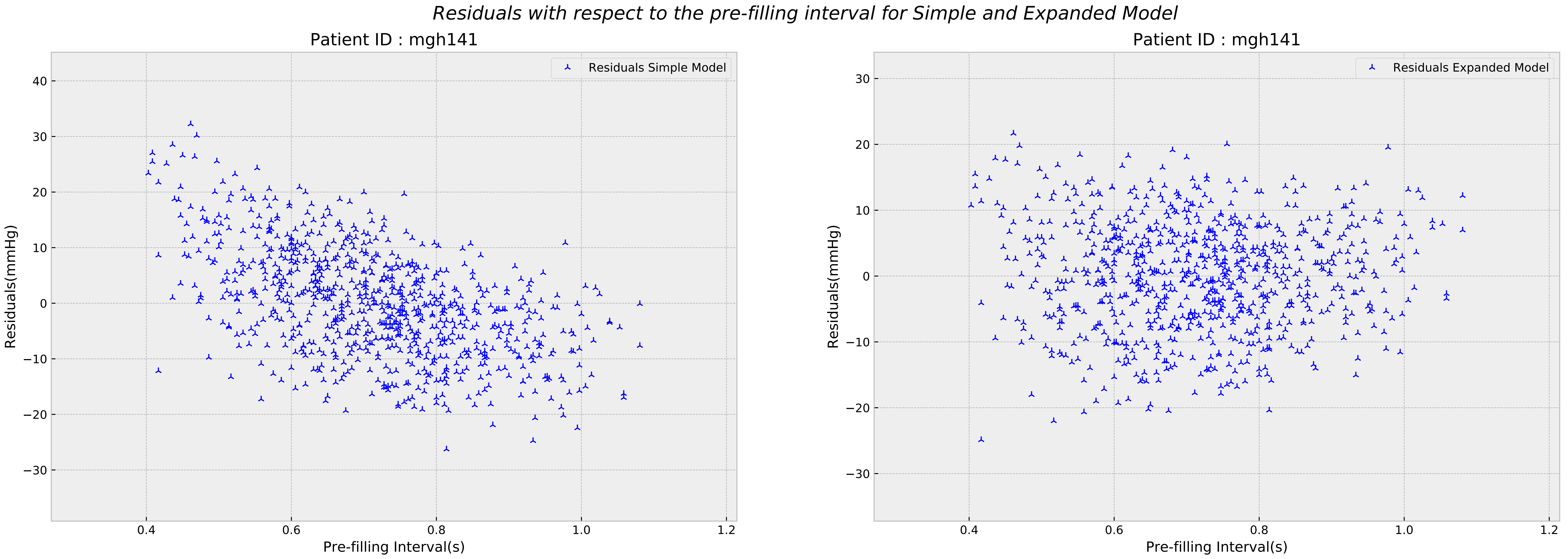
Residuals with respect to the pre-filling interval for Simple and Expanded Model
Patient ID : mgh141
Residuals Simple Model
Residuals(mmHg)
Pre-filling Interval(s)
40
30
20
10
0
−10
−20
−30
0.4
0.6
0.8
1.0
1.2
Patient ID : mgh141
Residuals Expanded Model
Residuals(mmHg)
Pre-filling Interval(s)
30
20
10
0
−10
−20
−30
0.4
0.6
0.8
1.0
1.2

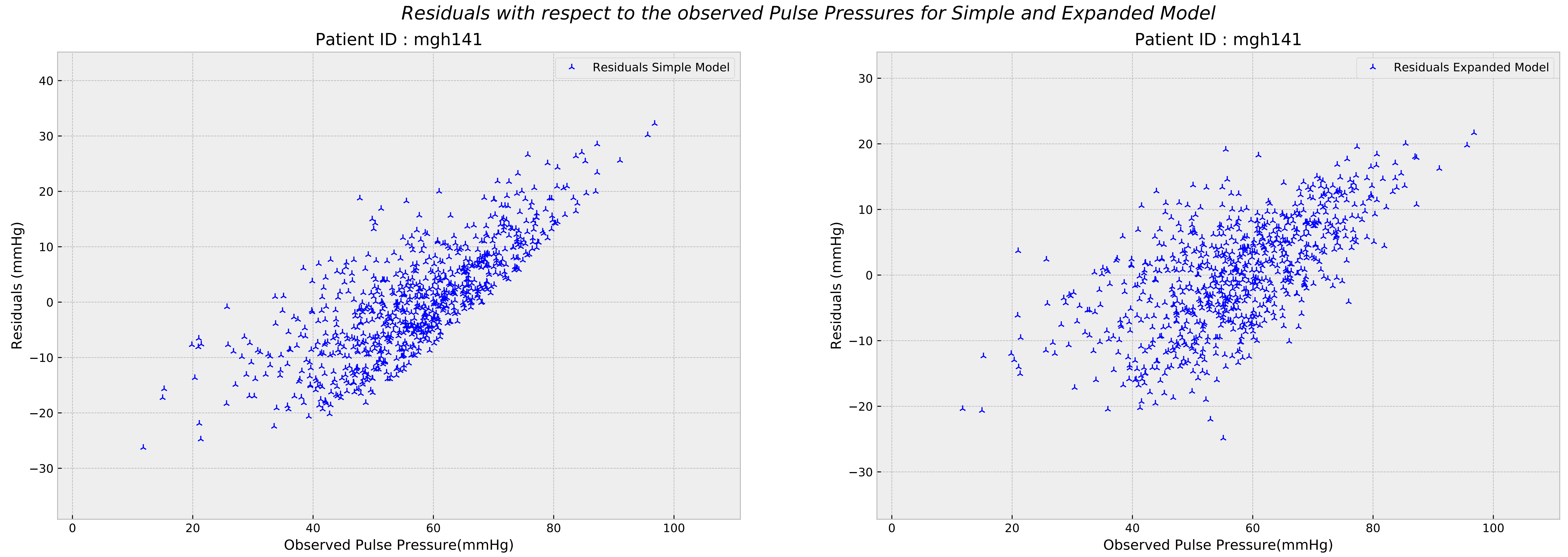
Residuals with respect to the observed Pulse Pressures for Simple and Expanded Model
Patient ID : mgh141
Residuals Simple Model
Residuals (mmHg)
Observed Pulse Pressure(mmHg)
Patient ID : mgh141
Residuals Expanded Model
Residuals (mmHg)
Observed Pulse Pressure(mmHg)

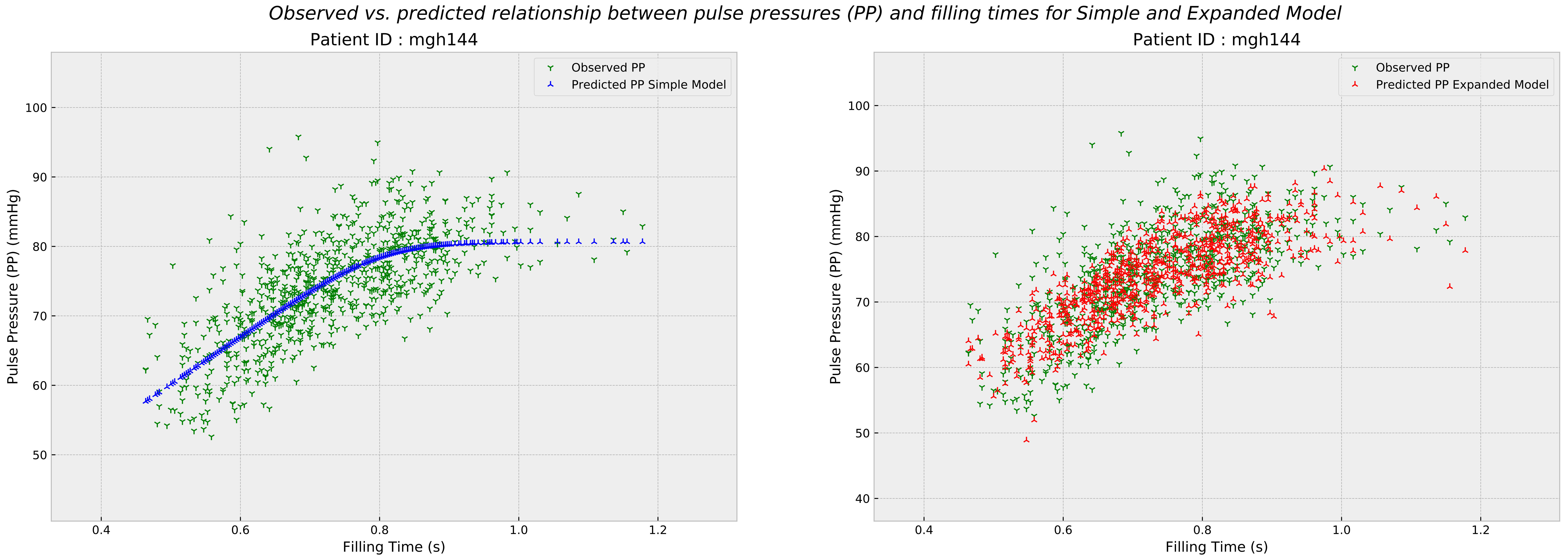
Observed vs. predicted relationship between pulse pressures (PP) and filling times for Simple and Expanded Model
Patient ID : mgh144
Observed PP
Predicted PP Simple Model
Pulse Pressure (PP) (mmHg)
Filling Time (s)
Patient ID : mgh144
Observed PP
Predicted PP Expanded Model
Pulse Pressure (PP) (mmHg)
Filling Time (s)

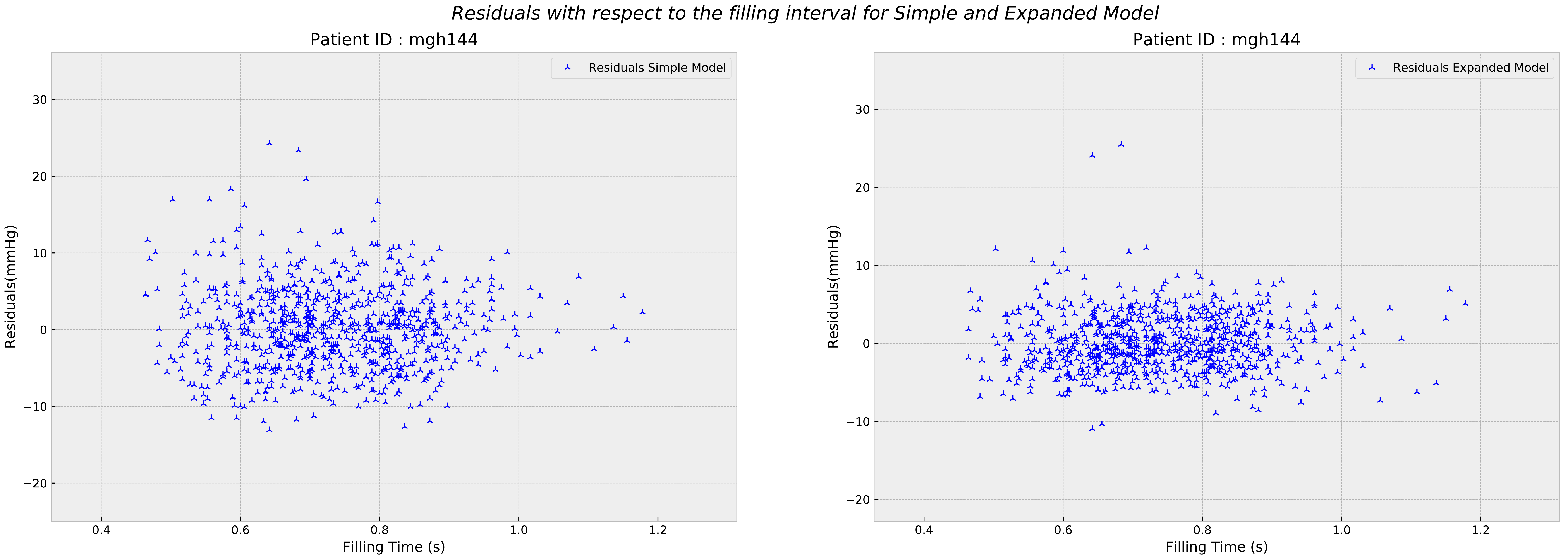
Residuals with respect to the filling interval for Simple and Expanded Model
Patient ID : mgh144
Residuals Simple Model
Residuals(mmHg)
30
20
10
0
−10
−20
0.4
0.6
0.8
1.0
1.2
Filling Time (s)
Patient ID : mgh144
Residuals Expanded Model
Residuals(mmHg)
30
20
10
0
−10
−20
0.4
0.6
0.8
1.0
1.2
Filling Time (s)

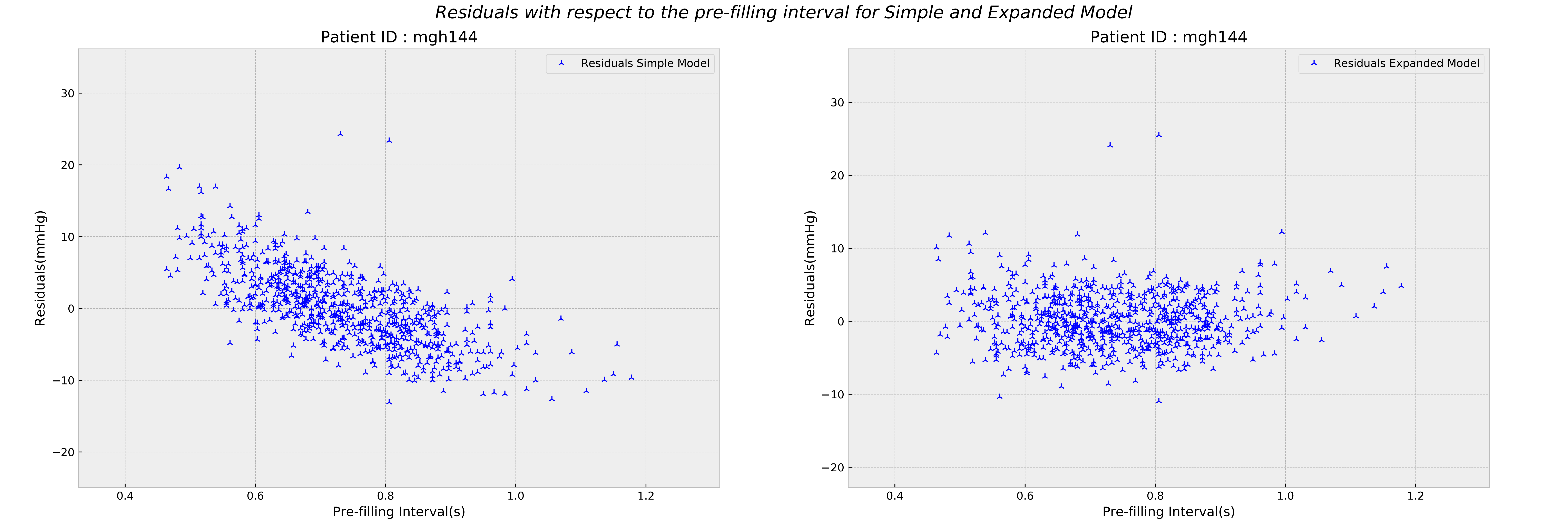
Residuals with respect to the pre-filling interval for Simple and Expanded Model
Patient ID : mgh144
Residuals Simple Model
Residuals(mmHg)
Pre-filling Interval(s)
30
20
10
0
−10
−20
0.4
0.6
0.8
1.0
1.2
Patient ID : mgh144
Residuals Expanded Model
Residuals(mmHg)
Pre-filling Interval(s)
30
20
10
0
−10
−20
0.4
0.6
0.8
1.0
1.2

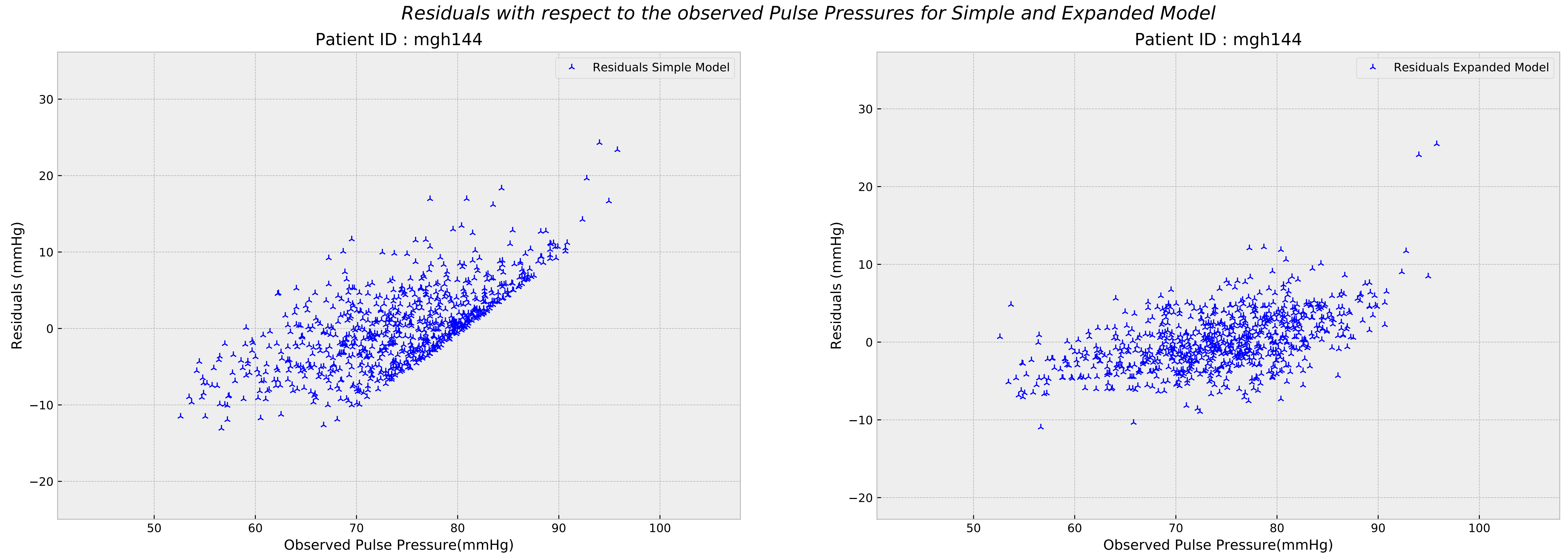
Residuals with respect to the observed Pulse Pressures for Simple and Expanded Model
Patient ID : mgh144
Residuals Simple Model
Residuals (mmHg)
Observed Pulse Pressure(mmHg)
30
20
10
0
−10
−20
50
60
70
80
90
100
Patient ID : mgh144
Residuals Expanded Model
Residuals (mmHg)
Observed Pulse Pressure(mmHg)

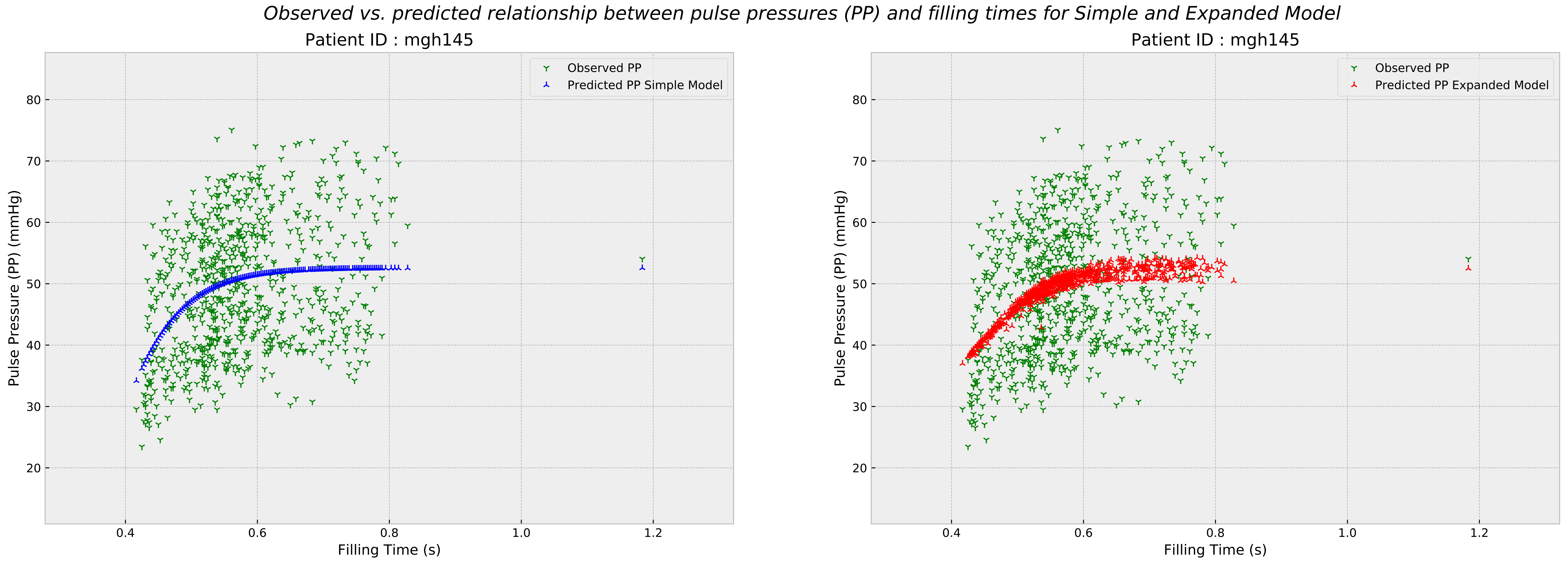
Observed vs. predicted relationship between pulse pressures (PP) and filling times for Simple and Expanded Model
Patient ID : mgh145
Observed PP
Predicted PP Simple Model
Pulse Pressure (PP) (mmHg)
Filling Time (s)
20
30
40
50
60
70
80
0.4
0.6
0.8
1.0
1.2
Patient ID : mgh145
Observed PP
Predicted PP Expanded Model
Pulse Pressure (PP) (mmHg)
Filling Time (s)

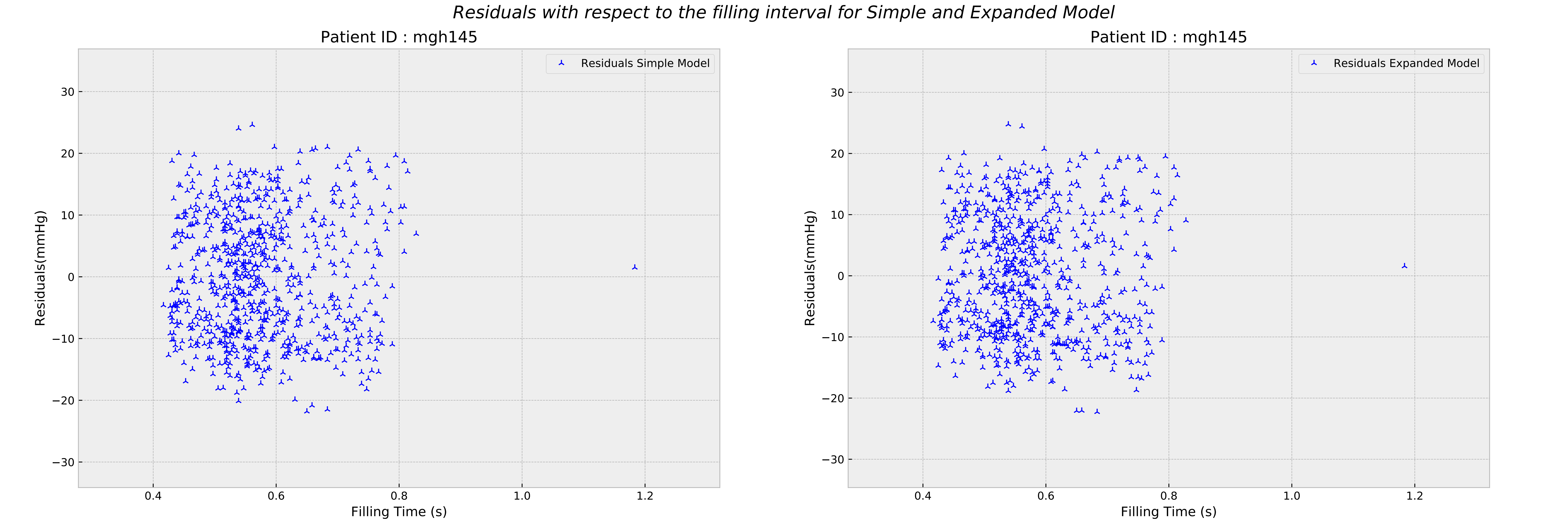
Residuals with respect to the filling interval for Simple and Expanded Model
Patient ID : mgh145
Residuals Simple Model
Residuals(mmHg)
Filling Time (s)
Patient ID : mgh145
Residuals Expanded Model
Residuals(mmHg)
Filling Time (s)

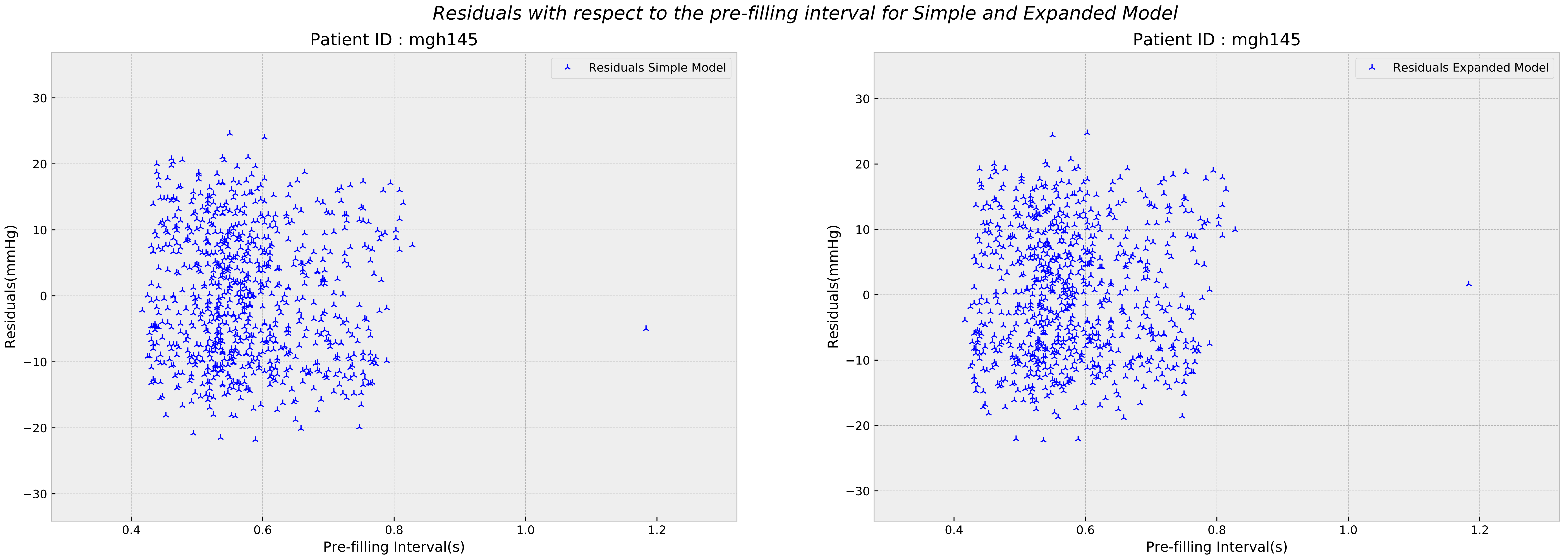

Residuals with respect to the pre-filling interval for Simple and Expanded Model
Patient ID : mgh145
Residuals Simple Model
Residuals(mmHg)
Pre-filling Interval(s)
30
20
10
0
−10
−20
−30
0.4
0.6
0.8
1.0
1.2
Patient ID : mgh145
Residuals Expanded Model
Residuals(mmHg)
Pre-filling Interval(s)

Residuals with respect to the observed Pulse Pressures for Simple and Expanded Model

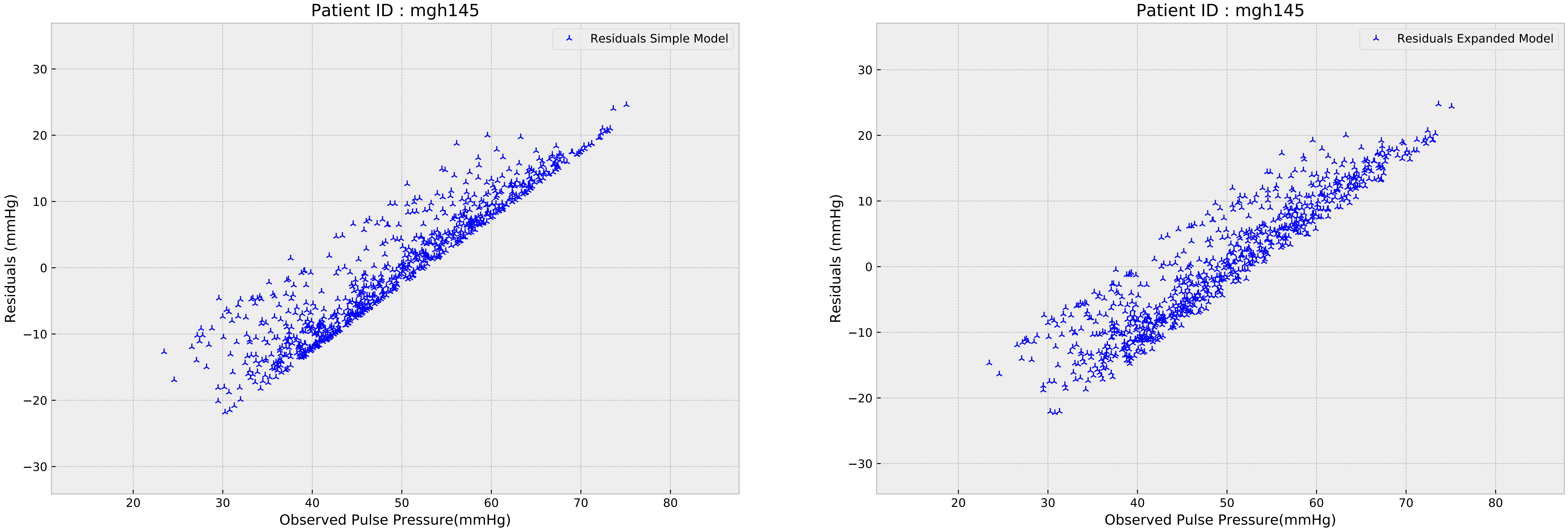

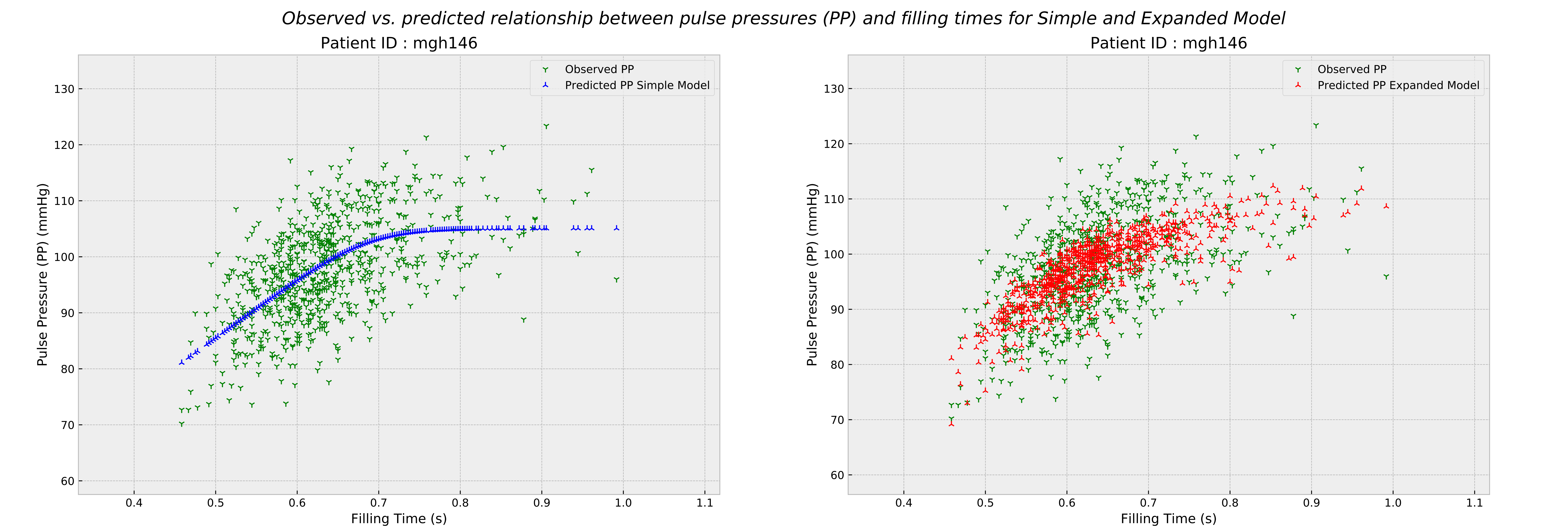
Observed vs. predicted relationship between pulse pressures (PP) and filling times for Simple and Expanded Model
Patient ID : mgh146
Observed PP
Predicted PP Simple Model
Pulse Pressure (PP) (mmHg)
Filling Time (s)
Patient ID : mgh146
Observed PP
Predicted PP Expanded Model
Pulse Pressure (PP) (mmHg)
Filling Time (s)

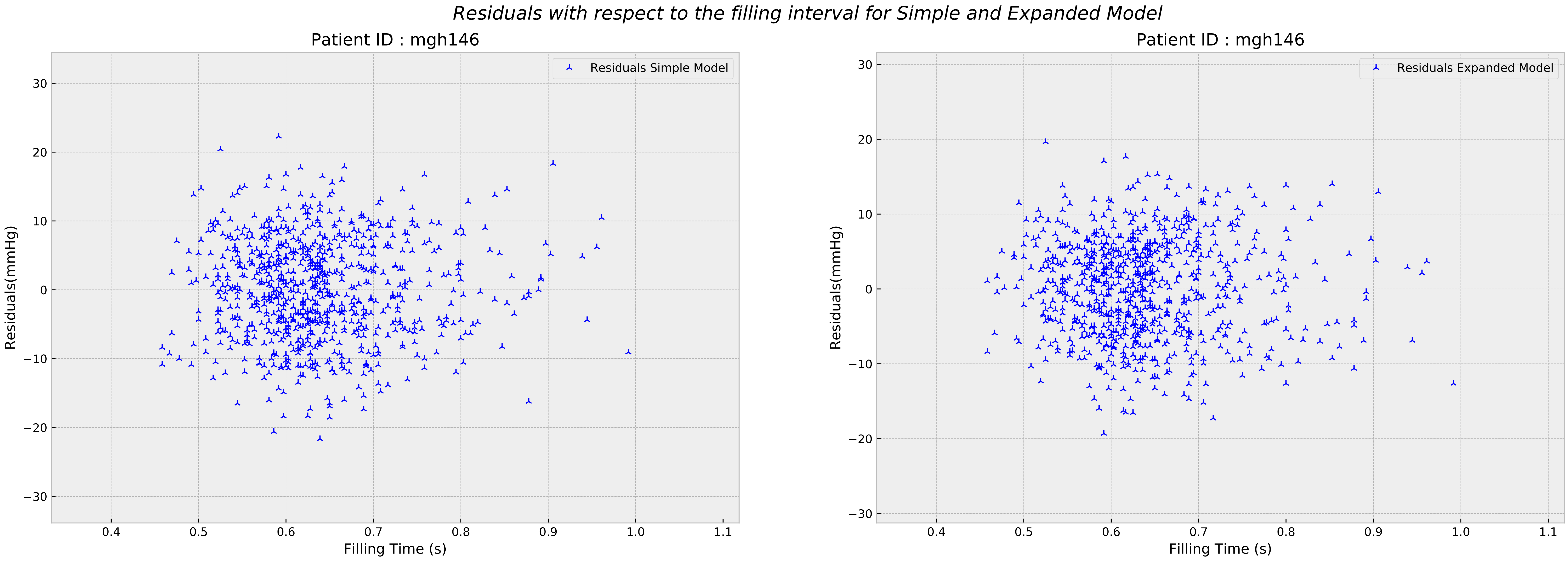

Residuals with respect to the filling interval for Simple and Expanded Model
Patient ID : mgh146
Residuals Simple Model
Residuals(mmHg)
Filling Time (s)
Patient ID : mgh146
Residuals Expanded Model
Residuals(mmHg)
Filling Time (s)

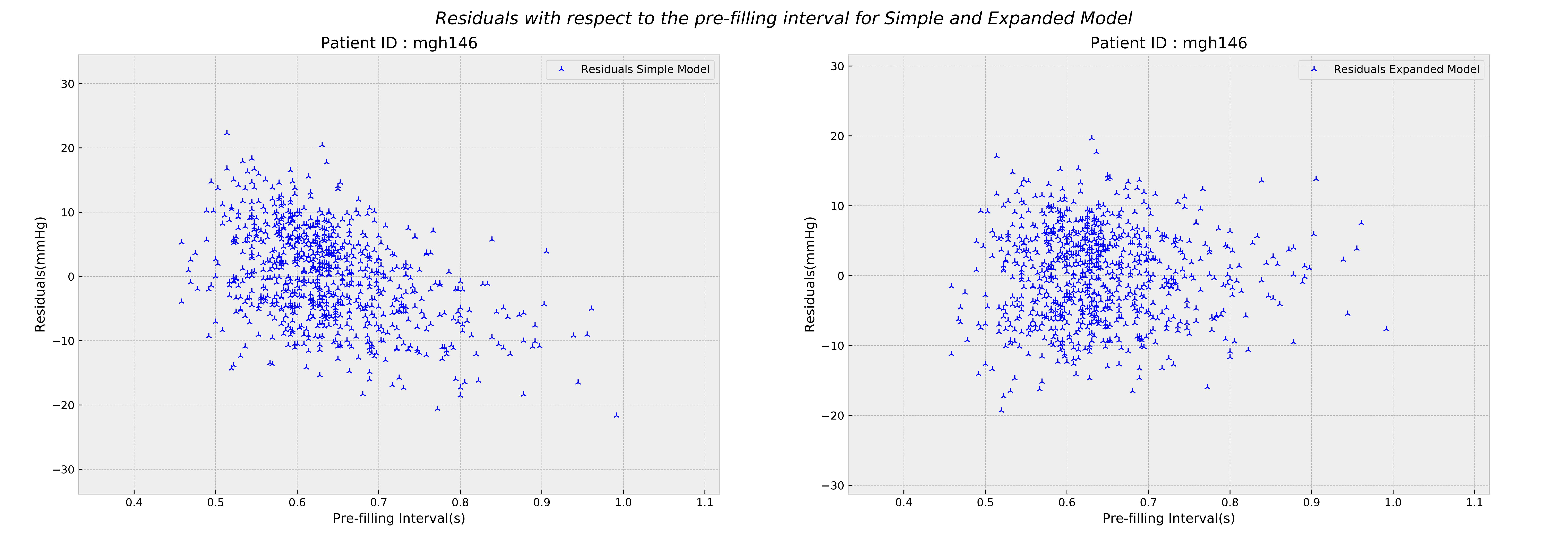
Residuals with respect to the pre-filling interval for Simple and Expanded Model
Patient ID : mgh146
Residuals Simple Model
Residuals(mmHg)
Pre-filling Interval(s)
Patient ID : mgh146
Residuals Expanded Model
Residuals(mmHg)
Pre-filling Interval(s)

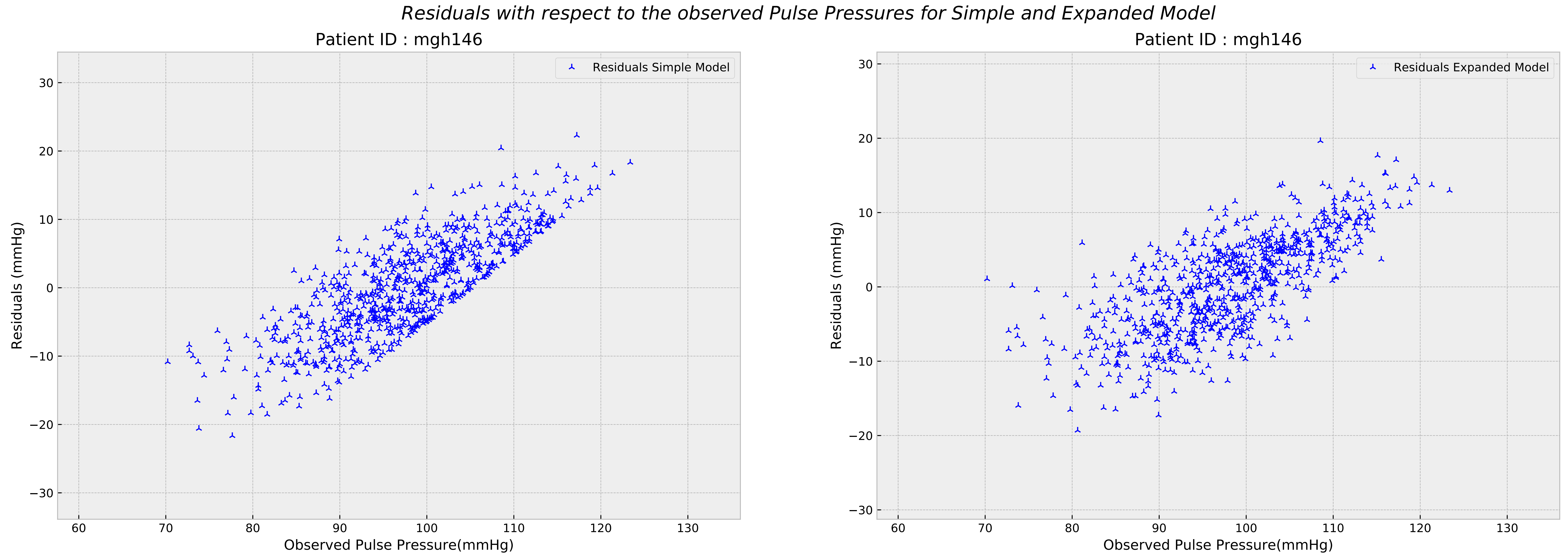

Residuals with respect to the observed Pulse Pressures for Simple and Expanded Model
Patient ID : mgh146
Residuals Simple Model
Residuals (mmHg)
Observed Pulse Pressure(mmHg)
Patient ID : mgh146
Residuals Expanded Model
Residuals (mmHg)
Observed Pulse Pressure(mmHg)

Observed vs. predicted relationship between pulse pressures (PP) and filling times for Simple and Expanded Model

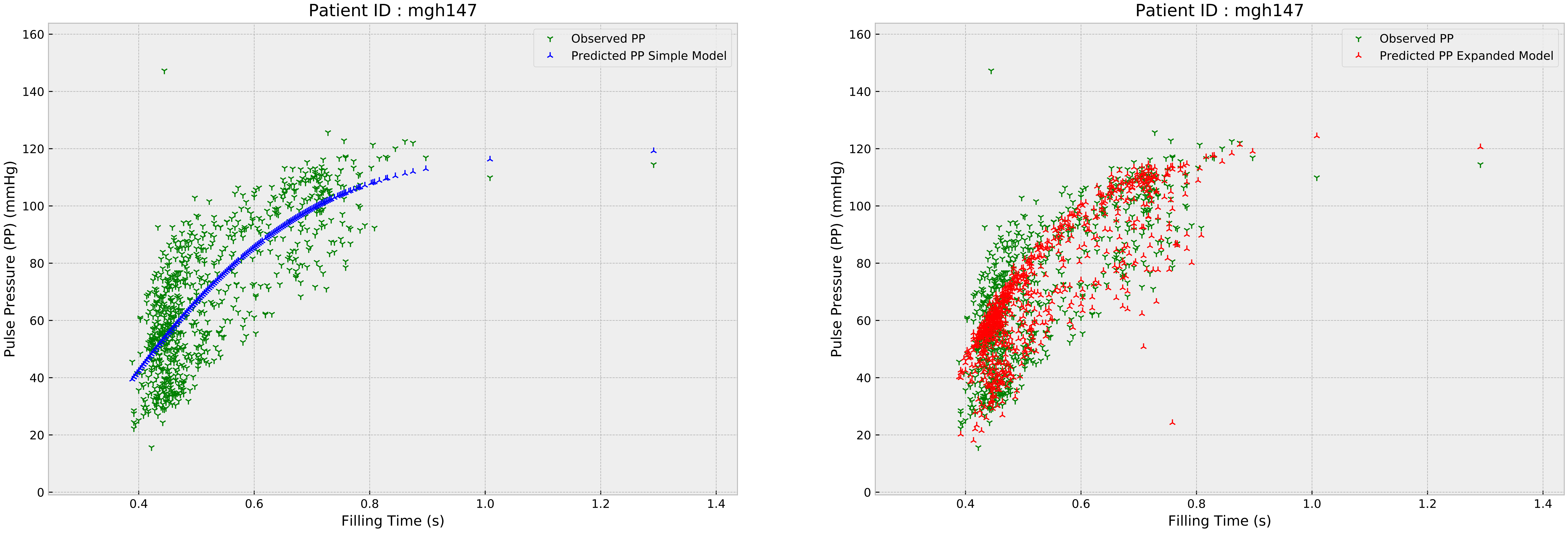

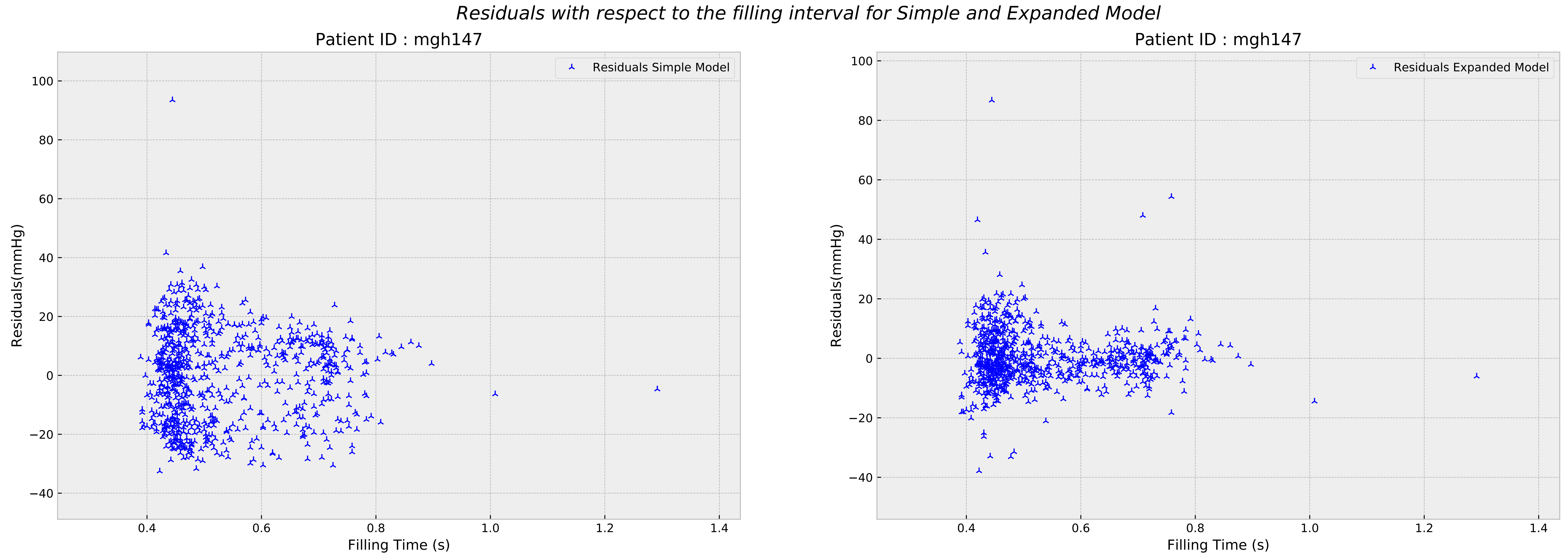
Residuals with respect to the filling interval for Simple and Expanded Model
Patient ID : mgh147
Residuals Simple Model
Residuals(mmHg)
Filling Time (s)
100
80
60
40
20
0
−20
−40
0.4
0.6
0.8
1.0
1.2
1.4
Patient ID : mgh147
Residuals Expanded Model
Residuals(mmHg)
Filling Time (s)
100
80
60
40
20
0
−20
−40
0.4
0.6
0.8
1.0
1.2
1.4

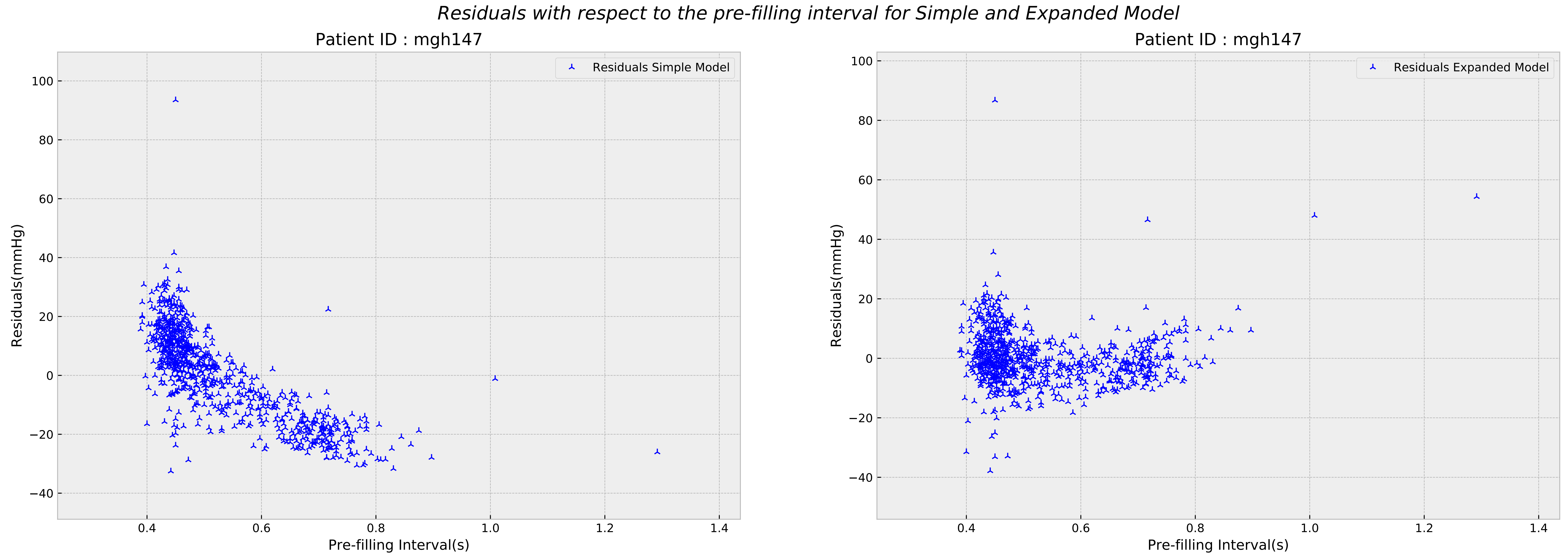
Residuals with respect to the pre-filling interval for Simple and Expanded Model
Patient ID : mgh147
Residuals Simple Model
Residuals(mmHg)
Pre-filling Interval(s)
100
80
60
40
20
0
−20
−40
0.4
0.6
0.8
1.0
1.2
1.4
Patient ID : mgh147
Residuals Expanded Model
Residuals(mmHg)
Pre-filling Interval(s)
100
80
60
40
20
0
−20
−40
0.4
0.6
0.8
1.0
1.2
1.4

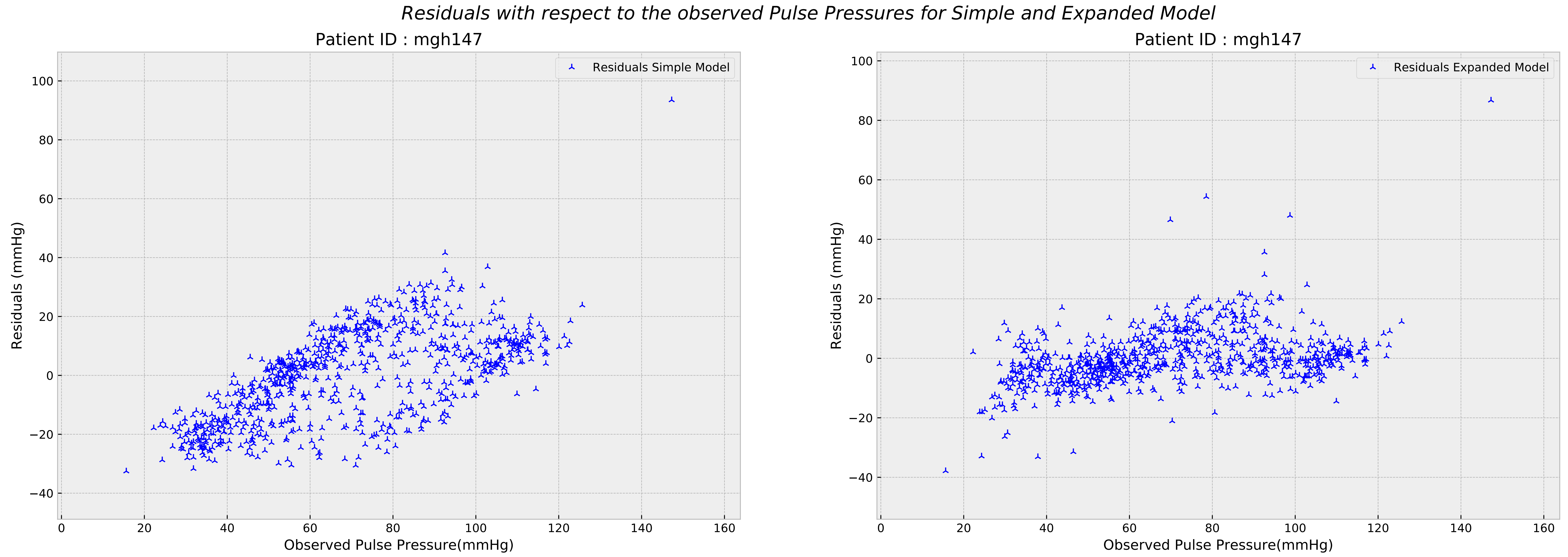

Residuals with respect to the observed Pulse Pressures for Simple and Expanded Model
Patient ID : mgh147
Residuals Simple Model
Residuals (mmHg)
Observed Pulse Pressure(mmHg)
Patient ID : mgh147
Residuals Expanded Model
Residuals (mmHg)
Observed Pulse Pressure(mmHg)

Observed vs. predicted relationship between pulse pressures (PP) and filling times for Simple and Expanded Model

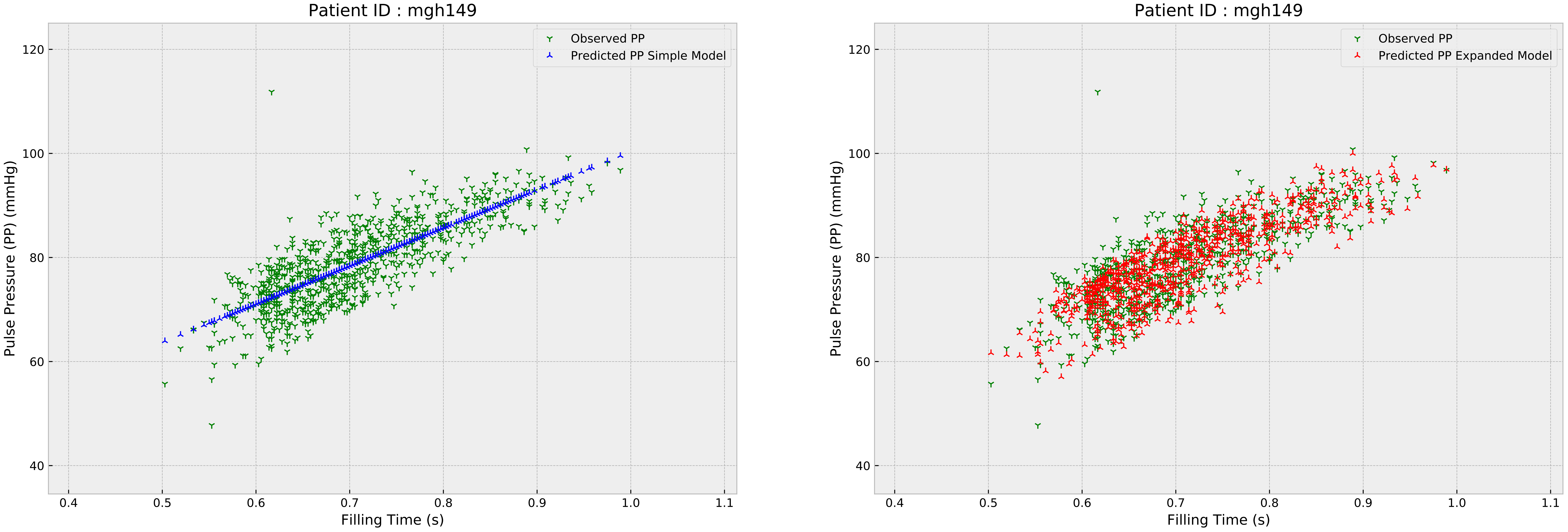

Residuals with respect to the filling interval for Simple and Expanded Model

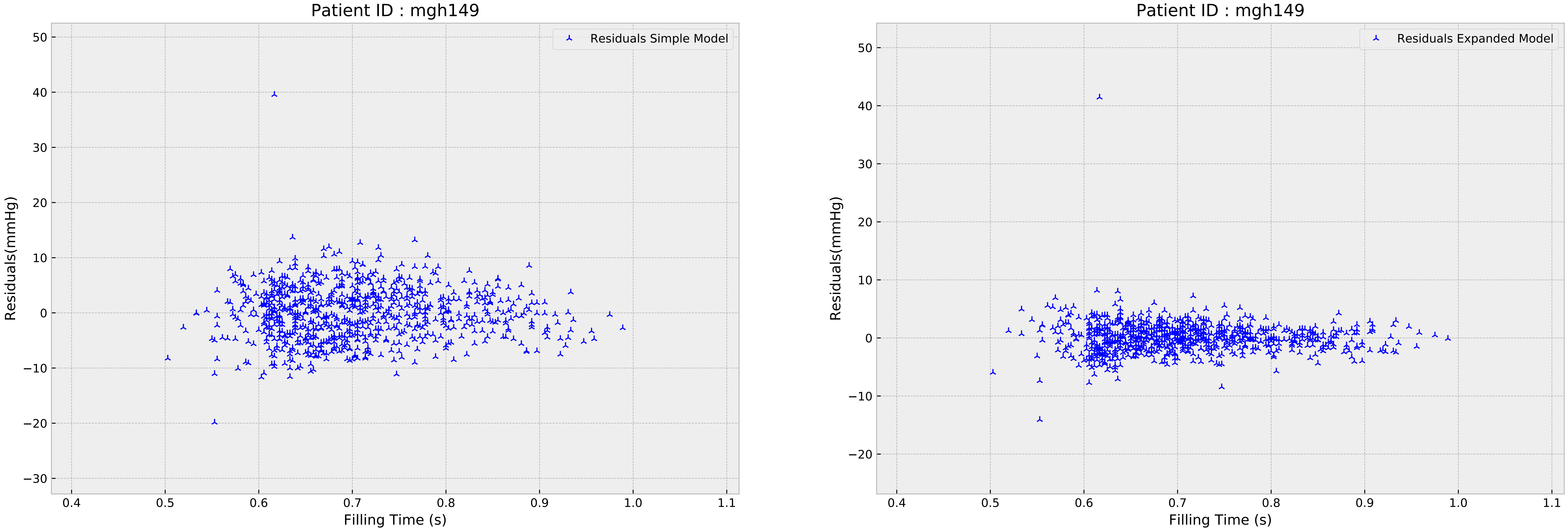

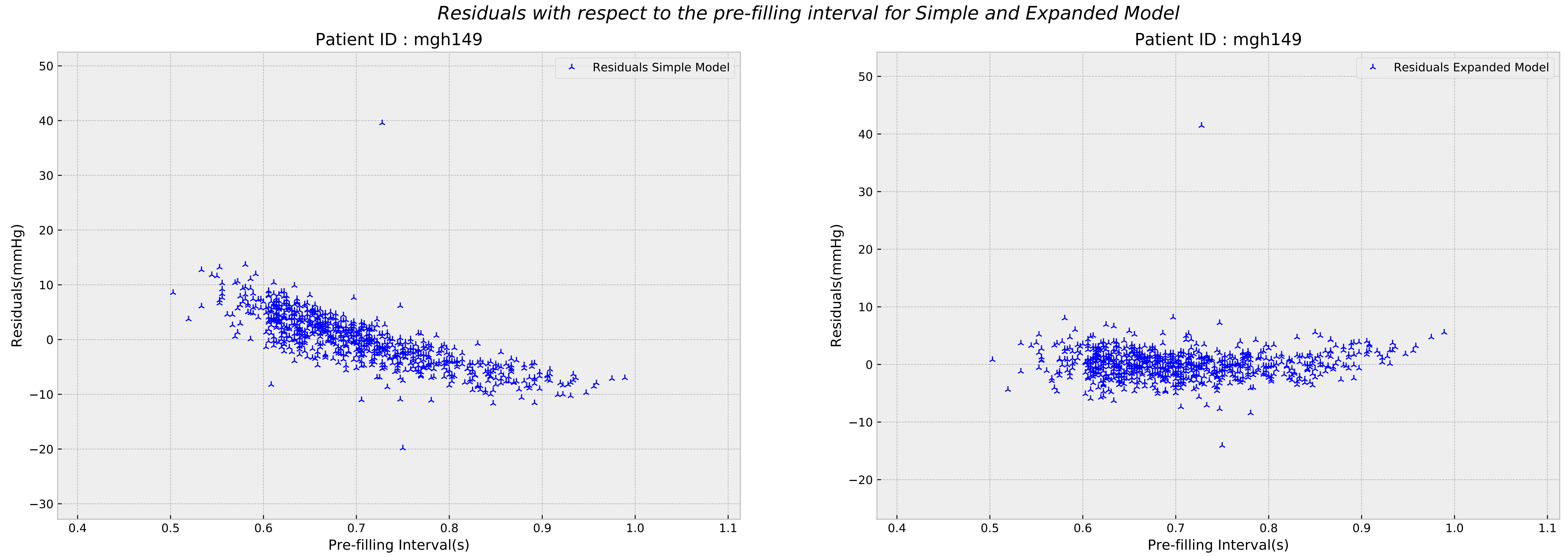
Residuals with respect to the pre-filling interval for Simple and Expanded Model
Patient ID : mgh149
Residuals Simple Model
Residuals(mmHg)
Pre-filling Interval(s)
50
40
30
20
10
0
−10
−20
−30
0.4
0.5
0.6
0.7
0.8
0.9
1.0
1.1
Patient ID : mgh149
Residuals Expanded Model
Residuals(mmHg)
Pre-filling Interval(s)
50
40
30
20
10
0
−10
−20
0.4
0.5
0.6
0.7
0.8
0.9
1.0
1.1

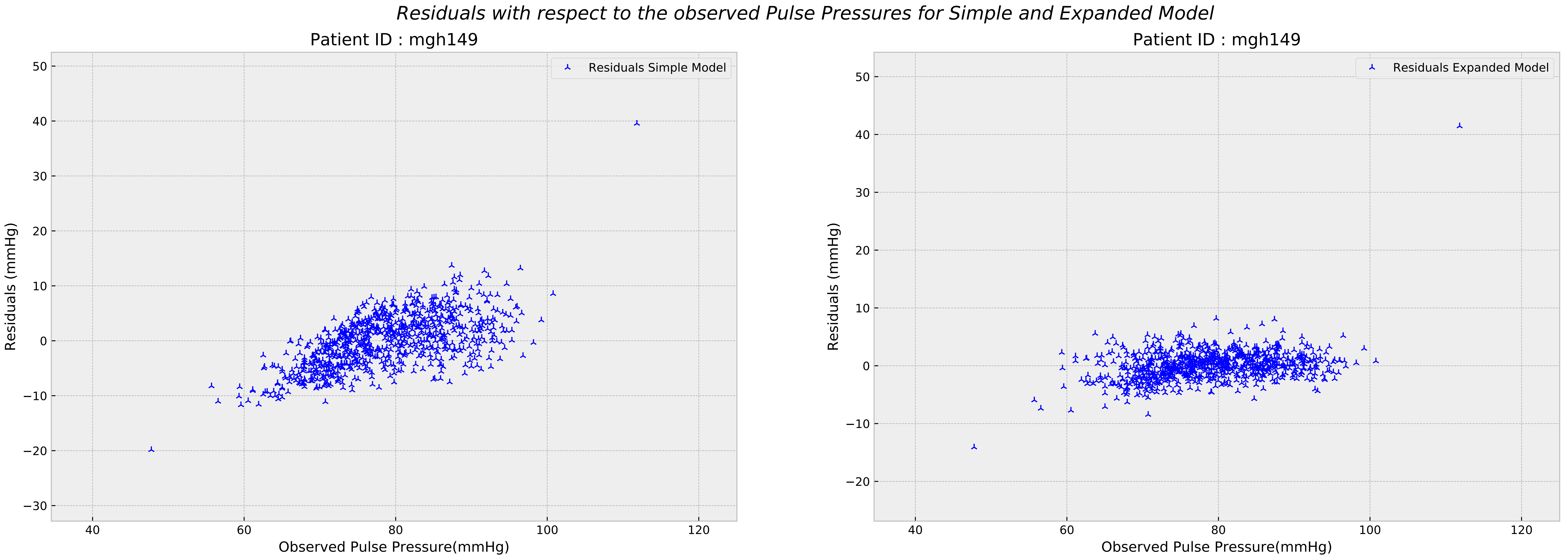
Residuals with respect to the observed Pulse Pressures for Simple and Expanded Model
Patient ID : mgh149
Residuals Simple Model
Residuals (mmHg)
Observed Pulse Pressure(mmHg)
50
40
30
20
10
0
−10
−20
−30
40
60
80
100
120
Patient ID : mgh149
Residuals Expanded Model
Residuals (mmHg)
Observed Pulse Pressure(mmHg)
50
40
30
20
10
0
−10
−20
40
60
80
100
120

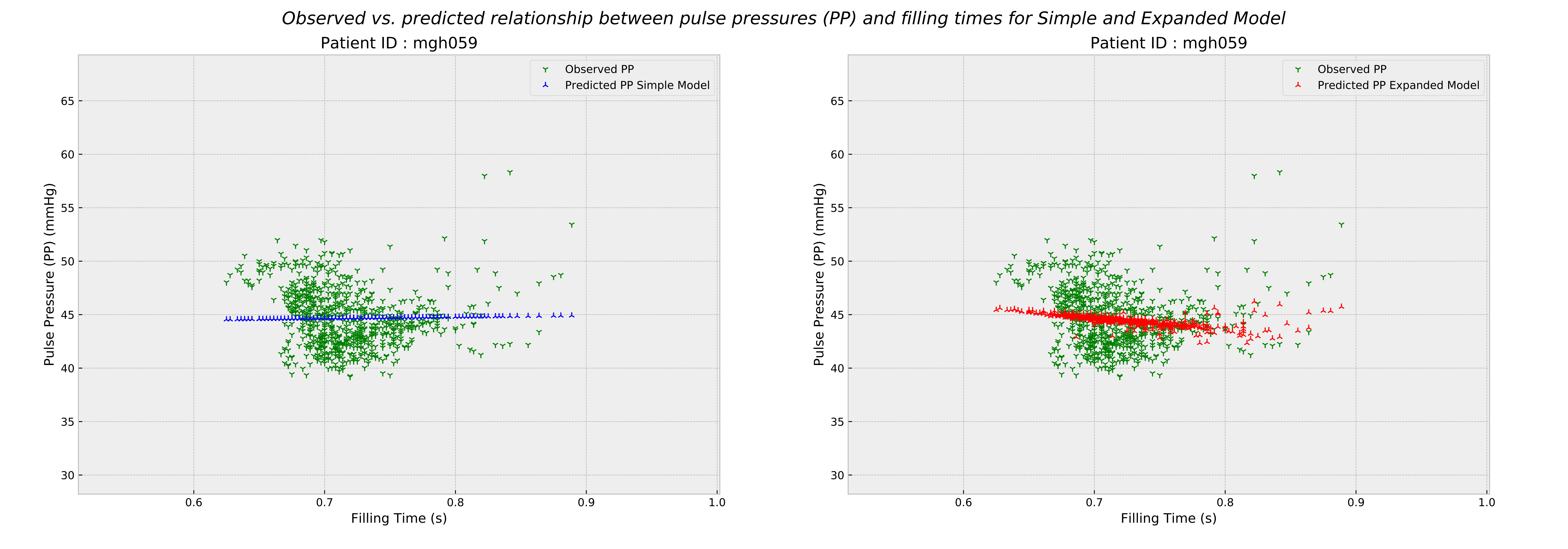

Observed vs. predicted relationship between pulse pressures (PP) and filling times for Simple and Expanded Model
Patient ID : mgh059
Observed PP
Predicted PP Simple Model
Pulse Pressure (PP) (mmHg)
Filling Time (s)
65
60
55
50
45
40
35
30
0.6
0.7
0.8
0.9
1.0
Patient ID : mgh059
Observed PP
Predicted PP Expanded Model
Pulse Pressure (PP) (mmHg)
Filling Time (s)

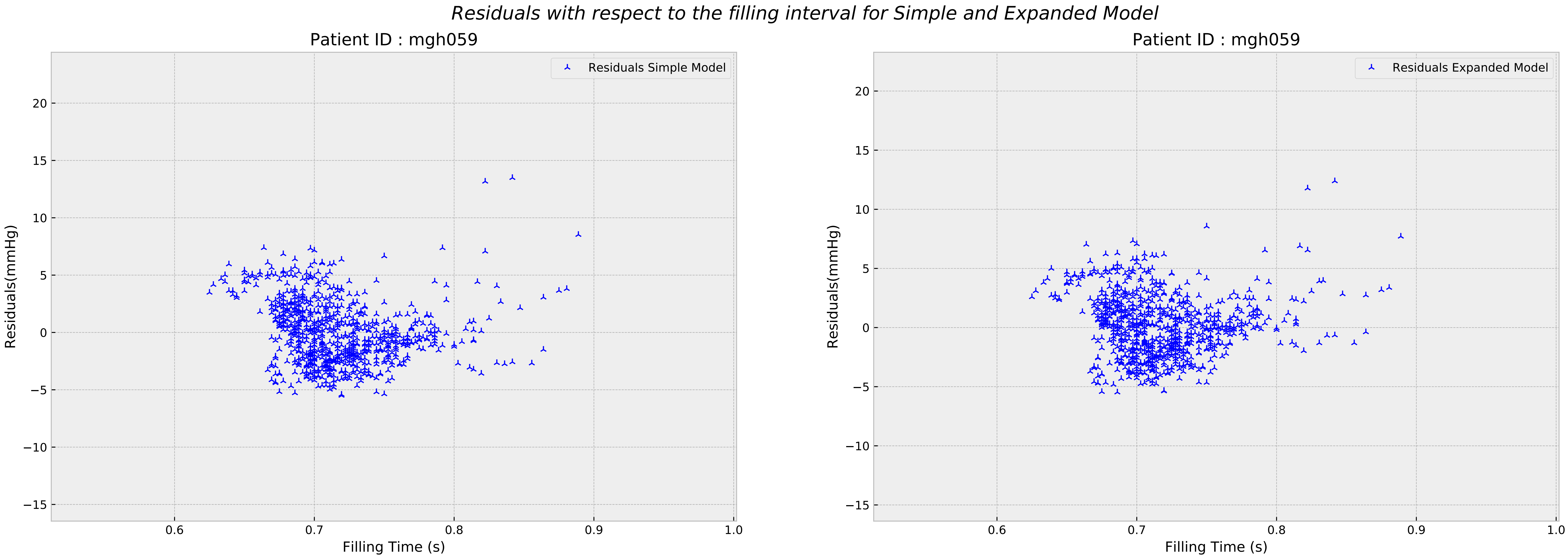
Residuals with respect to the filling interval for Simple and Expanded Model
Patient ID : mgh059
Residuals Simple Model
Residuals(mmHg)
Filling Time (s)
20
15
10
5
0
−5
−10
−15
0.6
0.7
0.8
0.9
1.0
Patient ID : mgh059
Residuals Expanded Model
Residuals(mmHg)
Filling Time (s)

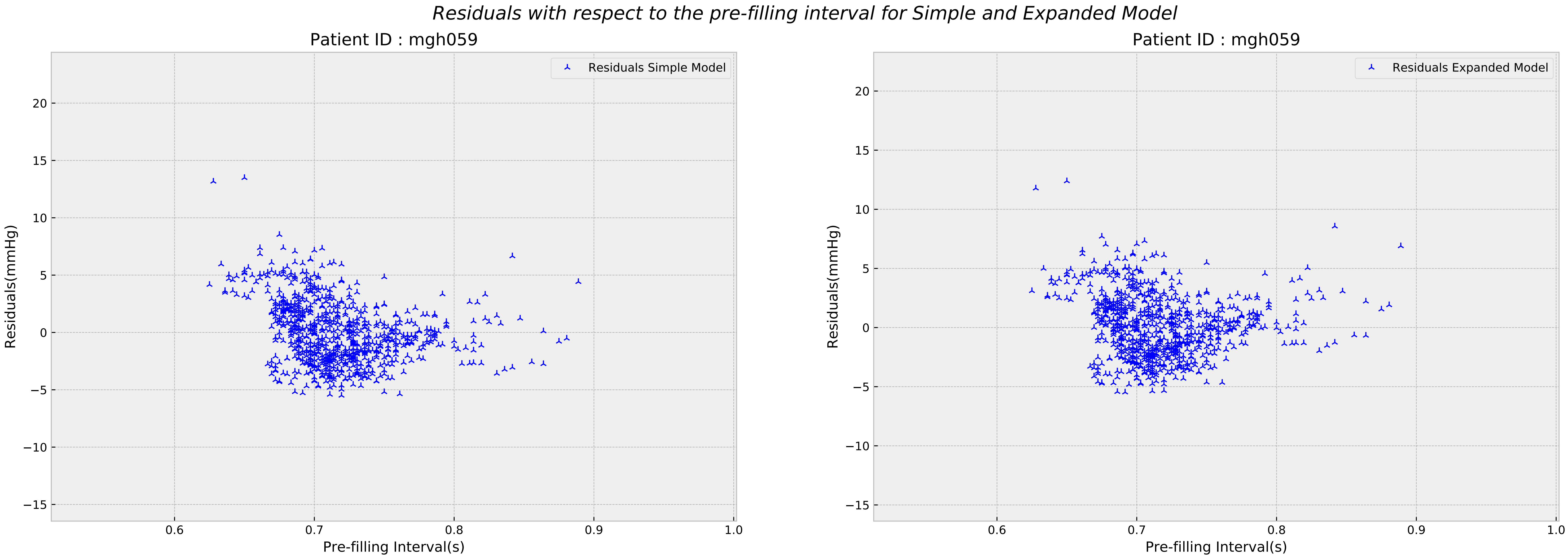
Residuals with respect to the pre-filling interval for Simple and Expanded Model
Patient ID : mgh059
Residuals Simple Model
Residuals(mmHg)
Pre-filling Interval(s)
Patient ID : mgh059
Residuals Expanded Model
Residuals(mmHg)
Pre-filling Interval(s)

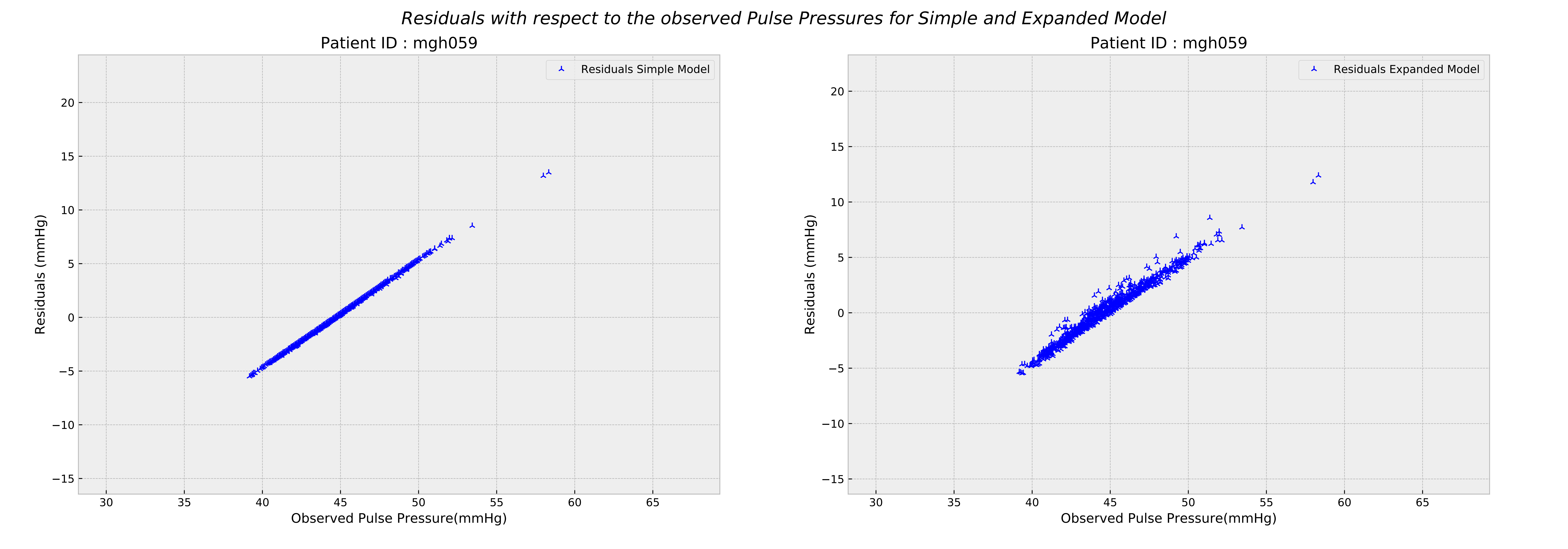
Residuals with respect to the observed Pulse Pressures for Simple and Expanded Model
Patient ID : mgh059
Residuals Simple Model
Residuals (mmHg)
Observed Pulse Pressure(mmHg)
Patient ID : mgh059
Residuals Expanded Model
Residuals (mmHg)
Observed Pulse Pressure(mmHg)

Observed vs. predicted relationship between pulse pressures (PP) and filling times for Simple and Expanded Model

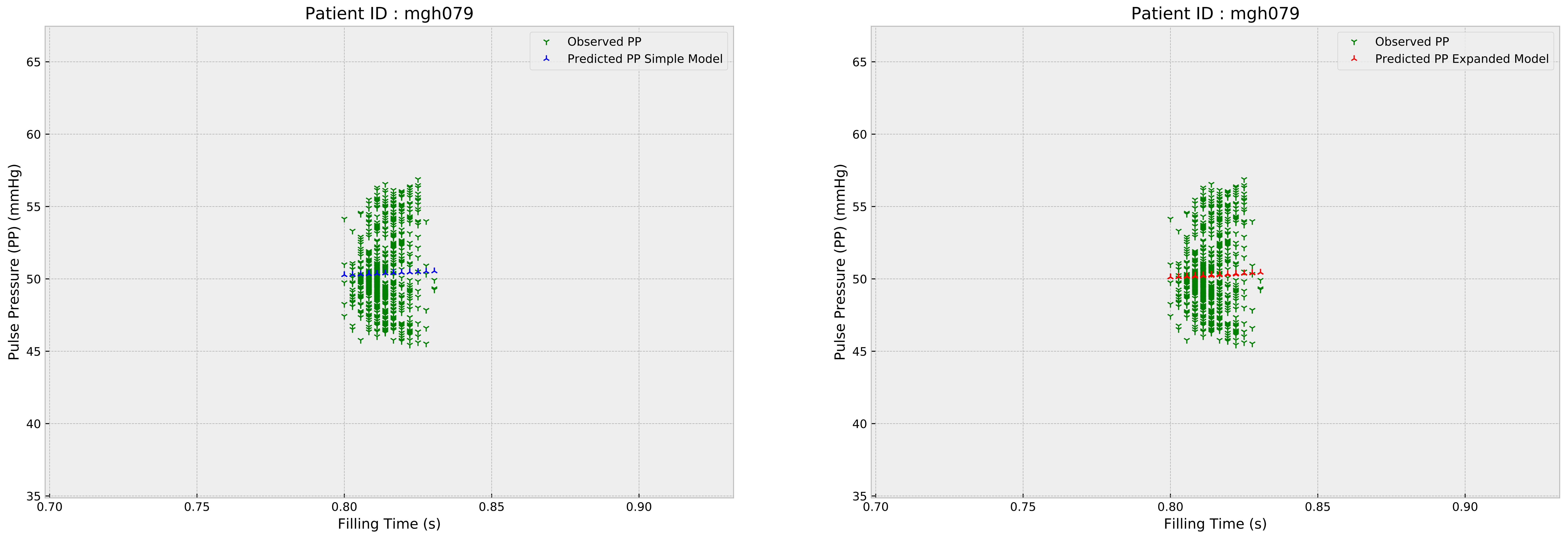

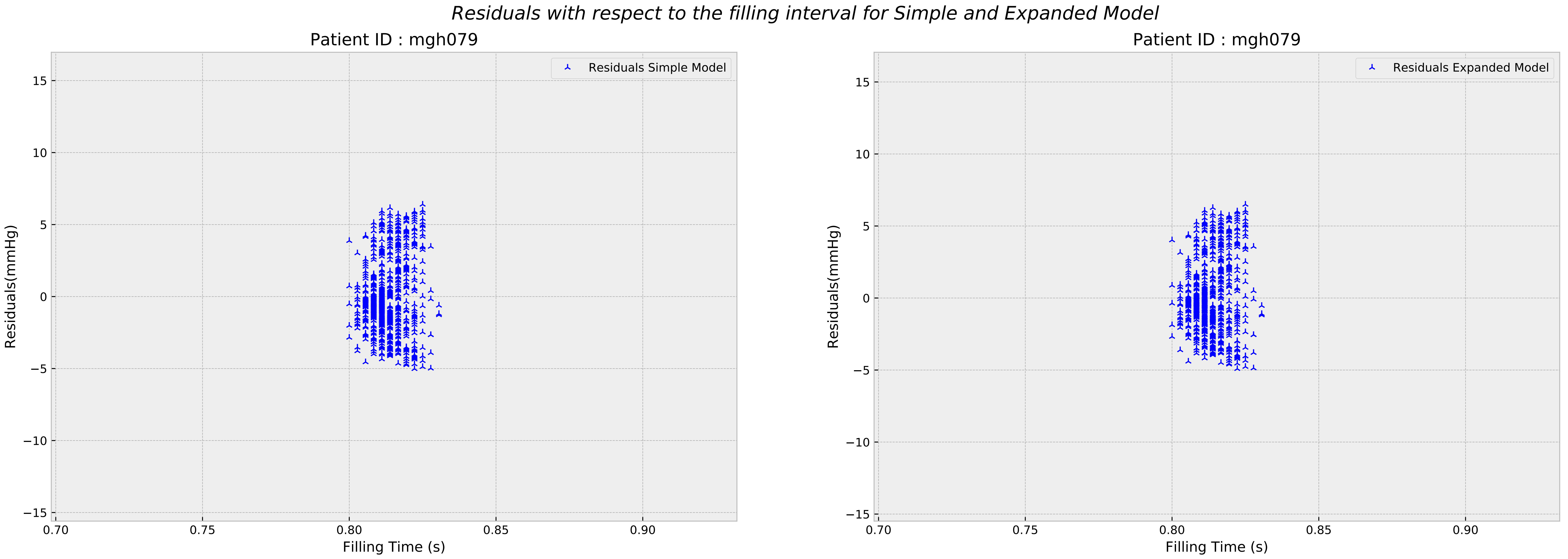
Residuals with respect to the filling interval for Simple and Expanded Model
Patient ID : mgh079
Residuals Simple Model
Residuals(mmHg)
15
10
5
0
−5
−10
−15
0.70
0.75
0.80
0.85
0.90
Filling Time (s)
Patient ID : mgh079
Residuals Expanded Model
Residuals(mmHg)
15
10
5
0
−5
−10
−15
0.70
0.75
0.80
0.85
0.90
Filling Time (s)

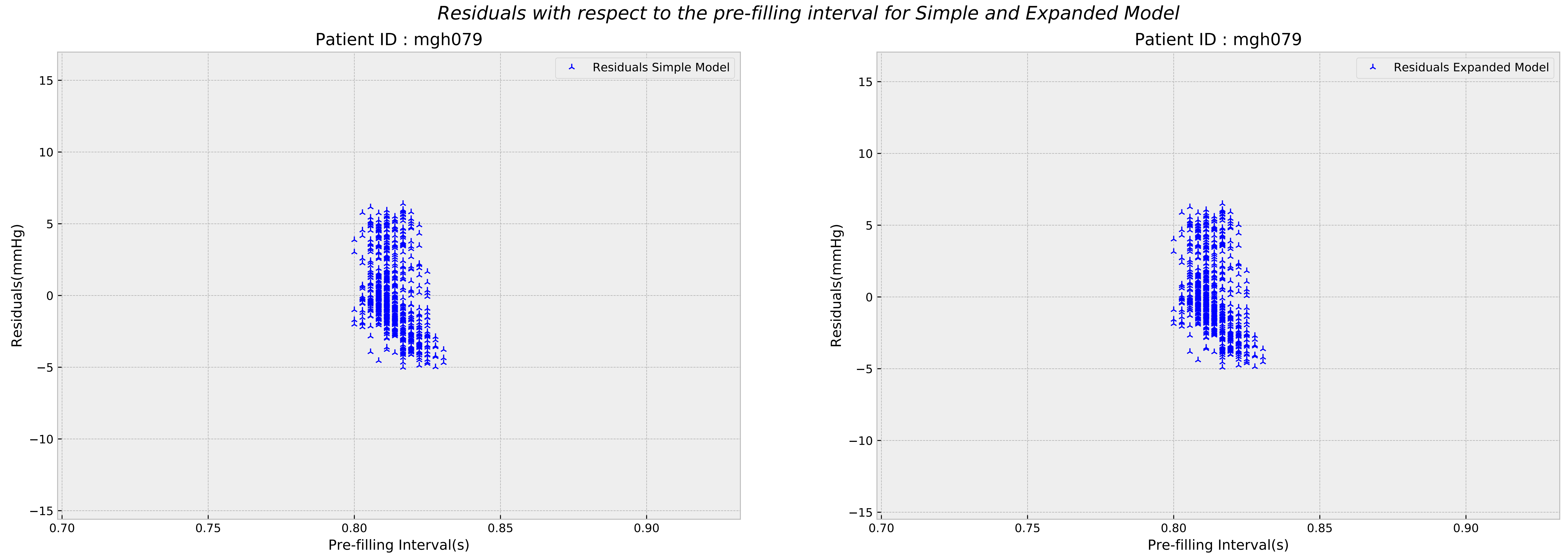
Residuals with respect to the pre-filling interval for Simple and Expanded Model
Patient ID : mgh079
Residuals Simple Model
Residuals(mmHg)
Pre-filling Interval(s)
Patient ID : mgh079
Residuals Expanded Model
Residuals(mmHg)
Pre-filling Interval(s)

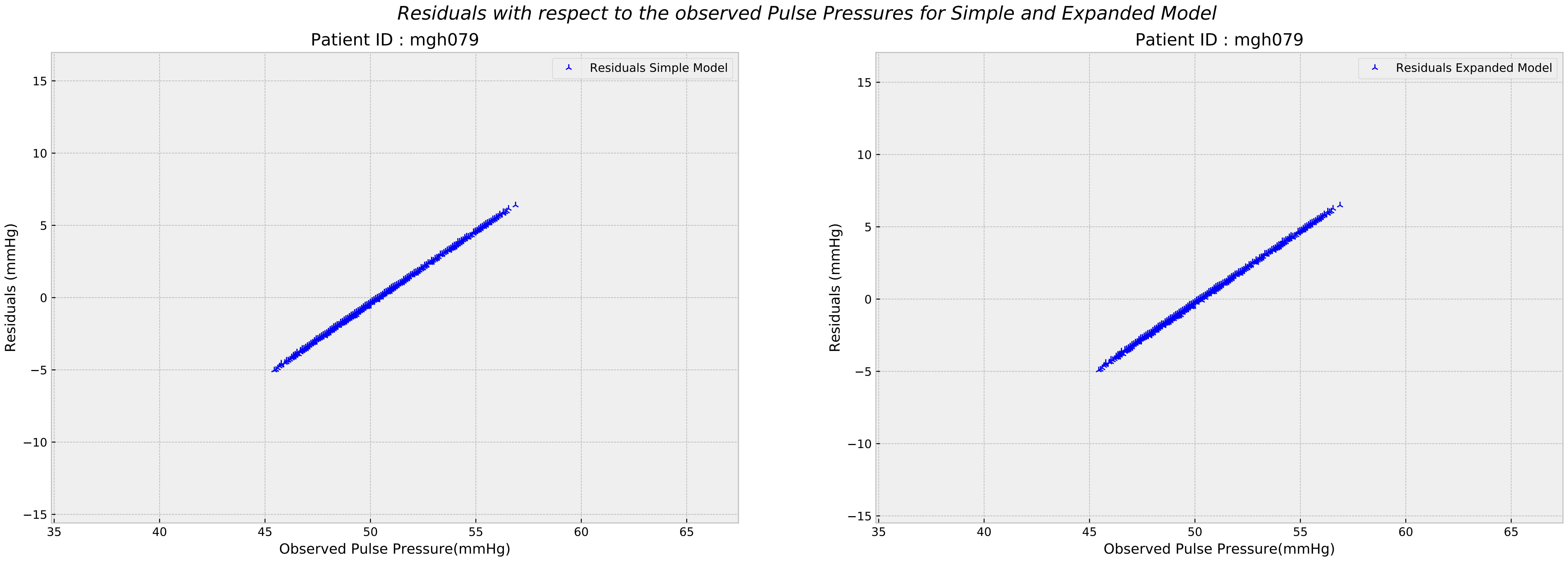
Residuals with respect to the observed Pulse Pressures for Simple and Expanded Model
Patient ID : mgh079
Residuals Simple Model
Residuals (mmHg)
Observed Pulse Pressure(mmHg)
15
10
5
0
−5
−10
−15
35
40
45
50
55
60
65
Patient ID : mgh079
Residuals Expanded Model
Residuals (mmHg)
Observed Pulse Pressure(mmHg)

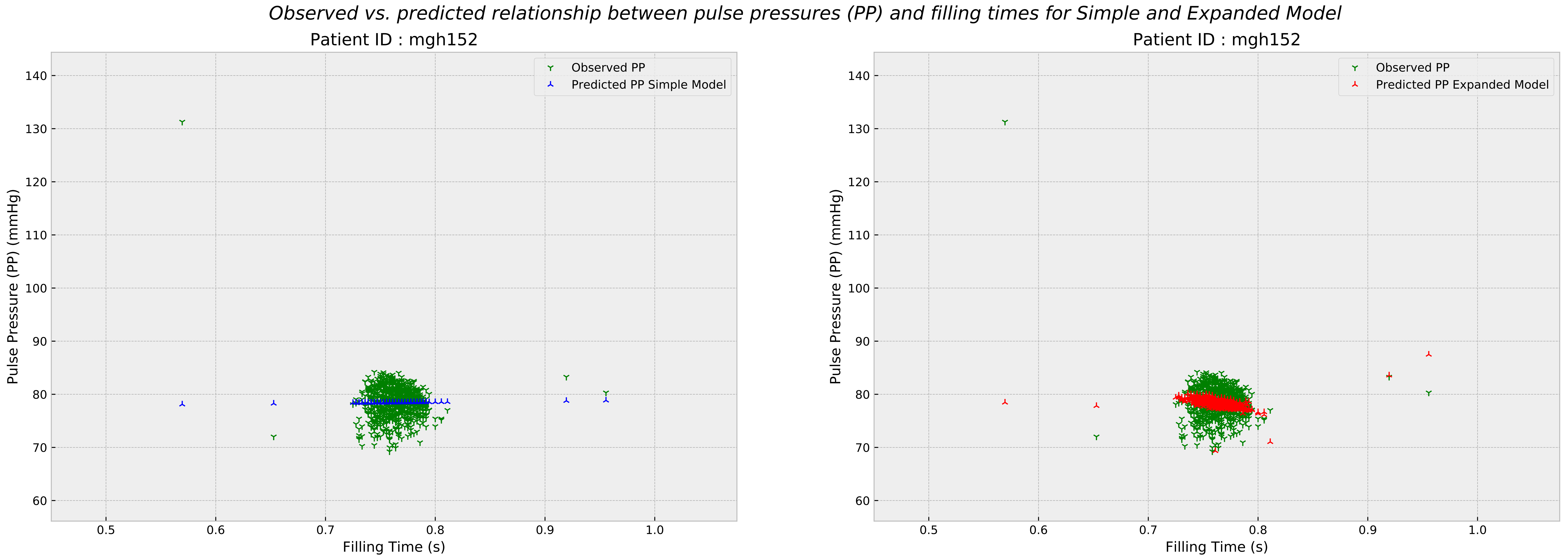

Observed vs. predicted relationship between pulse pressures (PP) and filling times for Simple and Expanded Model
Patient ID : mgh152
Observed PP
Predicted PP Simple Model
Pulse Pressure (PP) (mmHg)
Filling Time (s)
Patient ID : mgh152
Observed PP
Predicted PP Expanded Model
Pulse Pressure (PP) (mmHg)
Filling Time (s)

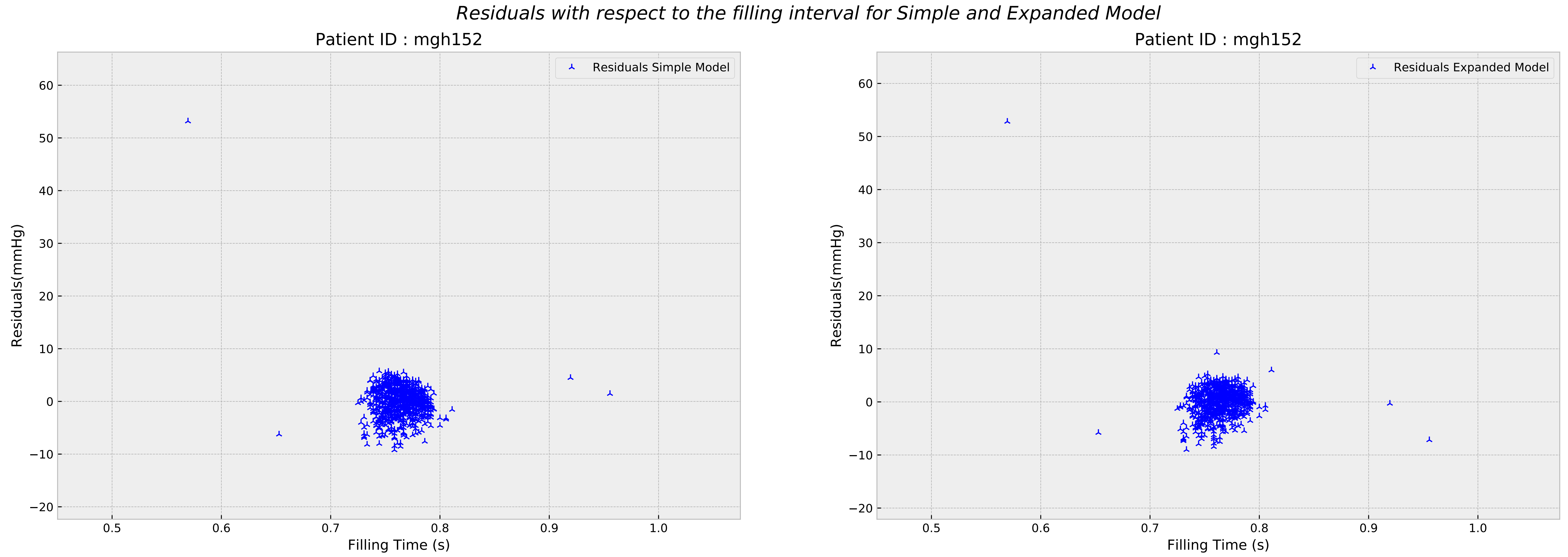
Residuals with respect to the filling interval for Simple and Expanded Model
Patient ID : mgh152
Residuals Simple Model
Residuals(mmHg)
Filling Time (s)
60
50
40
30
20
10
0
−10
−20
0.5
0.6
0.7
0.8
0.9
1.0
Patient ID : mgh152
Residuals Expanded Model
Residuals(mmHg)
Filling Time (s)

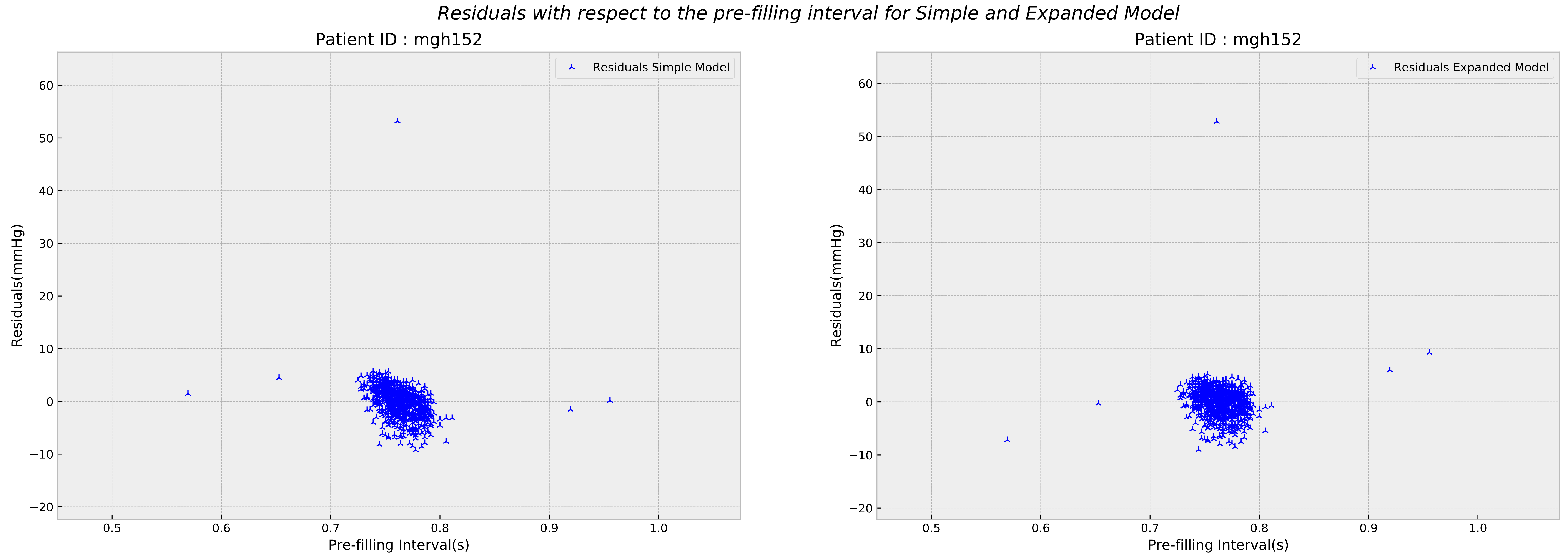

Residuals with respect to the pre-filling interval for Simple and Expanded Model
Patient ID : mgh152
Residuals Simple Model
Residuals(mmHg)
Pre-filling Interval(s)
Patient ID : mgh152
Residuals Expanded Model
Residuals(mmHg)
Pre-filling Interval(s)

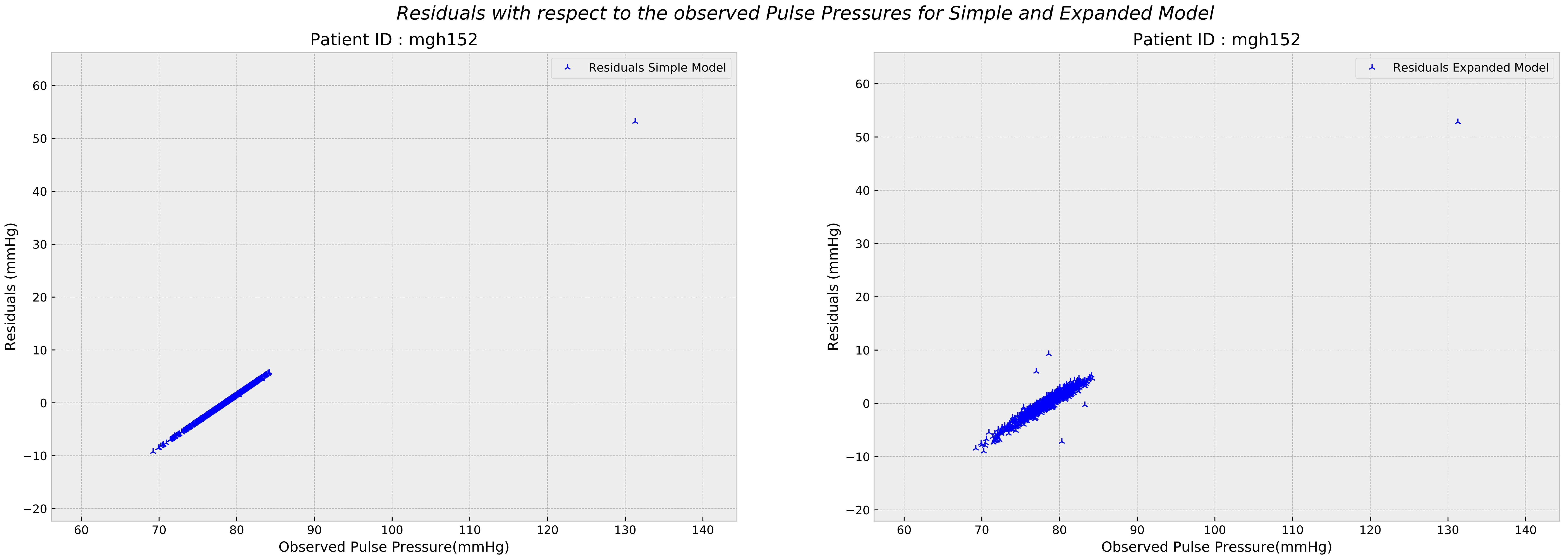
Residuals with respect to the observed Pulse Pressures for Simple and Expanded Model
Patient ID : mgh152
Residuals Simple Model
Residuals (mmHg)
Observed Pulse Pressure(mmHg)
Patient ID : mgh152
Residuals Expanded Model
Residuals (mmHg)
Observed Pulse Pressure(mmHg)
